# Structural Polytypism in B‑Site-Deficient Azetidinium-Based Pnictogen Halide Hexagonal Perovskites

**DOI:** 10.1021/acs.inorgchem.5c01374

**Published:** 2025-06-26

**Authors:** Hang Liu, Rebecca Rae, James Dalzell, Gavin S. Peters, Herbert Früchtl, Aidan P. McKay, David B. Cordes, Amit Kumar, Caroline A. Kirk, Finlay D. Morrison

**Affiliations:** † EaStCHEM School of Chemistry, 7486 University of St Andrews, St Andrews KY16 9ST, U.K.; ‡ EaStCHEM School of Chemistry, 151018 University of Edinburgh, Edinburgh EH9 3FJ, U.K.; § Centre for Quantum Materials and Technologies, School of Mathematics and Physics, 240524 Queen’s University Belfast, Belfast BT7 1NN, U.K.

## Abstract

Azetidinium (Az^+^)-based antimony and bismuth organic–inorganic hybrid halide B-site-deficient perovskite analogues Az_3_B_2_X_9_ (B^3+^ = Sb, Bi; X^–^ = Cl, Br, I) were systematically studied. All Az_3_B_2_X_9_ stoichiometries adopt hexagonal close-packed perovskite structures with the 6H (hcc)_2_ stacking sequence, differing only in the positions of the ordered B-site vacancies. In Az_3_Sb_2_Cl_9_ and Az_3_Sb_2_Br_9_ ordering of B-site vacancies in a single face-sharing octahedral layer leads to the formation of an unusual 2D layered polar structure with the *P*6_3_ *mc* space group. Variable-temperature single-crystal and powder XRD, DSC, DTA, and dielectric spectroscopy showed several successive phase transitions at low temperatures associated with distortions of the octahedral framework and order/disorder of the Az^+^ cation. In contrast, in Az_3_Sb_2_I_9_, Az_3_Bi_2_Br_9_, and Az_3_Bi_2_I_9_, the preferred arrangement of vacancies at corner-sharing octahedral sites generates a 0D “dimer” structure. This work highlights the flexibility for structural variations based on particular configurations of vacancy ordering in B-site-deficient halide perovskites.

## Graphical abstract

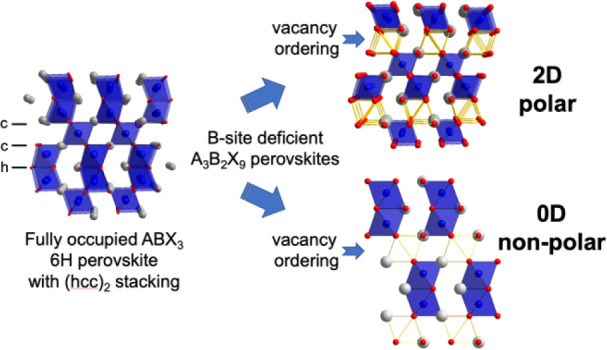

## 1 Introduction

The search for environmentally friendly, novel materials for optoelectronic and photovoltaic applications has become a global trend.^1,2^ Among all candidates, heavy metal halide perovskites with the general formula ABX_3_ (where A represents a large monovalent cation, B denotes a smaller B^2+^ metal cation, and X is a halide anion) have been extensively studied due to their rich structural diversity and tunable electronic properties.^3^ Recently, the search for replacement of lead-containing halide perovskite materials with lead-free organic–inorganic hybrid perovskites (OIHPs), especially halides based on isoelectronic Sb^3+^ and Bi^3+^, has become an inevitable trend.^4–7^ By introducing such higher-valence cations at the B-site in the ABX_3_ structure, the B-site-deficient perovskite family, presented with the general formula: A­(□*_n_* B’_1–3*n*_B_2*n*_)­X_3_ (where □ denotes a vacant site, *n* represents the fraction of vacancies per perovskite formula unit, and B’ and B are di- and trivalent cations, respectively), can be generated.

For general ABX_3_ perovskite polytypes, the crystal structure can be described as a combination of close-packed AX_3_ layers with various stacking sequences and interlayer octahedral voids occupied by B-site cations.^8^ To classify various polytypes based on stacking sequences, the Jagodzinski and Ramsdell notations are commonly utilized to informatively describe the structure.^9^ The Jagodzinski notation describes the structure based on the AX_3_ stacking sequence of hexagonal (h) and cubic (c) close-packing layers, whereas the Ramsdell notation adopts the form *n*M where *n* denotes the number of layers in the aristotype unit cell and M = C, H, and R represents cubic, hexagonal, and rhombohedral symmetry of the aristotype structure. To better understand the structural relationship among B-site-deficient perovskites, an extension of the description methods from stoichiometric ABX_3_ perovskites to nonstoichiometric B-site-deficient perovskites has been provided as a useful tool in the study of inorganic 3C (c)_3_ A_3_B_2_X_9_ halide perovskites.^10^ In this study, we extend these notation methods to other B-site-deficient polytypes other than previously studied cubic polytypes.^11^

Although B-site-deficient oxide perovskites are well-known and have been the subject of numerous studies (see the recent review by Yang et al.^12^) in recent years, several new B-site-deficient halide perovskite structures have been reported with the general formulas: A_3_B_2_X_9_, A_4_B’B_2_X_12_, A_8_Au_4_BX_23_, and A_10_B’B_6_X_30_, as summarized in Table 1.^13–17^ The underlying structures of all the above families can be described by the combination of Jagodzinski and Ramsdell notations (also included in Table 1). The rich structural diversity and varied octahedral connectivity in B-site-deficient perovskites provide huge flexibility to tailor various properties. For example, the manipulation of the relative composition of B’^2+^ and B^3+^ in A_4_B’B_2_X_12_ changes the original Cs_4_MnSb_2_Cl_12_ from 2D to 1D Cs_10_MnSb_6_Cl_30_ with an improvement in the photoluminescence quantum yield.^16^ Furthermore, by incorporating Cu at the Mn site, Cs_4_Mn_1–*x*_Cu_*x*_Sb_2_Cl_12_, the band gap can be tuned between 3.0 and 1.0 eV, which provides a promising candidate for tandem solar cell applications.^6^ However, the current understanding of B-site-deficient perovskites is still limited. On one hand, the conventional Goldschmidt structural factor has only limited applicability to B-site-deficient perovskites, addressing only cubic close-packed structures and failing to predict any polytypes with hexagonal or mixed cubic and hexagonal close packing.^18–20^ On the other hand, the emergence of novel structures with various ordering of B-site vacancies increases the difficulty of predicting new structures based only on ionic radii.

**Table 1:** Summary of Reported B-Site-Deficient Compounds of Various Halide Perovskite Polytypes with Trivalent or Mixed Di- and Trivalent B-Site Cations, Including Stacking Sequences, Symmetry, and Motif of B-Site Octahedral Connectivity

Polytype and stacking mode	Space group	Typical structure^a^	Octahedral motif
2H, (h)_2_	*R*3̅*c*	Cs_3_Tl_2_Cl_9_ ^21^	^0^[B_2_X_9_]^3–^
	*P*6_3_/*mmc*	Cs_3_Fe_2_F_9_ ^22^	
	*P*6_3_*cm*	Cs_3_Ga_2_F_9_ ^23^	
3C, (c)_3_	*P*3̅*m*1	Cs_3_Bi_2_Br_9_ ^24^	[∞2B2X9]3−
	*C*2/*c*	(GU)_3_Sb_2_Cl_9_ ^25^	
	*R*3̅*m*	Cs_4_MnSb_2_Cl_12_ ^6^	[∞2B′B2X12]4−
	*C*2/*m*	Cs_4_CuSb_2_Cl_12_ ^26^	
	*Pm*3̅*m*	Cs_8_Au_4_InBr_23_ ^15^	[∞3Au4BX24]8−
	*I*4_1_/*amd*	K_4_Mn_3_F_12_ ^27^	[∞3B′B2X12]4−
6H, (hcc)_2_	*P*6_3_/*mmc*	Cs_3_Cr_2_Cl_9_ ^28^	^0^[B_2_X_9_]^3–^
	*C*2/*c*	(MA)_3_Bi_2_I_9_ ^29^	
	*P*6_3_*mc*	(Az)_3_Bi_2_I_9_ ^30^	
	*Cmc*2_1_	(Az)_3_Bi_2_Br_9_ ^30^	
	*Pnma*	Cs_3_Bi_2_Cl_9_ ^31^	[∞1B4X18]6−
	*P*2_1_/*n*	(DMA)_3_Bi_2_Cl_9_ ^32^	
	*P*6_3_*mc*	(Az)_3_Sb_2_Br_9_ ^33^	[∞2B2X9]3−
	*P*31*c*	(Az)_3_Bi_2_Cl_9_ ^30^	
9R, (hhc)_3_	*R*3*m*	(PY)_3_Sb_2_Cl_9_ ^34^	[∞2B2X9]3−
10H, (hcccc)_2_	*Pnnm*	Cs_10_MnSb_6_Cl_30_ ^16^	[∞1B′B6X30]10−
12R, (hhcc)_3_	*R*3̅*m*	K_4_Fe_3_F_12_ ^35^	[∞2B′B2X12]4−

a GU denotes the guanidinium (CH_6_N_3_^+^) cation, MA represents the methylammonium (CH_3_NH_3_^+^) cation, FA denotes the formamidinium (CH_5_N_2_^+^) cation, DMA denotes the dimethylammonium (C_2_H_5_NH_3_^+^) cation, Az represents the azetidinium (C_3_H_8_N^+^) cation, and PY represents the pyrrolidinium (C_4_H_8_NH_2_^+^) cation.

One possible tool to investigate the potential stable polytypes is by extending the known Bärnighausen tree for B-site-deficient perovskite variants by using the Bilbao Crystallographic Server.^36–41^ By varying the crystallographic positions of sites occupied by B-site cations and vacancies, various hypothesized B-site-deficient perovskite structures can be generated and studied by different methods to evaluate their thermodynamic stability.^42^ This method has been extended by Chang et al. to describe all possible inorganic 3C B-site-deficient A_3_B_2_X_9_ structures.^10^ In a similar manner, the crystallographic relationships among 6H B-site-deficient A_3_B_2_X_9_ variants can also be described by following all possible paths from the parent fully occupied aristotype 6H ABX_3_ perovskite structure (with *P*6_3_/*mmc* symmetry), as the simplified schematic in Figure 1 demonstrates (the full Bärnighausen tree can be found in Figure S4.1). Based on group–subgroup relationships, the reported three different 6H A_3_B_2_X_9_ families can be distinguished as 6H 2D [∞2B2X9]3− (with aristotype *P*6_3_ *mc* symmetry), 6H 1D [∞1B4X18]6− (*Pnma*) and 6H 0D ^0^[B_2_X_9_]^3–^ (*P*6_3_/*mmc*). Compared to the 3C A_3_B_2_X_9_ family with only a 2D layered aristotype, the distribution of B-site vacancies brings greater influence on the dimensionality and modes of BX_6_ octahedra, ranging from 2D layered to 0D dimer structures. Furthermore, the specific ordering of the A-site organic cations and octahedral distortion also generates interesting properties, e.g., ferroelectric phase transitions, among the different 6H motifs.^32,43,44^ To better understand the structural relationships among different 6H motifs, an in depth study of the possible A_3_B_2_X_9_ structures with 6H motifs would be of great benefit in the exploration of novel low-dimensional halide perovskites.

**Figure 1:**
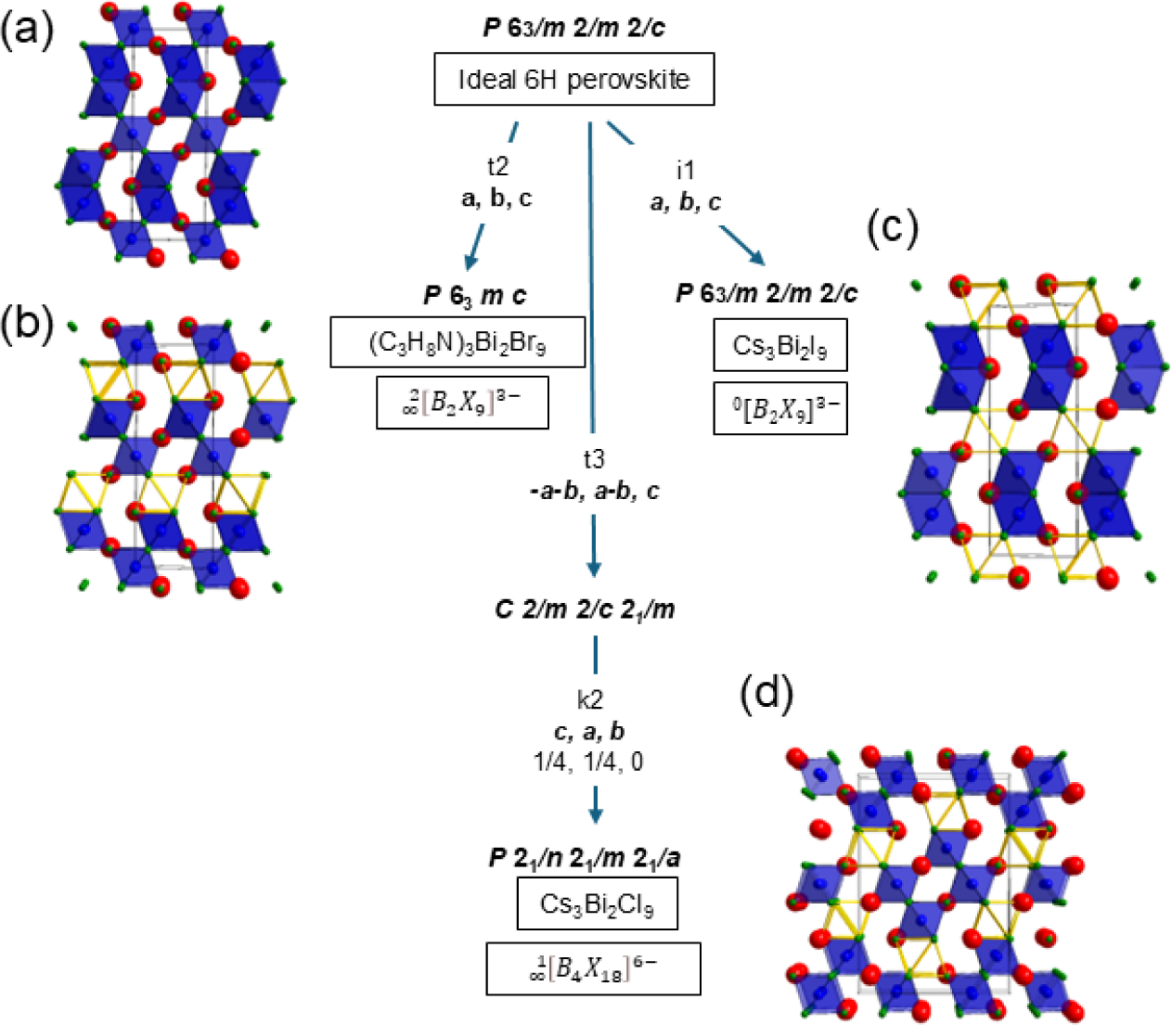
Simplified Bärnighausen tree for the group–subgroup relationships for B-site-deficient close-packed perovskite A_3_B_2_X_9_ structures derived from the fully occupied 6H aristotype (a). The various vacancy ordering results in either (b) 2D layered, (c) 0D ″dimer”, or (d) 1D columnar octahedral connectivity. Subgroups are determined from ideal positions where t represents translation equivalent subgroups, k denotes point group equivalent subgroups, and i denotes the isomorphic structure (for full version with site symmetry relationships, see Figure S4.1). Solid blue and yellow outlined octahedra indicate occupied and vacant B-sites, respectively.

In this paper, we report the systematic study of azetidinium (Az^+^, C_3_H_8_N^+^) pnictogen hybrid halide perovskites with the target composition Az_3_B_2_X_9_ where B = Sb^3+^ and Bi^3+^ and X = Cl^–^, Br^–^, and I^–^. These combinations variously form perovskite polytypes based on the 6H framework, but differing vacancy ordering results in either 2D layered or 0D perovskite structures. However, in two cases, a competing nonperovskite Az_2_BiX_5_ *cis*-chain structure is obtained. Here, we give an overview of the different composition-structure–property combinations of the perovskite phases in relation to the parent fully occupied 6H polytype, using both experimental and computational methods, and include comparisons with previous studies.

## 2 Experimental Methods

### 2.1 Sample Preparation

Starting materials involved azetidine (98%, Apollo Scientific), Bi_2_O_3_ (99%, Alfa Aesar), BiCl_3_ (98%, Fluorochem), BiBr_3_ (99%, Alfa Aesar), BiI_3_ (99%, Sigma-Aldrich), SbCl_3_ (99%, Alfa Aesar), SbBr_3_ (99%, Alfa Aesar), SbI_3_ (98%, Sigma-Aldrich), HCl_(aq)_ (37%, Sigma-Aldrich), HBr_(aq)_ (48%, Alfa Aesar), HI_(aq)_ (55–58%, Alfa Aesar), and 2-propanol (99.5%, Sigma-Aldrich). All were used as purchased without further purification.

Powder samples were prepared by a conventional solution method. For the preparation of Az_3_Sb_2_X_9_ and Az_3_Bi_2_I_9_, metal halides (4 mmol of SbCl_3_, SbBr_3_, SbI_3_, BiI_3_) were weighed and dissolved in the corresponding dilute acid (5 mL) in an ice bath with continuous magnetic stirring. The azetidine (3 mmol, ca. 0.2 mL) was dissolved in deionized water (2 mL) in an ice bath to inhibit the mass loss caused by the exothermic dissolution. Subsequently, the powder sample was obtained by slowly adding the azetidine aqueous solution dropwise to the metal halide precursor with continuous magnetic stirring; precipitation of the powder sample occurred within a few minutes. The yielded product was separated by vacuum filtration and washed several times with small portions (∼20 mL) of isopropanol. For the preparation of Az_2_BiCl_5_ powder, after the azetidine was fully mixed with the metal halide solution, 3 mL of isopropanol as an antisolvent was required to precipitate the solids. Due to similar chemical potential between Az_2_BiBr_5_ and Az_3_Bi_2_Br_9_, the synthesis adopted a nonstoichiometric ratio to prevent the coprecipitation of both phases. For Az_2_BiBr_5_, the precursor metal halide solution was obtained by dissolving BiBr_3_ (1 mmol) in 2 mL of 48% conc. HBr. However, for Az_3_Bi_2_Br_9_, the precursor was prepared by filtering a 3 mL suspension containing an excess (3 mmol) of Bi_2_O_3_ and ca. 28.7% w/w HBr_(aq)_ using a glass syringe filter to obtain a BiBr_3_ solution. Then, the following steps to obtain the powder sample were carried out in a similar way to Az_2_BiCl_5_ by mixing the precursors and adding 2-propanol as an antisolvent. After synthesis, filtration, and washing, all samples were dried in a drying oven at 80 °C for 2 h and then stored in a vacuum desiccator.

Single-crystal samples were prepared by a reactant diffusion method. The metal halide precursor was prepared following the same procedure as that used for the solution method and then sealed into a small vial. Azetidine liquid (2 mmol, ca. 0.2 mL) was dissolved in the antisolvent isopropanol and then sealed into a larger vial. Single crystals were grown by vapor-diffusing azetidine and isopropanol into the metal halide solution in a well-sealed system.

Powder samples of solid solutions Az_3_Sb_2_Cl_9–*x*_Br_*x*_ (*x* = 0, 0.5, 1, 1.5, 2, 3, 4.5, 6, 7, 8, and 9) were synthesized by the solution method in a procedure similar to that used for undoped Az_3_Sb_2_Cl_9_ and Az_3_Sb_2_Br_9_. In the preparation, the molar ratio between Cl and Br was controlled by fixing the molarity of Sb^3+^ and adjusting the relative amounts of SbCl_3_, SbBr_3_, HCl, and HBr, as shown in Table S1.1. For most preparations, except Az_3_Sb_2_Cl_7_Br_2_ (s), stoichiometric amounts of antimony halides were dissolved in the corresponding dilute acid (5 mL) in an ice bath with continuous stirring to obtain the precursor solution. Azetidine (0.2 mL), dissolved in 2 mL of deionized water, was subsequently added dropwise into the antimony precursor solution to obtain crystalline precipitates. Due to the initial failed attempt in obtaining single-phase Az_3_Sb_2_Cl_6_Br_3_, a second sample, Az_3_Sb_2_Cl_7_Br_2_ (s), was synthesized using a tripled amount of raw material, as shown in Table S1.1. After vacuum filtering, crystals ranging from transparent to yellowish in color were isolated and dried at 80 °C for 2 h.

In all reactions, the azetidine aqueous solution (∼2 mL) was first prepared by using a stoichiometric amount of azetidine (∼0.2 mL) to mitigate the influence of exothermic heat from the dissolution of azetidine. The azetidine aqueous solution (∼2 mL) was then added dropwise into the metal halide solution with continuous magnetic stirring to obtain powder samples. The time for precipitation depended on the solubility of the yielded products. For Az_3_Sb_2_I_9_, Az_3_Sb_2_Br_9_, and Az_3_Bi_2_I_9_, the precipitation occurs almost instantaneously. For Az_3_Sb_2_Cl_9_ and Az_3_Bi_2_Br_9_, the precipitation normally occurs a few minutes after the addition of the azetidine aqueous solution. For the solid solution series, the time required for precipitation depends on the practical Br concentration of the metal halide solution. Higher Br concentration leads to faster precipitation.

### 2.2 Single Crystal and Powder X-ray Diffraction

Single-crystal X-ray diffraction (SCXRD) data for Az_3_Sb_2_X_9_ (X = Cl, Br, or I), Az_3_Bi_2_X_9_ (X = Br or I), and Az_2_BiX_5_ (X = Cl or Br) compounds were collected using a Rigaku FR-X Ultrahigh Brilliance Microfocus RA generator/confocal optics and an XtaLAB P200 diffractometer [Mo Kα radiation (λ = 0.71073 Å)]. Room-temperature powder X-ray diffraction (PXRD) was performed using a PANalytical Empyrean diffractometer with Cu Kα1 (λ = 1.5406 Å) and a 2θ angle ranging from 3° to 70° at 298 K. The instrument was operated in a Bragg–Brentano geometry with a step size of ca. 0.02°. Low-temperature PXRD data were collected on undoped Az_3_Sb_2_Cl_9_ and Az_3_Sb_2_Br_9_ samples to study the low-temperature crystal structures. A Rigaku Smartlab X-ray diffractometer with Cu Kα1 radiation (λ = 1.5406 Å) was operated in Bragg–Brentano geometry with a step size of 0.01°. Data were collected over a 2θ range of 5° to 70° with a scan speed of 0.2°/min and a temperature range of 20 to 298 K. The temperature was varied using an Oxford Cryosystems PheniX attachment, which uses a recirculating helium compressor. Samples were packed onto a chromium-plated copper sample holder by using silicone grease. Details of the data analysis methodology are given in Sections S2.1 and S2.2.

### 2.3 Compositional Analysis

Determination of the chemical composition in Az_3_Sb_2_Cl_9–*x*_Br_*x*_ was performed on the powder sample via energy-dispersive X-ray spectroscopy (EDS) using a JEOL JSM-IT200 scanning electron microscope (SEM) with an accelerating voltage of 15 kV.

### 2.4 Ultraviolet–Visible Absorbance Spectroscopy

Pseudoabsorbances were estimated from steady-state UV–vis diffuse reflectance data measured by using a JASCO-V650 double-beam spectrophotometer.

### 2.5 Dielectric Spectroscopy

Dielectric spectroscopy was carried out using a 100 mV AC excitation in the frequency range of 100 Hz to 10 MHz with an Agilent 4294A Impedance Analyzer, between 20 and 298 K.

### 2.6 Thermal Analysis

Simultaneous thermogravimetric and differential scanning calorimetry analysis (TG-DSC) was carried out on the powder sample by using a NETZSCH STA449 instrument. The samples were heated at a rate of 10 K min^–1^ from ambient temperature to 673 K. Inert argon or nitrogen was used as the protective and purge gas; the flow rates of the purge and protective gases were 50 and 20 mL min^–1^, respectively. A NETZSCH DSC204F1 was used for the low-temperature differential scanning calorimetry analysis. The samples were heated from room temperature to 323 K, then cooled to 103 K before being heated again to 323 K at 10 K min^–1^ with an N_2_ purge and a protective gas flow rate of 40 mL min^–1^.

### 2.7 Piezoresponse Force Microscopy

PFM measurements were carried out using the MFP-3D Infinity system with Pt/Ir-coated Si cantilevers in conjunction with an SR830 lock-in amplifier. An AC bias, fixed at 20 kHz, was additionally supplied to the probing tip using the same lock-in amplifier for voltages ranging from 1–5 V_ac_. More details can be found in Section S2.7.

### 2.8 Density Functional Theory Calculations

All DFT calculations were carried out using the CASTEP version 22.11 with the program’s standard default on-the-fly pseudopotentials. The exchange–correlation energy functional was approximated within the generalized-gradient approximation (GGA) framework using the Perdew–Burke–Ernzerhof functional for solids (PBESol) parametrization. For simplification, scalar relativistic potentials without spin–orbit coupling were used for all geometry optimizations and electronic structure calculations. We used a plane wave cutoff of 32 Ry for the wave functions and a Γ-centered Monkhorst–Pack (MP) 6 × 6 × 4 k-point grid for the Brillouin zone (BZ) sampling in all calculations. For more details including treatment of the A-cation site disorder, please see Section S2.8.

## 3 Results and Discussion

Organic–inorganic hybrid halides formed with the azetidinium cation (C_3_H_8_N^+^) and pnictogen metal (Sb^3+^ and Bi^3+^) cations were systematically investigated by both experimental and calculation methods (see Section S1 for full details). All combinations (except B = Bi and X = Cl) form structures based on the 6H perovskite polytype framework, differing only in the site(s) of ordered B-site vacancies. Across all combinations, only two basic perovskite structures were found to be adopted, both derived from the 6H framework shown in Figure 1a.

**A.** For perovskites with large pnictogen cations (B = Sb^3+^ and Bi^3+^), it is rare to observe face-sharing octahedra (FSO); instead, corner-sharing octahedra (CSO) are more common in order to avoid close B–B distances. In B-site-deficient perovskites, vacancies are therefore preferentially located at FSO sites. The most common 6H variant for A_3_B_2_Cl_9_ is a 1D (hcc)_2_ polytype with alternating occupancy of vacancies at FSO sites to form a zigzag chain, as shown in Figure 1d (e.g., Cs_3_Bi_2_Cl_9_ and (MA)_3_Bi_2_Cl_9_).^45^ In surprising contrast to this expectation, Az_3_Sb_2_Cl_9_ and Az_3_Sb_2_Br_9_ adopt a 6H polytype framework but with the B-site vacancies instead occupying one layer of face-sharing octahedral (FSO) sites, which reduces the dimensions of the inorganic framework to 2D, as shown in Figure 1b. This 2D (hcc)_2_ A_3_B_2_X_9_ perovskite polytype was previously only hypothesized in the structural screening of all potential 6H A_3_B_2_X_9_ perovskites until the recent successful preparation of Az_3_Sb_2_Cl_9_ and Az_3_Sb_2_Br_9_ by Luo et al.^33^ and independently in this work,^46^ and of Az_3_Bi_2_Cl_9_ by Jin et al.^30^

**B.** 0D (hcc)_2_ A_3_B_2_X_9_ perovskite polytypes were found in Az_3_Sb_2_I_9_, Az_3_Bi_2_Br_9_, and Az_3_Bi_2_I_9_, with the latter two in agreement with Jin et al.^30^ In this 0D 6H polytype, B-site vacancies preferentially occupy the corner-sharing octahedral (CSO) sites, leading to a connectivity reduction from 3D to 0D, as shown in Figure 1c. This polytype is a widely observed crystal structure associated with relatively smaller B-site cations (e.g., Cs_3_Cr_2_Cl_9_) and/or larger anions (Cs_3_Bi_2_I_9_).^47^ As this structure consists of pairs of face-sharing octahedra, it is also termed a dimer structure in some reports. However, it is worth noting that a dimer structure can also be observed in 0D 2H (h)_2_ polytypes (e.g., Cs_3_Tl_2_Cl_9_ and Cs_3_Cr_2_I_9_), consisting of simple hexagonal close-packed AX_3_ sequences.^48^

In addition to the close-packed Az_3_B_2_X_9_ perovskite polytype structures described above, two nonperovskite compounds with a 2:1:5 stoichiometry, general formula A_2_BX_5_, were also observed as strongly competing side products in both the bismuth chloride and bromide systems. Both Az_2_BiCl_5_ and Az_2_BiBr_5_ adopt a 1D chain structure with corner-sharing [BiX_6_]^3–^ units as previously reported,^49,50^ and so are not discussed in any detail.

### 3.1 2D (hcc)_2_ Perovskite Structure for Az_3_Sb_2_Cl_9_ and Az_3_Sb_2_Br_9_

The crystal structures of Az_3_Sb_2_Cl_9_ and Az_3_Sb_2_Br_9_ were determined by single-crystal X-ray diffraction (SCXRD) at 293, 173, and 100 K. As described above, both adopt a 2D (hcc)_2_ polytype, which results from vacancy ordering in a single FSO layer, as previously demonstrated^33^ and shown in Figure 1a. This structure is rarely observed in either inorganic pnictogen perovskites or organic–inorganic pnictogen hybrids. To form such a structure, the inversion center must be broken to allow each face-sharing octahedral crystallographic site to be solely occupied by either vacancies or metal cations, as demonstrated in Figure 1b. Unlike other polar (c)_3_ cubic layered perovskites or other A_3_B_2_X_9_ polar hexagonal perovskites with (hhc)_3_ stacking modes, the loss of the inversion center and formation of the resulting polar structure are intrinsically related to the arrangement of the B-site vacancies within the 6H framework, rather than to the specific ordering of organic molecules or octahedral distortion induced by temperature lowering.^34,51,52^ Hence, this unique octahedral network provides an intrinsic polar axis perpendicular to the close-packed layers, and the aristotype structure has *P*6_3_ *mc* symmetry. In addition to Az_3_Sb_2_Cl_9_ and Az_3_Sb_2_Br_9_, a similar nonpolar-to-polar transition induced by B-site vacancies was also observed in the hexagonal close-packed 2H 0D (h)_2_ Cs_3_Ga_2_F_9_ ^23^ and 4H 0D (hc)_2_ K_2_GeF_6_.^53^

At room temperature, single-crystal diffraction data indicate that Az_3_Sb_2_Cl_9_ and Az_3_Sb_2_Br_9_ are both isostructural and isosymmetric, crystallizing in the aristotype space group *P*6_3_ *mc* of the layered structure as described above and in agreement with Luo et al.^33^ (see Table S3.1 for full crystallographic details). The room-temperature structure is shown in Figure 2: the Sb^3+^ is octahedrally coordinated and corner-shares with nearby octahedra to form [Sb_2_X_9_]_∞_ layers extending in the close-packing (*ab*) plane. In comparison with the parent 6H perovskite structure with the *P*6_3_/*mmc* space group, the symmetry breaking at the mirror plane between two symmetry-equivalent FSO B-sites leads to a splitting of Wyckoff positions, as two inequivalent sites are now occupied by B-site cations and vacancies in separate layers. This symmetry breaking also results in an increase of the number of both A-sites and X-sites from two to three. At the three A-sites, the Az^+^ cation shows symmetry-induced disorder due to the symmetry mismatch between Az^+^ and the 6_3_ screw axis. A similar problem of Az disorder and incompatibility with 6_3_ symmetry was encountered by Luo et al.,^33^ who attempted to model this disorder by introducing three different disordered Az orientations, modeled by octahedral, cubic, and triprismatic motifs.

**Figure 2:**
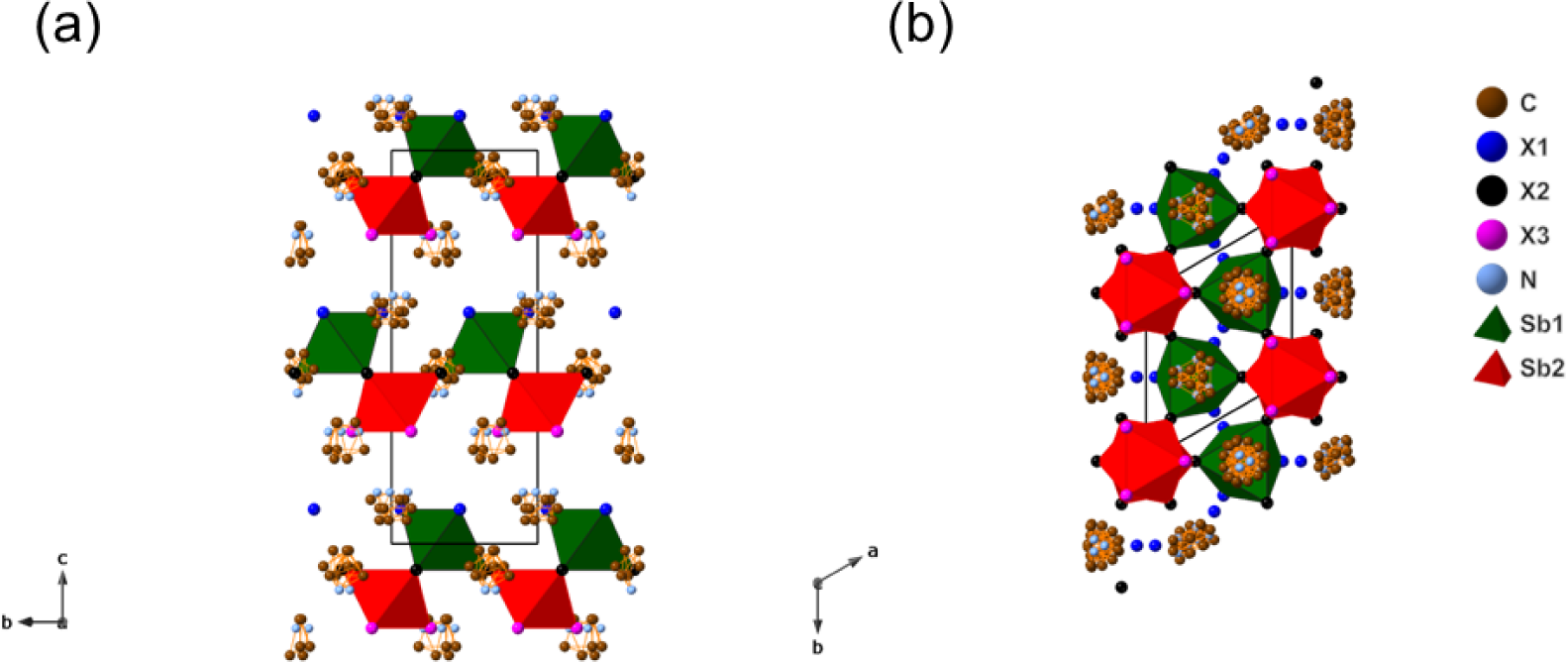
Room-temperature crystal structures of *P*6_3_ *mc* Az_3_Sb_2_Cl_9_ and Az_3_Sb_2_Br_9_ viewed (a) along and (b) perpendicular to the packing layers (the disordered azetidinium cations are drawn without hydrogen). Note the split sites for constituent atoms of the A-site molecule to account for the disorder.

The *P*6_3_ *mc* space group is polar, and so the material must be piezoelectric. As discussed above, the symmetry breaking to a polar space group is solely due to the vacancy ordering, and although pyroelectric behavior would also be a possibility if an additional polar distortion was present, there appears to be no crystallographic evidence of a spontaneous polarization, i.e., cooperative ion displacements or A-cation alignment, at any of the temperatures where data were collected. Room-temperature piezoresponse force microscopy (PFM) was carried out on single-crystal samples of both Az_3_Sb_2_Cl_9_ and Az_3_Sb_2_Br_9_ to confirm the polar nature suggested by SCXRD. In essence, PFM evaluates the piezoelectric response associated with the polar phase through the application of a localized oscillating electrical bias to a nanoscale metallic probe. In both samples, the response was found to be relatively uniform across the sample surfaces; hence, single-pixel data taken over a period of time were compared, and the response of the surface under increasing electrical bias (1 to 5 V) was recorded, as shown in Figure 3. The responses observed in single-crystal samples of both Az_3_Sb_2_Cl_9_ and Az_3_Sb_2_Br_9_ were compared to amorphous glass (nonpiezoelectric, no signal) and a sample of periodically poled lithium niobate (LNO), which displays a strong signal associated with a monodomain region. The average PFM amplitude response of the samples, plotted against different excitation voltages, scales linearly for all samples except glass, as expected. The gradients demonstrate that Az_3_Sb_2_Br_9_ has a higher effective *d*_33_ piezoelectric coefficient (1.8 ± 0.3 pm V^–1^) compared to Az_3_Sb_2_Cl_9_ (0.6 ± 0.3 pm V^–1^), based on comparison to the known *d*_33_ coefficient of z-cut lithium niobate, further confirming that both crystals are polar and piezoelectric. To exclude possible contributions from electrostriction, the piezoelectric signals of Az_3_Sb_2_Cl_9_ and Az_3_Sb_2_Br_9_ at 2ω frequencies were measured, and no response was detected. The amplitude of *d*_33_ in the piezoelectric tensor for both compounds confirms an intrinsic polar direction along the close-packed direction of the *c*-axis, consistent with their *P*6_3_ *mc* space group crystal structure.

**Figure 3:**
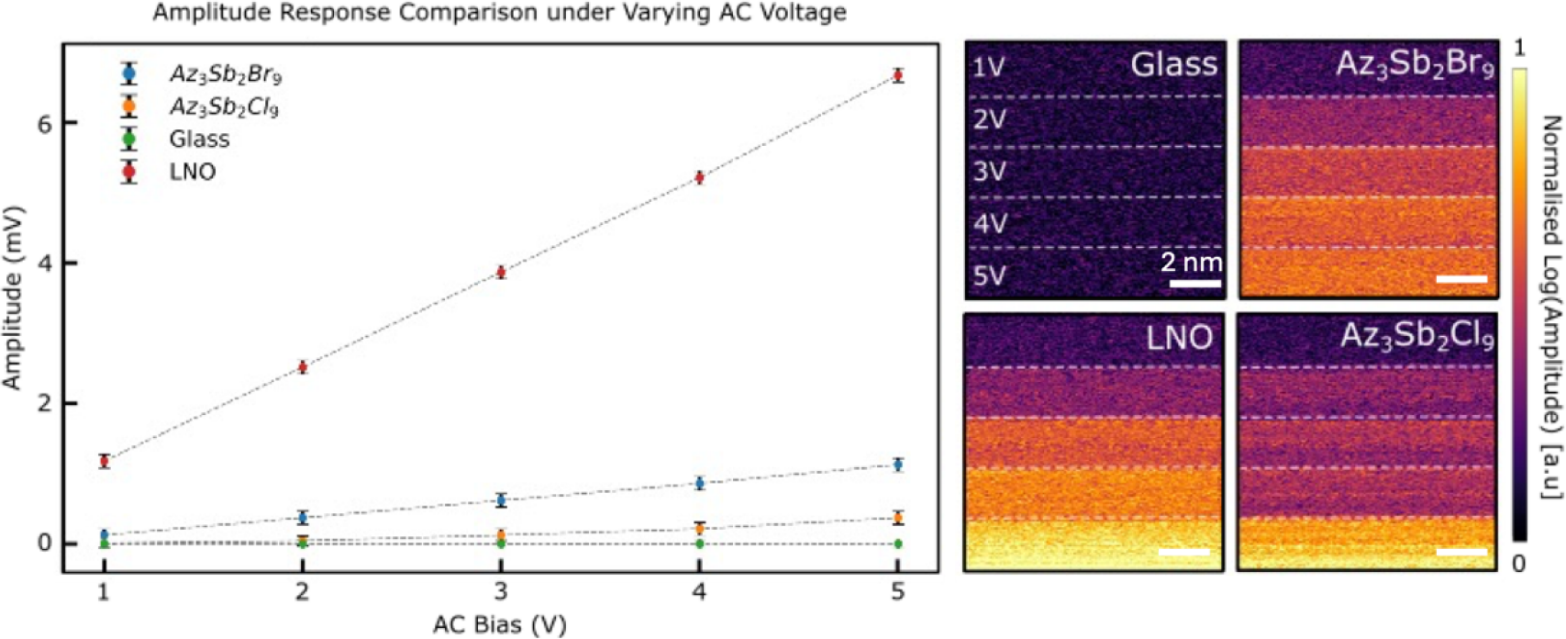
Dependence of the piezoelectric coefficient *d*_33_ on the amplitude of the applied AC field in comparison to transitional piezoelectric LiNbO_3_ (LNO) and nonpiezoelectric glass.

Dielectric measurements were carried out on polycrystalline pellets for both Az_3_Sb_2_Cl_9_ and Az_3_Sb_2_Br_9_ in the temperature range of 30–310 K with a frequency range from 100 Hz to 10 MHz in order to determine any structural changes and, hence, potential changes in piezoelectric performance. The temperature and frequency dependence of the real (ε’) and imaginary (ε’’) parts of the complex dielectric permittivity for Az_3_Sb_2_Cl_9_ and Az_3_Sb_2_Br_9_ are shown in Figure 4. For Az_3_Sb_2_Cl_9_, two dielectric anomalies are observed in the ε’(ω, *T*) response, as shown in Figure 4a. The first is a frequency-independent discontinuity in the ε’(ω, *T*) curve at 220 K, which was also observed by Luo et al.;^33^ such a lambda-type profile is typically associated with a first-order structural phase transition (PT) and was previously assigned as a transition from the RT *P*6_3_ *mc* to *Pna*2_1_.^33^ The second dielectric anomaly observed at lower temperatures in our ε’(ω, *T*) data is a broad frequency-dependent peak observed around 100 K, and this feature is more clearly evident in the ε’’(ω, *T*) curves, as shown in Figure 4b. From both curves, the shift of peaks toward higher temperatures with increasing applied frequencies indicates thermally activated dielectric relaxation behavior.^54,55^ A similar frequency-dependent dielectric response has been observed in both methylammonium and formamidinium lead iodide 3C perovskites and was attributed to a dynamic slowing of the A-site cation motion upon cooling.^56^ When combined with the observed changes in the temperature-dependent diffraction data (discussed in detail below), it is therefore likely that this is also due to subtle changes in the Az^+^ position and dynamics with varying temperature.

**Figure 4:**
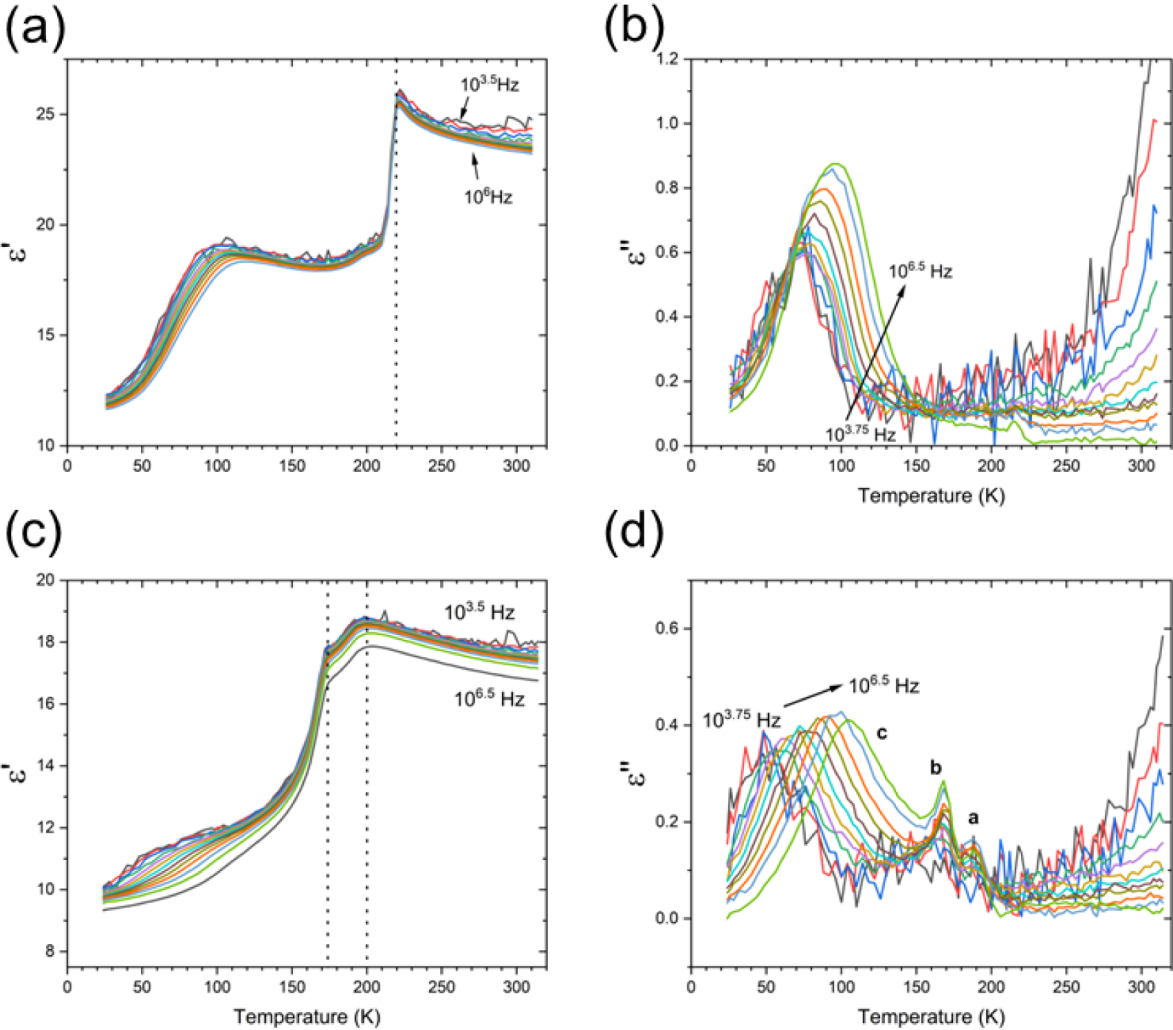
Dielectric data for Az_3_Sb_2_Cl_9_ (top) and Az_3_Sb_2_Br_9_ (bottom) collected upon cooling, showing both real, ε’ (a, c) and imaginary, ε’’ (b, d) parts of the complex dielectric permittivity as a function of frequency (ω) and temperature (*T*).

Az_3_Sb_2_Br_9_ shows a slightly different dielectric response compared to Az_3_Sb_2_Cl_9_, as demonstrated in Figure 4c,d. Two dielectric anomalies are observed in the ε’(ω, *T*) curves at ca. 200 and 173 K, whereas dielectric dissipation peaks are observed in the ε’’(ω, *T*) curves at ca. 188, 170, and 80 K, respectively. The frequency-independent dielectric anomalies in both the ε’(ω, *T*) and ε’’(ω, *T*) curves suggest two PTs at 200 and 173 K, marked as **a** and **b** in Figure 4d. At lower temperatures (<100 K), a similar thermally activated dielectric relaxation peak **c** is also observed in Az_3_Sb_2_Br_9_. It is worth noting that a similar double peak in ε’ is also present in the data of Luo et al. in the region of 170–200 K for Az_3_Sb_2_Br_9_, but the presence or origin of this feature is not acknowledged in that study, and also dielectric data below 150 K was not reported.^33^

Differential scanning calorimetry (DSC) was performed between 300 and 100 K on both compounds to further investigate these successive PTs. The DSC data are shown in Figure 5, which confirm the presence of several thermal events indicative of PTs, in agreement with the dielectric spectra for both compounds. A more detailed analysis of the thermal events is summarized in Table S3.9. The successive phases are labeled I to IV in order of appearance on cooling from room temperature, as shown in Figure 5. From the calculated entropy change, Δ*S*_I→II_ = 18 J K^–1^ mol^–1^ in Az_3_Sb_2_Cl_9_, the PT (I to II) shows a typical order–disorder nature at 213/220 K (cooling/heating), which agrees with the previous report from Luo et al. and is also consistent with the observed dielectric response, as shown in Figure 4a,b.^33^ Three PTs were observed in the DSC data of Az_3_Sb_2_Br_9_ at 202/202, 199/197, and 187/187 K, which also correlate with the observed ε’(ω, *T*) and ε’’(ω, *T*) data, as shown in Figure 4c,d. Although Luo et al. also observed these additional features in their DSC data, they did not report any crystallographic data nor discuss potential origins of these transitions. It is interesting to note that the low-temperature *Pna*2_1_ structure they reported is from the data obtained at 123 K, which would correspond to phase IV based on our labeling and DSC data from both their and our studies. From our DSC data, the amplitude of the calculated entropy changes, Δ*S*_I→II_ = 4, Δ*S*_II→III_ = 3, and Δ*S*_III→IV_ = 10 J K^–1^ mol^–1^, combined with the single-crystal diffraction analysis of the Az^+^ position (see below) and frequency-dependent dielectric response, suggest an order–disorder nature for these PTs.^57–59^ As discussed in more detail in the following section, variable-temperature PXRD of this compound shows a subtle and complex structural change at low temperatures involving a large supercell and possible incommensurate modulation driven by changes in the Az cation dynamics and orientation.

**Figure 5:**
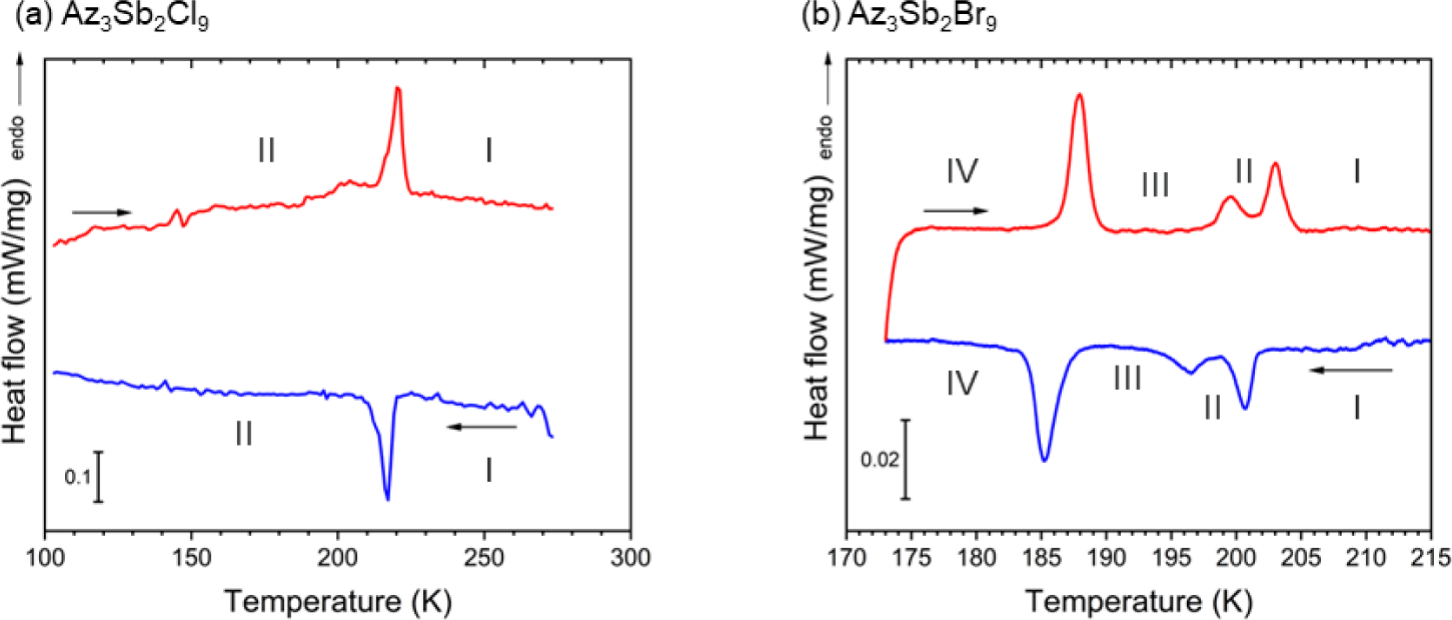
LT-DSC curves for (a) Az_3_Sb_2_Cl_9_ with a heating/cooling rate of 10 K min^–1^ and (b) Az_3_Sb_2_Br_9_ with a heating/cooling rate of 2 K min^–1^, showing successive phase transitions upon cooling (blue) and heating (red).

Both DSC and dielectric data clearly indicate the presence of successive phase transitions below room temperature for both compounds, and variable temperature (VT) XRD was carried out to investigate the structural origin of these transitions. From the VT-XRD data for Az_3_Sb_2_Cl_9_, three phase transitions are evident between 300 and 60 K. The first anomaly observed in DSC data at 220 K can be attributed to an order–disorder PT from the parent disordered hexagonal *P*6_3_ *mc* phase I (as determined by RT SCXRD) to an orthorhombic superstructure phase II, as suggested by both LT SCXRD and PXRD data. The crystal structure, as initially determined by SCXRD data measured at 173 K, is assigned to the orthorhombic space group *Pna*2_1_ with a unit cell expansion from the parent hexagonal cell: *a*_II_ ≈ √3*a*_0_, *b*_II_ ≈ *a*_0_, and *c*_II_ ≈ *c*_0_, again in agreement with Luo et al.^33^ This first PT in Az_3_Sb_2_Cl_9_ involves the ordering of two A-site organic cations, a decrease in the degree of disorder in the third, and octahedral tilting of [SbCl_6_]^3–^. To understand the configurational ordering of the organic Az^+^ cation, it is helpful to analyze the calculated Δ*S* in terms of Boltzmann statistics. Based on the crystallographic mechanism of this PT, the calculated Δ*S*_I→II_ = 18 J K^–1^ mol^–1^ is close to the theoretical value of 3*R*ln2 = 17 J K^–1^ mol^–1^ and further supports the ordering of the molecular A-site cations at two nonequivalent sites in Az_3_Sb_2_Cl_9_.^34,60^ In addition, the tilting of the octahedral network can be described by a linear combination of three distortion modes with irreducible representations: Γ_1_^+^, Γ_5_^+^, and M_2_^+^ using the ISODISTORT package of the ISOTROPY software suite.^61,62^ The Γ_1_^+^ mode only contributes to the general shrinkage of the unit cell and shows no association with any octahedral distortion. The Γ_5_^+^ mode mainly involves an in-plane movement of bridging anions (linking CSO) along the [100] direction, whereas the M_2_^+^ mode dominates the in-phase tilting distortion of both Sb1 and Sb2 octahedra, as shown in Figure 6. The observation of superstructure peaks and orthorhombic distortion in the VT-PXRD data measured between 220 and 210 K also supports this structural evolution, despite the strong preferred orientation (PO) along (00L), as demonstrated in Figure 7.

**Figure 6:**
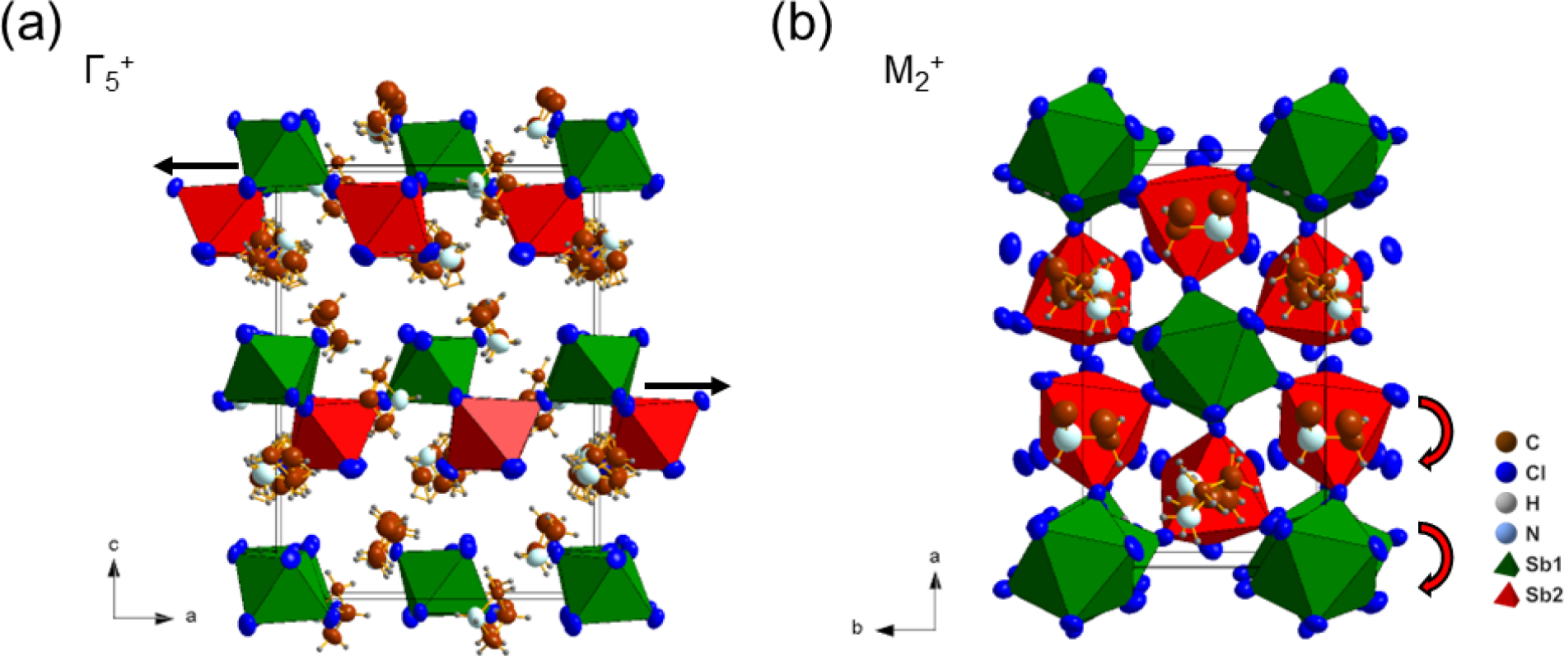
Structural evolution of the *Pna*2_1_ phase II in Az_3_Sb_2_Cl_9_ from the parent phase I *P*6_3_ *mc*, highlighting the distortions associated with the (a) Γ_5_^+^ and (b) M_2_^+^ modes.

**Figure 7:**
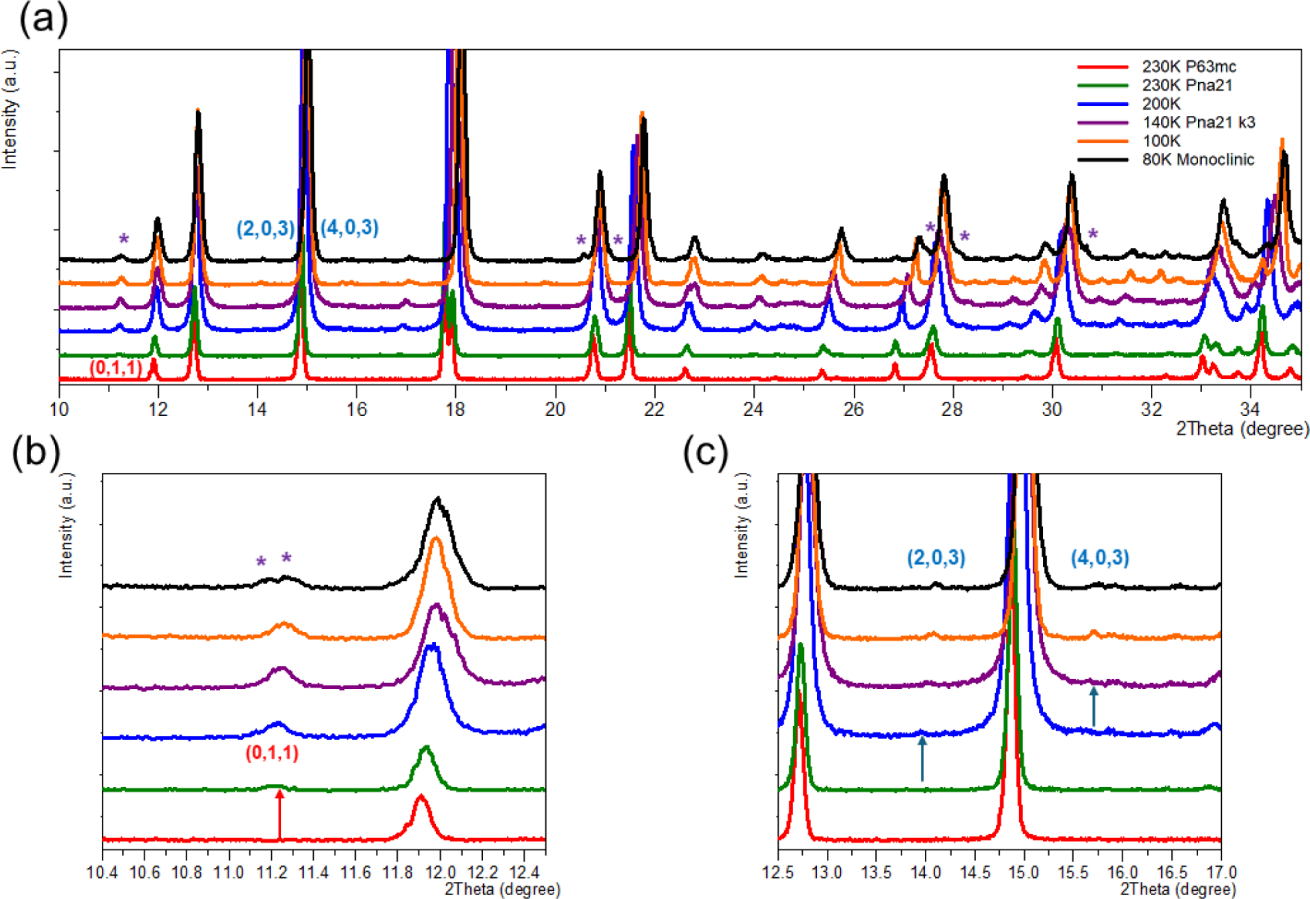
(a) LT-PXRD profile of Az_3_Sb_2_Cl_9_; zoomed sections highlighting (b) the growth and splitting of (011) during the PTs (I→II) and (II→III), respectively, and (c) superstructure peaks (203) and (403) during PT (II→III) indicating a monoclinic distortion at ca. 90 K, where the asterisks at 80 K represent the peak split during the PT­(II→III). Arrows and asterisks highlight the superlattice peaks.

Below 220 K, a continuous phase transition from phase II *Pna*2_1_ to a larger superstructure phase III can be gradually observed in the VT-PXRD data for Az_3_Sb_2_Cl_9_, characterized by the increasing relative intensities of weak superstructure peaks with decreasing temperature, as indicated in Figure 7. Despite the complexities caused by strong preferred orientation (PO) and uncertainty in the position of the organic cations, the subtle distortion can be attributed to a further loss of translational symmetries, leading to a further tripling of the phase II orthorhombic cell along the *a*-axis, resulting in a supercell with dimensions ca. 44.01 × 8.54 × 19.77 Å, i.e., *a*_III_ = 3*a*_II_ = 3√3*a*_0_, *b*_III_ = *a*_0_, and *c*_III_ = *c*_0_ with respect to the parent (room temperature) hexagonal cell. As no thermal hysteresis is evident in the DSC for PT II–III, the most reasonable solution for the crystal structure is the orthorhombic subgroup *Pna*2_1_ ***k*** 3. At 70 K, further splitting and broadening of specific hk0 peaks suggest a monoclinic distortion of the *Pna*2_1_ orthorhombic supercell. However, successful and unambiguous determination of the space group and unit cell parameters of such a large and complex structure was not possible using Rietveld refinement of PXRD data.

In comparison with Az_3_Sb_2_Cl_9_, the structural evolution of Az_3_Sb_2_Br_9_ at low temperature is more ambiguous, and no satisfactory structural solution can be obtained from SCXRD below the initial phase transition. Although the DSC data indicate two PTs at 202/202 and 198/197 K, no evidence of peak splitting or broadening can be observed in the PXRD data in this range, and hence all data can be well-refined by the phase I *P*6_3_ *mc* structure. At 184 and 180 K, the emergence of superstructure peaks and peak broadening suggest an orthorhombic distortion (see Figure S3.10), but no solution can be found among commensurate candidates. Considering the large shift of low-angle superstructure peaks over a 4 K temperature increment, these data may involve more than one intermediate phase, which complicates the determination of the structure. At 170 K, the appearance of new superstructure peaks and corresponding peak broadening suggests the occurrence of further different PTs from those at 184 and 180 K. Two possible solutions with space groups *Pmn*2_1_ and *Pna*2_1_ can be found for phase IV with a unit cell relation to the parent structure: *a*_IV_ = 2*a*_0_, *b*_IV_ = 3√3*a*_0_, *c*_IV_ = *c*_0_ and *a*_IV_ = 3√3*a*_0_, *b*_IV_ = 2*a*_0_, *c*_IV_ = *c*_0_, respectively, leading to the formation of an orthorhombic 2*a*_0_ × 3√3*a*_0_ × *c*_0_ supercell. Due to the complexities arising from strong PO and uncertain motifs of the organic cations, it is practically impossible to further determine the active modes or atomic displacements from PXRD data via refining the relative intensities. Between 60 and 170 K, no PTs can be detected from PXRD data. The relationship between reduced lattice parameters and temperature for Az_3_Sb_2_Cl_9_ and Az_3_Sb_2_Br_9_ is shown in Figure 8. Given the difficulties in absolute structure solution and exact determination of the Az cation position/orientation, it is likely that these structural changes involve changes in Az^+^ cation disorder, and the dynamics of this are reflected in the frequency-dependent dielectric data observed at low temperature for both compounds, as shown in Figure 4.

**Figure 8:**
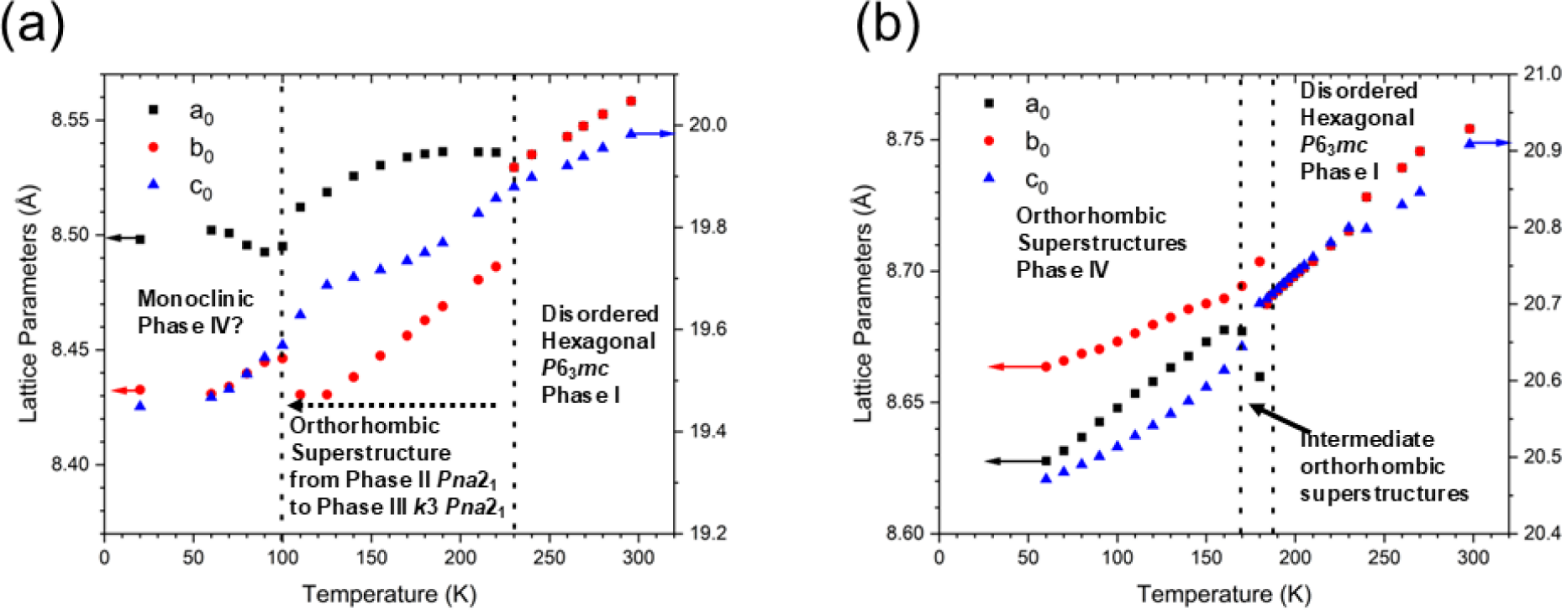
Thermal evolution of the reduced lattice parameters (scaled to the aristotype structure with parameters *a*_0_ = *b*_0_ and *c*_0_) estimated from VT-PXRD by Rietveld refinement in (a) Az_3_Sb_2_Cl_9_; in phase II, *a* = √3*a*_0_, *b* = *b*_0_, *c* = *c*_0_ and in phases III and IV, *a* = 3√3*a*_0_, *b* = *b*_0_, *c* = *c*_0_; and (b) Az_3_Sb_2_Br_9_, in phase I, the reduced lattice parameters *a* = *a*_0_ = *b*_0_, *c* = *c*_0_; in the intermediate phase and phase IV, refined using the space group *Pna*2_1_, *a* = 3√3*a*_0_, *b* = *b*_0_, *c* = *c*_0_.^63^

#### 3.1.1 Solid Solution Az_3_Sb_2_Cl_9–*x*_Br*_x_*

As Az_3_Sb_2_Cl_9_ and Az_3_Sb_2_Br_9_ are isostructural at RT, it is obvious to investigate the tunability of this novel 2D 6H structure via the preparation of the solid solution Az_3_Sb_2_Cl_9–*x*_Br_*x*_. Powder samples of the solid solution series were prepared via conventional solution methods (see the Supporting Information for details). The relative ratios of incorporated chlorine and bromine were estimated by energy-dispersive X-ray spectroscopy. Powder XRD was performed to verify phase purity and study the structural evolution in Az_3_Sb_2_Cl_9–*x*_Br_*x*_ via Rietveld refinement. Further information on EDS analysis and PXRD refinement is detailed in Section S3, Figures 3.11–3.13, and is listed in Tables S3.7 and S3.8.

From the PXRD data and EDS results, it is evident that Az_3_Sb_2_Cl_9_ can form a complete solid solution with Az_3_Sb_2_Br_9_. PXRD indicates that all samples form single-phase compounds in the *P*6_3_ *mc* space group, with the exception of the Az_3_Sb_2_Cl_6_Br_3_ sample, which clearly consists of two phases with distinctive halogen compositions, as shown in Figure 9a. The relationship between the target Br concentration (i.e., precursor solution composition during preparation) and the observed Br content in the resultant powders, as determined from EDS, is shown in Figure 9b. The data clearly suggest a strong preference for samples to be bromine-rich, indicating a higher stability of Az_3_Sb_2_Br_9_ compared to that of Az_3_Sb_2_Cl_9_.

**Figure 9:**
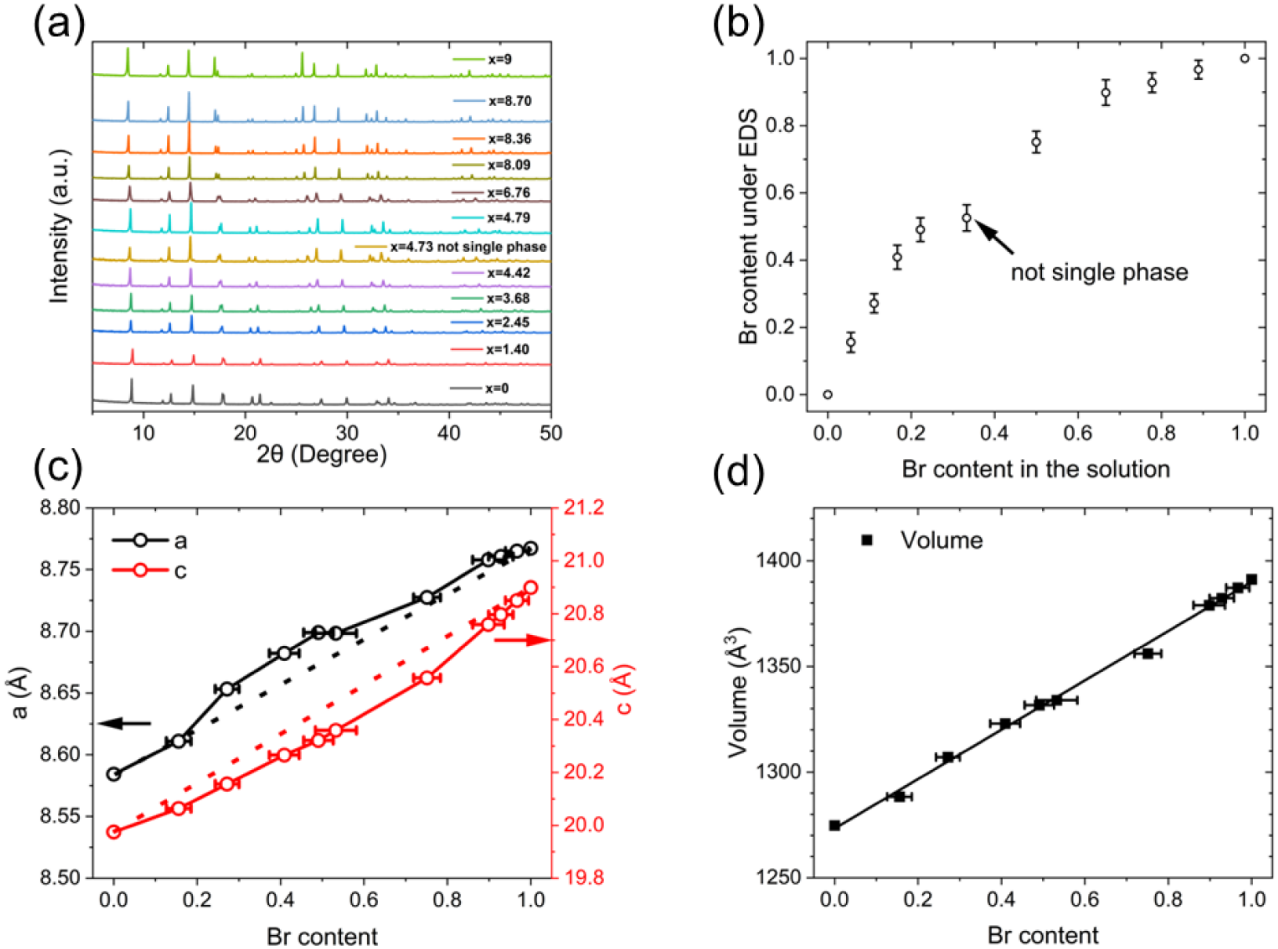
(a) Room-temperature PXRD patterns for Az_3_Sb_2_Cl_9–*x*_Br*_x_*, where the bromine content, *x*, was determined by EDS; (b) relationship between target and measured Br content; dependence of (c) lattice parameters and (d) unit cell volume on proportion of Br substitution (excluding the two phase’Az_3_Sb_2_Cl_6_Br_3_’ sample), where the dotted lines represent the ideal linear relationship of Vegard’s law.

The dependence of lattice parameters and volume on halide composition is shown in Figure 9c (excluding Az_3_Sb_2_Cl_6_Br_3_). From the linear extrapolation between Az_3_Sb_2_Cl_9_ and Az_3_Sb_2_Br_9_ end members, it is clear that the variation of lattice parameters *a* and *c* have positive and negative deviations from Vegard’s law, respectively; however, these effects cancel out, and the volume follows the expected linear dependence. This result suggests that such deviation is not attributed to the overestimation of heavy atom concentration commonly observed in EDS analysis but is caused by the preferential arrangement of bromine anions rather than a homogeneous (random) distribution across the three anion sites. As the room-temperature crystal structure shows (Figure 2), the three inequivalent anion sites are terminal X1, bridging X2, and terminal X3, where the terminal X1 and X3 sites almost share the same chemical environments. Preferential site occupancy by Br was analyzed through Rietveld refinements of PXRD data and showed a weak preference for Br occupancy at the bridging X2 site rather than an even distribution across all sites (see Section S3, pages S18 and S19 for details of methodology). This preferential occupancy is possibly related to the previously observed higher thermodynamic stability of Az_3_Sb_2_Br_9_, as the larger, more polarizable Br anions play a more effective role in reducing the Sb^3+^–Sb^3+^ repulsion in the [Sb_2_X_9_]_∞_^3–^ layers. This weak anion site preference for Br at the bridging anion site in these 2D (hcc)_2_ analogues is in contrast to previous observations in cubic close-packed 2D Cs_3_Sb_2_Cl_9–*x*_Br*_x_* and Cs_4_CdBi_2_Cl_12–*x*_Br*_x_* with an exclusive preferential occupancy of Br at terminal anion sites.^64,65^

The electronic structure and optical band gap of Az_3_Sb_2_Cl_9–*x*_Br*_x_* were investigated by both UV–vis absorbance spectroscopy and DFT calculations. Optical absorption spectroscopy was performed on all Az_3_Sb_2_Cl_9–*x*_Br*_x_* compositions, except for the two-phase Az_3_Sb_2_Cl_6_Br_3_ sample. From the absorbance spectra of Az_3_Sb_2_Cl_9–*x*_Br*_x_*, a red shift in the onset of absorption from ca. 350 nm in Az_3_Sb_2_Cl_9_ to 440 nm in Az_3_Sb_2_Br_9_ can be gradually observed with increasing Br occupancy in the solid solution, as shown in Figure 10a. Due to the disordered nature of the Az cation in the structure and given that the band gap should predominantly be determined by the B–X orbital interactions, our DFT calculations use an inorganic Cs^+^ ion as a proxy for Az^+^ in order to obviate this issue (see Section S2.8 for full methodology). These first-approximation calculations suggest an indirect band gap, as shown in Figures S3.18 and S3.19. Although this is in contrast to the direct band gap determined from the DFT calculations of Lou et al., their methodology to deal with the acknowledged Az disorder, which they modeled using three different site disordering models, is not clear.^33^ The indirect band gap was determined by the Kubelka–Munk transformation and shows the expected systematic decrease with increasing Br content, as shown in Figure 10b.^66^

**Figure 10:**
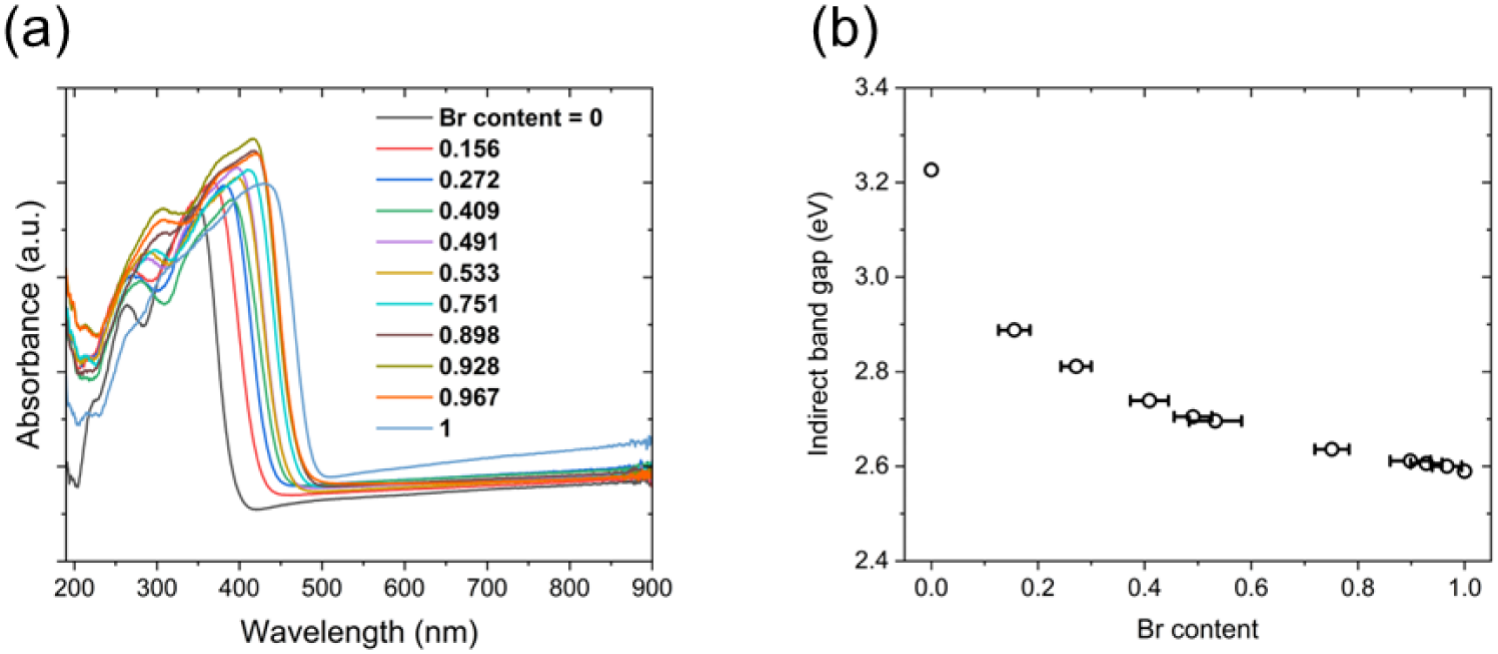
Dependence of (a) optical absorbance and (b) indirect band gap on the proportion of Br substitution in Az_3_Sb_2_Cl_9–*x*_Br*_x_,* where composition was determined by EDS.

### 3.2 0D (hcc)_2_ Perovskite Structure in Az_3_Sb_2_I_9_, Az_3_Bi_2_Br_9_, and Az_3_Bi_2_I_9_

The crystal structures of Az_3_Sb_2_I_9_, Az_3_Bi_2_Br_9_, and Az_3_Bi_2_I_9_ were determined by SCXRD, and the results are summarized in Tables S3.1 and S3.2. Rietveld refinements were performed on the RT PXRD patterns to confirm the space group and lattice parameters determined from SCXRD (refinement details can also be found in Section S3). HT-DTA, LT-DSC, and dielectric measurements were carried out to study the thermal stability and PTs observed in SCXRD.

Az_3_Sb_2_I_9_, Az_3_Bi_2_Br_9_, and Az_3_Bi_2_I_9_ all adopt the 6H 0D (hcc)_2_ polytype shown in Figure 1c, which is frequently observed in other inorganic or organic–inorganic hybrid iodoantimonates­(III) and iodobismuthates­(III),^67^ as well as previously reported for Az_3_Bi_2_Br_9_ and Az_3_Bi_2_I_9_.^30^ In this structure type, the B-site vacancies are exclusively located in the corner-sharing octahedra (CSO) of the 6H type, resulting in a structural framework consisting of face-sharing [B_2_X_9_]^3–^ octahedral dimer units, yielding a discrete B-site polyhedra motif with zero-dimensional connectivity (^0^[*B*_2_ *X*_9_]). At high temperature, these inorganic and specific hybrids adopt the prototypic 6H 0D structure with a *P*6_3_/*mmc* space group. In this hexagonal phase, the organic cation tends to be highly disordered, with large atomic displacement ellipsoids and symmetry-induced disorder of atomic positions. With decreasing temperature, the rotations of the organic cations and the distortion of [B_2_X_9_]^3–^ units typically lead to symmetry-breaking PTs, from the aristotype 6/*mmm* symmetry to *mmm* in GU^+^-based hybrids or to 2/m in TMS^+^-based hybrids, as shown in Table 1.^34,55^ However, it is likely that some disorder of the organic cation may endure even in the low-temperature phases of many hybrid perovskites.^34,55^

At room temperature, single-crystal data indicate Az_3_Sb_2_I_9_, Az_3_Bi_2_Br_9_, and Az_3_Bi_2_I_9_ are isostructural, all crystallizing in the *Cmcm* space group, as shown in Figure 11. This disagrees with the *Cmc*2_1_ and *P*6_3_ *mc* symmetries reported by Jin et al., for Az_3_Bi_2_Br_9_ and Az_3_Bi_2_I_9_, respectively, based on powder XRD analysis.^30^ It is worth noting that *Cmc*2_1_ and *P*6_3_ *mc* are polar subgroups of *Cmcm* and the aristotype *P*6_3_/*mmc* symmetries, respectively. The authors provide no detailed discussion or physical property measurements to support the choice of polar space groups over the nonpolar equivalents; however, they did acknowledge the difficulty in dealing with the Az disorder based on powder data analysis and also had a significant amount of impurity phase present in the case of the iodide sample, which may have affected the accuracy of their analysis. For each of the compounds prepared in this study, there is no evidence of any high-temperature phase transition to the aristotype *P*6_3_ */mmc* space group in DTA before reaching the decomposition point at ∼450 K (see Figures S3.15 and S3.16 for details), nor of any properties suggestive of polar symmetry. In the octahedral dimers, the metal cation occupies the 8f Wyckoff crystallographic site with nearly identical longer bonds to X anions at the bridging (h-layer) sites and also nearly identical shorter bonds to the terminating X sites in the c-layer. Such a bond length distribution is widely observed in most bioctahedral A_3_B_2_X_9_ structures and is generally associated with B^3+^–B^3+^ repulsion. The three organic Az^+^ cations per formula unit occupy two symmetry-inequivalent sites and, based on SCXRD analysis, are disordered, with both imperfect positioning over symmetry elements and additional positional disorder.

**Figure 11:**
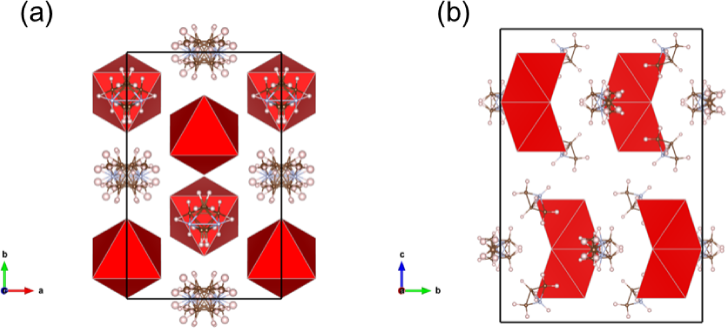
Crystal structure of the room-temperature *Cmcm* phase of Az_3_B_2_I_9_ with the [B_2_X_9_]^3–^ dimer structure viewed perpendicular (a) and parallel (b) to the close-packing planes.

From DSC data, Az_3_Sb_2_I_9_, Az_3_Bi_2_Br_9_, and Az_3_Bi_2_I_9_ demonstrate several PTs during cooling (Figure 12). From the calculated thermodynamic values, Az_3_Sb_2_I_9_ exhibits one order–disorder phase transition at 289/278 K (heating/cooling). In the bismuth analogues, two phase transitions were observed between 298 and 100 K; the calculated entropies of the first PT (I→II) in both compounds at 294/294 K for bromide (Δ*S*_I→II_ = 27 J K^–1^ mol^–1^) and 238/229 K for iodide (Δ*S*_I→II_ = 16 J K^–1^ mol^–1^) suggest an order–disorder transition. The second peak for the II→III PT in both compounds, at 195.4 K for bromide and 172.8 K for iodide, is more readily observed in the heating cycle and is less discernible during cooling.

**Figure 12:**
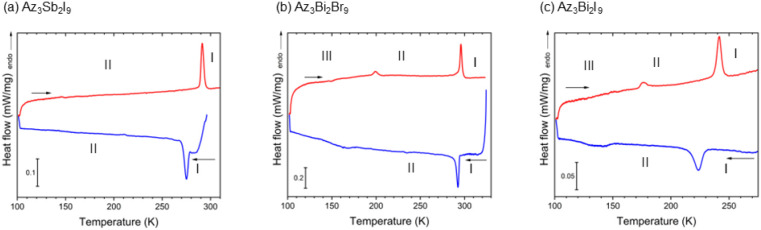
LT-DSC curves for (a) Az_3_Sb_2_I_9_, (b) Az_3_Bi_2_Br_9_, and (c) Az_3_Bi_2_I_9_, collected upon both cooling (blue) and heating (red) with a cooling/heating rate of 10 K min^–1^. Note the endothermic-like feature in the initial stages of the cooling trace in (a) and (b) is an artifact associated with the data collection methodology.

Upon cooling from room temperature, Az_3_Sb_2_I_9_, Az_3_Bi_2_Br_9_, and Az_3_Bi_2_I_9_ all demonstrate similar order–disorder PTs from the *Cmcm* orthorhombic structure (I) to a *Pbcm* superstructure (II) (the two Bi compounds, Az_3_Bi_2_Br_9_ and Az_3_Bi_2_I_9_, both undergo a further PT (II→III) to a *Pnma* structure, described in detail later). In all three compounds, the I→II phase transition comprises ordering of the organic cation and tilting of inorganic [B_2_X_9_]^3–^ dimers. The observed Δ*S* in Az_3_Sb_2_I_9_ and Az_3_Bi_2_I_9_ are 17 J K^–1^ mol^–1^ and 16 J K^–1^ mol^–1^, which is close to theoretical Δ*S*_theo_ = 3*R*ln2 = 17 J K^–1^ mol^–1^. However, the observed Δ*S* in Az_3_Bi_2_Br_9_ is 27 J K^–1^ mol^–1^, which agrees with the theoretical value Δ*S* = 3*R*ln3 = 27 J K^–1^ mol^–1^. Assuming that in the ordered phase II, the three Az^+^ are static without any orientational or positional disorder, the corresponding entropy change should be equal to 3*R*ln3, as for the reported temperature-induced PTs in (PY)_3_Sb_2_Cl_9_.^34^ This value is very close to the experimental results for Az_3_Bi_2_Br_9_. The smaller entropy change for both Az_3_Sb_2_I_9_ and Az_3_Bi_2_I_9_ suggests an already partially ordered orientation of Az^+^ in the high-temperature phase (I) rather than complete disorder. In the PTs (II→III) of the bismuth compounds, the crystal structure transforms from *Pbcm* II into *Pnma* III and this involves a change in the octahedral tilt, as described below.

The tilting of inorganic dimers in the I to II PT can be described by a linear combination of distortion modes with irreducible representations, as determined using ISODISTORT. Two distortion modes Γ_1_^+^ and Υ_3_^–^ are active in the *Pbcm* phase (II) relative to the parent *Cmcm* phase (I); the influence of these modes on the crystal structure is depicted in Figure 13. The Γ_1_^+^ modes in all phases consist of general strain modes of the unit cell and an in-phase tilting distortion of the BX_6_ octahedra about the [100] direction, while the Υ_3_^–^ modes consist of an in-phase rotation of the BX_6_ octahedra about the [001] direction. Despite the similarity of the influence of the Γ_1_^+^ modes on octahedral distortion, Γ_1_^+^ leads to a minor compression of the unit cell along the *b* and *c* axes in Az_3_Bi_2_Br_9_. However, in both iodides, Γ_1_^+^ generates a large expansion of the unit cell along the *b* axis but with considerable shrinkage of the unit cell along the *a* and *c* axes with decreasing temperature. Corresponding to this difference, it can be observed from the polyhedral distortion parameters that in the iodides, the B–I bond length and BI_6_ polyhedral volume decrease after the phase transition, while in the bromide, the Bi–Br bond length and BiBr_6_ polyhedron exhibit an expansion after the phase transition. This is possibly related to the ordering of the organic cation. In the PT from II to III in the bismuth analogues, the dominant distortion mode Υ_3_^–^ in phase II is replaced by Υ_2_^+^, consisting of a sudden change of the in-phase rotation direction in the nearby [B_2_X_9_]^3–^ dimers about the close-packed direction, as demonstrated in Figure 14.

**Figure 13:**
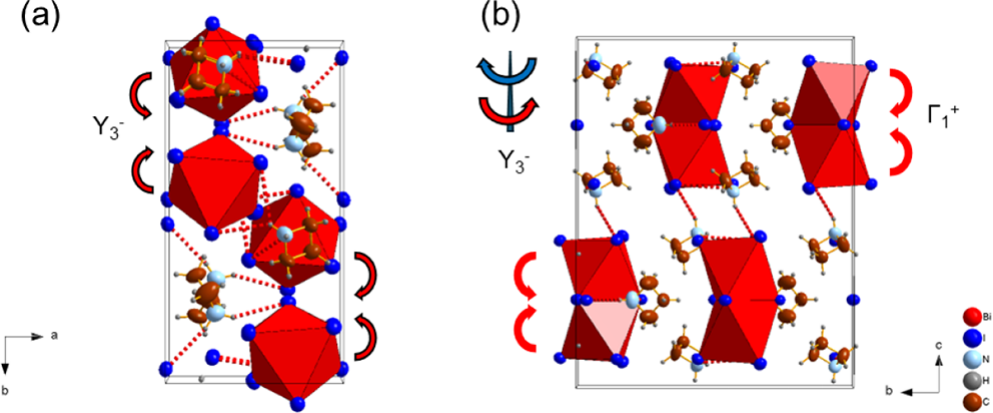
Crystal structure of Az_3_Bi_2_I_9_, as determined by SCXRD at 173 K, showing the evolution of the *Pbcm* phase II, with the [B_2_X_9_]^3–^ dimer structure highlighting the distortions associated with the (a) Y_5_^+^ and (b) Γ _1_^+^ modes in comparison with the parent *Cmcm* phase I. This is also observed in Az_3_Sb_2_I_9_ and Az_3_Bi_2_Br_9_ (the weak N–H···I hydrogen bonds are emphasized by red dotted lines).

**Figure 14:**
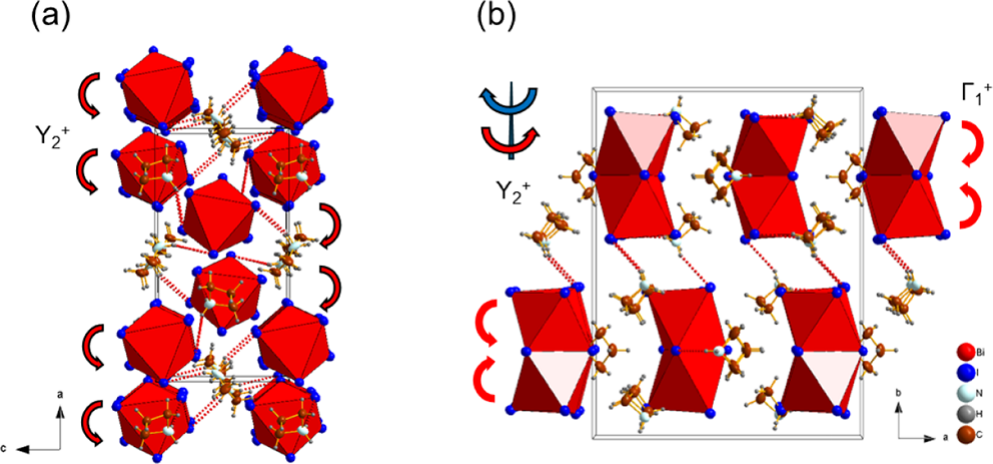
Crystal structure of Az_3_Bi_2_I_9_, as determined by SCXRD at 100 K, showing evolution of *Pnma* phase III and highlighting the distortions associated with the (a) Y_2_^+^ and (b) Γ _1_^+^ modes in comparison to parent *Cmcm* phase I (the weak N–H···I hydrogen bonds are emphasized by red dotted lines).

The influence of the PTs on the dimer structures was also studied by dielectric measurements for Az_3_Sb_2_I_9_, Az_3_Bi_2_Br_9_, and Az_3_Bi_2_I_9_. The dielectric data measured between 100 and 310 K show no evidence of any anomalies associated with the successive PTs observed in the DSC and structural data, suggesting that these small distortions have little effect on the charge displacement process in these particular compounds. The electronic structures of Az_3_Sb_2_I_9_, Az_3_Bi_2_I_9_, and Az_3_Bi_2_Br_9_ were studied by UV–vis spectroscopy and ab initio calculations by DFT (see Supporting Information for full details), which indicate an indirect band gap for all three compounds. From a Kubelka–Munk transformation of the absorbance spectra, band gaps of 1.99, 2.07, and 2.66 eV were determined for Az_3_Bi_2_I_9_, Az_3_Sb_2_I_9_, and Az_3_Bi_2_Br_9_, respectively (Figure S3.17).

It is worth noting that during preparation of both Az_3_Bi_2_Cl_9_ and Az_3_Bi_2_Br_9_, the competing nonperovskite ’2:1:5′ phases Az_2_BiCl_5_ and Az_2_BiBr_5_ were often formed. In the Bi–Br system, the 2:1:5 phase was prevalent when using near-stoichiometric ratios during synthesis; however, high-purity Az_3_Bi_2_Br_9_ can be obtained, for example, by using saturated Bi^3+^ solutions as described in Section S1. These “2:1:5” 1D phases typically consist of either *trans*- or *cis*-connected chains of BX octahedra. Both Az_2_BiCl_5_ and Az_2_BiBr_5_ have been previously reported in detail; both form a *cis*-type arrangement and undergo a reversible paraelectric (*Pnma*)–ferroelectric (*P*2_1_) phase transition at around 230 K.^49,50^ During the current study, these competing impurity phases were also synthesized and analyzed. The results are in full agreement with these prior reports but are included throughout the Supporting Information for reference.

## 4 Summary and Conclusions

A range of azetidinium pnictogen (B = Sb^3+^ and Bi^3+^) halides (X = Cl^–^, Br^–^ and I^–^) were systematically synthesized and studied. Two different perovskite polytype structures were derived from the hexagonal 6H perovskite structure. Az_3_Sb_2_Cl_9_ and Az_3_Sb_2_Br_9_ show a unique 2D polar (hcc)_2_ perovskite structure at room temperature, arising from the preferred arrangement of B-site vacancies in one layer of face-sharing octahedral sites. Low-temperature DSC and dielectric measurements on both compounds suggest a complex sequence of order–disorder-type PTs takes place in both compounds. These findings were confirmed by low-temperature SCXRD and VT-PXRD, which indicate the formation of superstructures and the final symmetry reduction to a monoclinic superstructure for Az_3_Sb_2_Cl_9_ and to an orthorhombic superstructure for Az_3_Sb_2_Br_9_. The solid solution Az_3_Sb_2_Cl_9–*x*_Br_*x*_ was studied by PXRD and optical absorbance spectroscopy and showed complete substitution and continuous bandgap tunability. A weak Br preferred occupancy at the bridging halogen site was observed, leading to a deviation of lattice parameters and optical band gaps from linear relationships.

In contrast, Az_3_Sb_2_I_9_, Az_3_Bi_2_Br_9_, and Az_3_Bi_2_I_9_ adopt a 0D (hcc)_2_ perovskite structure at room temperature as a result of different vacancy ordering in the corner-sharing octahedral sites. Again, low-temperature DSC indicates the occurrence of successive PTs, and SCXRD suggests these involve both partial order–disorder of the organic azetidinium cation positions and distortion of [B_2_X_9_]^3–^ octahedral dimers while retaining overall orthorhombic symmetry. For Bi combined with either Cl or Br, competing nonperovskite phases Az_2_BiCl_5_ and Az_2_BiBr_5_ were observed. These have a 1D cis-connected octahedral network at room temperatures as previously reported.^49,50^

In conclusion, the complexity within a “simple” single B-site-deficient A_3_B_2_X_9_ stoichiometry clearly highlights vacancy ordering in B-site-deficient perovskites as an additional variable for composition-structure–property tuning in organic–inorganic halide perovskites. The additional compositional flexibility and various parent stacking polytypes (9R, 12H, etc.) afforded by the hexagonal perovskites therefore open a large area of structural space to be investigated within this class of materials, with great potential for material development.

## Supplementary Material



## Data Availability

The research data underpinning this publication can be accessed at 10.17630/c47693ff-3017-4dc9-a7d8-4385765b9d37.

## Notes

Material synthesis, characterization methods, and data including compositional analysis, single-crystal and powder X-ray diffraction, UV–Vis, TGA–DSC, dielectric data, and DFT calculations (PDF)

## References

[1] Wu Y., Li X., Zeng H. (2021). Lead-Free Halide Double Perovskites: Structure, Luminescence,
and Applications. Small Struct..

[2] Igbari F., Wang Z.-K., Liao L.-S. (2019). Progress of Lead-Free
Halide Double
Perovskites. Adv. Energy Mater..

[3] Han Y., Yue S., Cui B.-B. (2021). Low-Dimensional Metal Halide Perovskite
Crystal Materials:
Structure Strategies and Luminescence Applications. Adv. Sci..

[4] Lehner A. J., Fabini D. H., Evans H. A., Hébert C.-A., Smock S. R., Hu J., Wang H., Zwanziger J. W., Chabinyc M. L., Seshadri R. (2015). Crystal and Electronic
Structures
of Complex Bismuth Iodides A_3_Bi_2_I_9_ (A = K, Rb, Cs) Related to Perovskite: Aiding the Rational Design
of Photovoltaics. Chem. Mater..

[5] Pramod A. K., Raj Subramaniam M., Hevia S. A., Batabyal S. K. (2022). Synthesis of lead-free
Cs_3_Sb_2_Cl_3_Br_6_ halide perovskite
through solution processing method for self-powered photodetector
applications. Mater. Lett..

[6] Vargas B., Torres-Cadena R., Rodríguez-Hernández J., Gembicky M., Xie H., Jiménez-Mier J., Liu Y.-S., Menéndez-Proupin E., Dunbar K. R., Lopez N., Olalde-Velasco P., Solis-Ibarra D. (2018). Optical, Electronic,
and Magnetic Engineering of ⟨111⟩ Layered Halide Perovskites. Chem. Mater..

[7] Zhang Y., Fadaei Tirani F., Pattison P., Schenk-Joss K., Xiao Z., Nazeeruddin M. K., Gao P. (2020). Zero-dimensional hybrid
iodobismuthate derivatives: From structure study to photovoltaic application. Dalton Trans..

[8] Lee J.-W., Tan S., Seok S. I., Yang Y., Park N.-G. (2022). Rethinking the A
cation in halide perovskites. Science.

[9] Stein F., Palm M., Sauthoff G. (2004). Structure
and stability of Laves
phases. Part I. Critical assessment of factors controlling Laves phase
stability. Intermetallics.

[10] Chang J. H., Doert T., Ruck M. (2016). Structural
Variety of Defect Perovskite
Variants M_3_E_2_X_9_ (M = Rb, Tl,E = Bi,
Sb,X = Br, I). Z. Anorg. Allg. Chem..

[11] Arakcheeva A. V., Novikova M. S., Zaitsev A. I., Lubman G. U. (1999). Perovskite-like
modification of Cs_3_Sb_2_I_9_ as a member
of the 0D family A_3_B_2_X_9_. J. Struct. Chem..

[12] Yang X., Fernández–Carrión A. J., Geng X., Kuang X. (2024). B-site deficient
hexagonal perovskites: Structural stability, ionic order-disorder
and electrical properties. Prog. Solid State
Chem..

[13] Vargas B., Coutino-Gonzalez E., Ovalle-Encinia O., Sanchez-Ake C., Solis-Ibarra D. (2020). Efficient
Emission in Halide Layered Double Perovskites:
The Role of Sb^3+^ Substitution in Cs_4_Cd_1‑x_Mn_x_Bi_2_Cl_12_ Phosphors. J. Phys. Chem. Lett..

[14] Vargas B., Torres-Cadena R., Reyes-Castillo D. T., Rodríguez-Hernández J., Gembicky M., Menéndez-Proupin E., Solis-Ibarra D. (2020). Chemical Diversity
in Lead-Free, Layered Double Perovskites: A Combined Experimental
and Computational Approach. Chem. Mater..

[15] Lindquist K. P., Boles M. A., Mack S. A., Neaton J. B., Karunadasa H. I. (2021). Gold-Cage
Perovskites: A Three-Dimensional Au^III^-X Framework Encasing
Isolated MX_6_
^3–^ Octahedra (M^III^ = In, Sb, Bi; X = Cl^–^, Br^–^,
I^–^). J. Am. Chem. Soc..

[16] Liu H., Hafeez H., Cordes D. B., Slawin A. M. Z., Peters G., Lee S. L., Samuel I. D. W., Morrison F. D. (2023). Enhanced Photoluminescence
and Reduced Dimensionality via Vacancy Ordering in a 10H Halide Perovskite. Inorg. Chem..

[17] Jakubas R., Rok M., Mencel K., Bator G., Piecha-Bisiorek A. (2020). Correlation
between crystal structures and polar (ferroelectric) properties of
hybrids of haloantimonates­(iii) and halobismuthates­(iii). Inorg. Chem. Front..

[18] Trolliard G., Ténèze N., Boullay P., Mercurio D. (2004). TEM study of cation-deficient-perovskite
related A_n_B_n–1_O_3n_ compounds:
The twin-shift option. J. Solid State Chem..

[19] Nguyen L. T., Cava R. J. (2021). Hexagonal Perovskites as Quantum
Materials. Chem. Rev..

[20] Tidrow S. C. (2014). Mapping
Comparison of Goldschmidt’s Tolerance Factor with Perovskite
Structural Conditions. Ferroelectrics.

[21] Hoard J. L., Goldstein L. (1935). The Crystal Structure of Cesium Enneachlordithalliate,
Cs_3_Tl_2_Cl_9_. J. Chem. Phys..

[22] Dance J., Mur J., Darriet J., Hagenmuller P., Massa W., Kummer S., Babel D. (1986). Magnetic properties of the dimeric iron (III) fluoride: Cs_3_Fe_2_F_9_. J. Solid State
Chem..

[23] De
Kozak A., Mary Y., Gredin P., Renaudin J., Férey G., Babel D. (1994). The crystal structure of the binuclear
fluorocompound Cs_3_Ga_2_F_9_. Eur. J. Solid State Inorg. Chem..

[24] Liu C., Wang Y., Geng H., Zhu T., Ertekin E., Gosztola D., Yang S., Huang J., Yang B., Han K., Canton S. E., Kong Q., Zheng K., Zhang X. (2019). Asynchronous
Photoexcited Electronic and Structural Relaxation in Lead-Free Perovskites. J. Am. Chem. Soc..

[25] Szklarz P., Zaleski J., Jakubas R., Bator G., Medycki W., Falińska K. (2005). The structure,
phase transition and molecular dynamics
of [C­(NH_2_)_3_]_3_[Sb_2_Br_9_]. J. Phys.: Condens. Matter.

[26] Vargas B., Ramos E., Perez-Gutierrez E., Alonso J. C., Solis-Ibarra D. (2017). A Direct Bandgap
Copper-Antimony Halide Perovskite. J. Am. Chem.
Soc..

[27] Frenzen G., Kummer S., Massa W., Babel D. (1987). Tetragonale Fluorperowskite
AM_0.75_ – _0.25_F_3_ mit Kationendefizit:
K_4_Mn^II^Mn_2_
^III^F_12_ und Ba_2_Cs_2_Cu_3_F_12_. Z. Anorg. Allg. Chem..

[28] Saßmannshausen M., Lutz H. D. (2001). Caesiumchromhalogenide
Cs_3_CrCl_6_, Cs_3_Cr_2_Cl_9_ und Cs_3_CrBr_6_ - Darstellung, Eigenschaften,
Kristallstruktur. Z. Anorg. Allg. Chem..

[29] Kamminga M. E., Stroppa A., Picozzi S., Chislov M., Zvereva I. A., Baas J., Meetsma A., Blake G. R., Palstra T. T. (2017). Polar Nature
of (CH_3_NH_3_)_3_Bi_2_I_9_ Perovskite-Like Hybrids. Inorg. Chem..

[30] Jin Y. U., Marler B., Salak A. N., Escobar-Castillo M., Benson N., Lupascu D. C. (2025). Perovskite-inspired
low-dimensional
hybrid azetidinium bismuth halides: [(CH_2_)_3_NH_2_]_3_Bi_2_X_9_ (X = I, Br, Cl). Mater. Chem. Front..

[31] Tailor N. K., Satapathi S. (2021). Photosensitive
Dielectric and Conductivity Relaxation
in Lead-Free Cs_3_Bi_2_Cl_9_ Perovskite
Single Crystals. J. Phys. Chem. C.

[32] Wojtaś M., Bator G., Jakubas R., Zaleski J., Kosturek B., Baran J. (2003). Crystal structure, phase transitions and ferroelastic properties
of [(CH_3_)_2_NH_2_]_3_[Bi_2_Cl_9_]. J. Solid State Chem..

[33] Luo W., Wu L. K., Shen H. Y., Li H. K., Xu Z. J., Shi C., Ye H. Y., Miao L. P., Wang N. (2024). Halogen-Regulated *T*
_c_ and X-ray Radiation Detection in 2D Hybrid
Perovskite Ferroelastic Semiconductor. Inorg.
Chem..

[34] Wojciechowska M., Gągor A., Piecha-Bisiorek A., Jakubas R., Ciżman A., Zaręba J. K., Nyk M., Zieliński P., Medycki W., Bil A. (2018). Ferroelectricity
and Ferroelasticity
in Organic Inorganic Hybrid (Pyrrolidinium)_3_[Sb_2_Cl_9_]. Chem. Mater..

[35] Kim S. W., Zhang R., Halasyamani P. S., Hayward M. A. (2015). K_4_Fe_3_F_12_: An Fe^2+^/Fe^3+^ Charge-Ordered,
Ferrimagnetic Fluoride with a Cation-Deficient, Layered Perovskite
Structure. Inorg. Chem..

[36] Block T., Seidel S., Pöttgen R. (2022). Bärnighausen
Trees –
A group–subgroup reference database. Z. Kristallogr. - Cryst. Mater..

[37] Müller U. (1998). Strukturverwandtschaften
zwischen trigonalen Verbindungen mit hexagonal-dichtester Anionen-Teilstruktur
und besetzten Oktaederlücken. Berechnung der Anzahl möglicher
Strukturtypen. II [1]. Z. Anorg. Allg. Chem..

[38] Bock O., Müller U. (2002). Symmetrieverwandtschaften
bei Varianten des Perowskit-Typs. Acta Crystallogr.,
Sect. B:Struct. Sci..

[39] Aroyo M. I., Perez-Mato J. M., Orobengoa D., Tasci E., de la Flor G., Kirov A. (2011). Crystallography online:
Bilbao crystallographic server. Bulg. Chem.
Commun..

[40] Aroyo M. I., Perez-Mato J. M., Capillas C., Kroumova E., Ivantchev S., Madariaga G., Kirov A., Wondratschek H. (2006). Bilbao Crystallographic
Server: I. Databases and crystallographic computing programs. Z. Kristallogr. - Cryst. Mater..

[41] Aroyo M. I., Kirov A., Capillas C., Perez-Mato J., Wondratschek H. (2006). Bilbao Crystallographic Server. II.
Representations
of crystallographic point groups and space groups. Acta Crystallogr., Sect. A: Found. Crystallogr..

[42] Lin Y. P., Hu S., Xia B., Fan K. Q., Gong L. K., Kong J. T., Huang X. Y., Xiao Z., Du K. Z. (2019). Material Design
and Optoelectronic Properties of Three-Dimensional Quadruple Perovskite
Halides. J. Phys. Chem. Lett..

[43] Szklarz P., Jakubas R., Gągor A., Bator G., Cichos J., Karbowiak M. (2020). [NH_2_CHNH_2_]_3_Sb_2_I_9_: A lead-free and low-toxicity organic–inorganic
hybrid ferroelectric based on antimony­(iii) as a potential semiconducting
absorber. Inorg. Chem. Front..

[44] Szklarz P., Gągor A., Jakubas R., Zieliński P., Piecha-Bisiorek A., Cichos J., Karbowiak M., Bator G., Ciżman A. (2019). Lead-free
hybrid ferroelectric material
based on formamidine: [NH_2_CHNH_2_]_3_Bi_2_I_9_. J. Mater. Chem.
C.

[45] Belkyal I., Mokhlisse R., Tanouti B., Hesse K. F., Depmeier W. (1997). Crystal structure
of tris (mono-methylammonium) nonachlorodibismuthate (III),(CH_3_NH_3_)_3_Bi_2_Cl_9_. Z. Kristallogr. - Cryst. Mater..

[46] Ashbrook, S. ; Senn, M. ; Amano, M. ; Owen, L. 42nd RSC Solid State Chemistry Group Christmas Meeting; RSC Solid-State Chemistry Group: Edinburgh, UK, 2023; p CO10.

[47] Chabot B., Parthe E. (1978). Cs_3_Sb_2_I_9_ and Cs_3_Bi_2_I_9_ with the hexagonal Cs_3_Cr_2_Cl_9_ structure type. Acta Crystallogr. B Struct. Sci. Cryst. Eng. Mater..

[48] Saillant R., Wentworth R. (1968). Magnetic and
spectroscopic studies of salts of M_2_X_9_
^3–^. Inorg.
Chem..

[49] Chen J., Zhang X., Cai Z., Zhang Y., Song Q., Hua X.-N., Sun B. (2024). Intermolecular Forces Regulating
the Phase-Transition Temperatures in Organic–Inorganic Hybrid
Materials. Inorg. Chem..

[50] Rok M., Zarychta B., Zareba J. K., Krupinska A., Dziuk B., Durlak P., Janicki R., Jakubas R., Bator G., Medycki W., Zamponi M., Piecha-Bisiorek A. (2024). Ferroelectric,
Switchable Dielectric and Nonlinear Optical Properties in Inorganic-Organic
Lead-Free 1D Hybrids Based on Bi­(III) and Azetidine: (C_3_NH_8_)_2_[BiCl_5_], (C_3_NH_8_)_2_[BiBr_5_]. J.
Phys. Chem. Lett..

[51] Jakubas R., Bator G., Sobczyk L., Mróz J. (1994). Dielectric
and pyroelectric properties of (CH_3_NH_3_)_3_Me_2_Br_9_ (Me= Sb, Bi) crystals in the
ferroelectric phase transition regions. Ferroelectrics.

[52] Bator G., Jakubas R., Sobczyk L., Mroaz J. (1993). Dielectric and pyroelectric
properties of [N­(CH_3_)_3_H]_3_Sb_2_Cl_9_ in the low temperature region. Ferroelectrics.

[53] Lang T., Fang S., Han T., Wang M., Yang D., Wang J., Cao S., Peng L., Liu B., Cai M., Zhong Y., Korepanov V. I., Yakovlev A. N. (2020). Phase Transformation
of a K_2_GeF_6_ Polymorph for Phosphors Driven by
a Simple Precipitation-Dissolution Equilibrium and Ion Exchange. Inorg. Chem..

[54] Sahoo A., Paul T., Pal P., Makani N. H., Ghosh A., Banerjee R. (2023). Dielectric Relaxation
Mechanism in the Phase-Transition
Region of a Chiral Hybrid Perovskite and Its Piezoelectric-Energy-Harvesting
Properties. Phys. Rev. Appl..

[55] Rowinska M., Piecha-Bisiorek A., Medycki W., Durlak P., Jakubas R., Gagor A. (2023). Structural,
Electric and Dynamic Properties of (Pyrrolidinium)_3_[Bi_2_I_9_] and (Pyrrolidinium)_3_[Sb_2_I_9_]: New Lead-Free, Organic-Inorganic Hybrids
with Narrow Band Gaps. Molecules.

[56] Fabini D. H., Hogan T., Evans H. A., Stoumpos C. C., Kanatzidis M. G., Seshadri R. (2016). Dielectric and Thermodynamic Signatures
of Low-Temperature
Glassy Dynamics in the Hybrid Perovskites CH_3_NH_3_PbI_3_ and HC­(NH_2_)_2_PbI_3_. J. Phys. Chem. Lett..

[57] Onodera Y., Sawashima N. (1991). Correlation
between transition entropy and the Rhodes-Wohlfarth
ratio in ferroelectric materials. J. Phys. Soc.
Jpn..

[58] Flerov I. N., Gorev M. V., Grannec J., Tressaud A. (2002). Role of metal fluoride
octahedra in the mechanism of phase transitions in A_2_BMF_6_ elpasolites. J. Fluor. Chem..

[59] Onoda-Yamamuro N., Matsuo T., Suga H. (1990). Calorimetric and IR spectroscopic
studies of phase transitions in methylammonium trihalogenoplumbates
(II). J. Phys. Chem. Solids.

[60] Butler K. T., Walsh A., Cheetham A. K., Kieslich G. (2016). Organised chaos: Entropy
in hybrid inorganic-organic systems and other materials. Chem. Sci..

[61] Campbell B. J., Stokes H. T., Tanner D. E., Hatch D. M. (2006). ISODISPLACE: A web-based
tool for exploring structural distortions. J.
Appl. Crystallogr..

[62] Stokes, H. T. ; Hatch, D.M. ; Campbell, B. J. ISODISTORT; ISOTROPY Software Suite, 2023. iso.byu.edu.

[63] Toby B. H., Von Dreele R. B. (2013). GSAS-II: The genesis of a modern
open-source all purpose
crystallography software package. J. Appl. Crystallogr..

[64] Pradhan A., Jena M. K., Samal S. L. (2022). Understanding of the Band Gap Transition
in Cs_3_Sb_2_Cl_9–x_Br_x_: Anion Site Preference-Induced Structural Distortion. ACS Appl. Energy Mater..

[65] Gray M. B., Majher J. D., Holzapfel N. P., Woodward P. M. (2021). Exploring the Stability
of Mixed-Halide Vacancy-Ordered Quadruple Perovskites. Chem. Mater..

[66] Makula P., Pacia M., Macyk W. (2018). How To Correctly
Determine the Band
Gap Energy of Modified Semiconductor Photocatalysts Based on UV-Vis
Spectra. J. Phys. Chem. Lett..

[67] Ning W., Gao F. (2019). Structural and Functional Diversity in Lead-Free Halide Perovskite
Materials. Adv. Mater..

