# Supplementary material for: Structural Polytypism in B‑Site-Deficient Azetidinium-Based Pnictogen Halide Hexagonal Perovskites
Source: Inorg Chem. 2025 Jun 26;64(27):13837–51. doi: 10.1021/acs.inorgchem.5c01374 (PMC12264971; doi:10.1021/acs.inorgchem.5c01374)
Supplement: Supplementary file 1 [file ic5c01374_si_001.pdf]

Supporting information for:

Structural polytypism in B-site deficient azetidinium-based pnictogen halide hexagonal perovskites

Hang Liu[1,*], Rebecca Rae[2], James Dalzell[3], Gavin S. Peters[1], Herbert Früchtl[1], Aidan P. McKay[1], David B. Cordes[1], Amit Kumar[3], Caroline A. Kirk[2], Finlay D. Morrison[1,*].

[1] EaStCHEM School of Chemistry, University of St Andrews, St Andrews, UK.

[2] EaStCHEM School of Chemistry, University of Edinburgh, Edinburgh EH9 3FJ, UK.

[3] Centre for Quantum Materials and Technologies, School of Mathematics and Physics, Queen's University Belfast, Belfast BT7 1NN, UK.

*email: finlay.morrison@st-andrews.ac.uk

Table of contents

S1. Sample preparation 3
S2. Experimental Methods....................3
S2.1 Single crystal X-ray diffraction 3
S2.2 Powder X-ray diffraction 4
S2.3 Compositional Analysis....................4
S2.4 Ultraviolet–visible absorbance spectroscopy....................4
S2.5 Dielectric spectroscopy....................4
S2.6 DSC Measurements 4
S2.7 Piezoresponse force microscopy....................5
S2.8 Computational methodology....................5
S3. Supplementary results 6
S3.1 Single crystal X-ray diffraction data 6
S3.2 Powder X-ray diffraction (PXRD) results 8
S3.4 Energy-dispersive X-ray spectroscopy (EDS) results....................21
S3.5 Dielectric spectroscopy results 23
S3.6 DSC and DTA results....................24
S3.7 Optical absorbance results 27
S3.8 Computational results based on DFT 27
S4. Supplementary Discussions....................31
S4.1 Discussion on crystal structures 31
S5. References....................34

S1. Sample preparation

Table S1.1 Preparation conditions for $Az_3Sb_2Cl_{9-x}Br_x$.

Sample	$SbCl_3$ (mmol)	$SbBr_3$ (mmol)	37% HCl (ml)	48% HBr (ml)
$Az_3Sb_2Cl_9$	4	0	3	0
$Az_3Sb_2Cl_{8.5}Br_{0.5}$	3.107	0.8931	3	0
$Az_3Sb_2Cl_8Br_1$	2.214	1.786	3	0
$Az_3Sb_2Cl_{7.5}Br_{1.5}$	1.321	2.679	3	0
$Az_3Sb_2Cl_7Br_2$	0.428	3.572	3	0
$Az_3Sb_2Cl_7Br_2$ (s)	1.282	10.718	9	0
$Az_3Sb_2Cl_6Br_3$	3.601	0.399	1.5	1.5
$Az_3Sb_2Cl_{4.5}Br_{4.5}$	1.192	2.808	1.5	1.5
$Az_3Sb_2Cl_3Br_6$	0.547	3.453	1	2
$Az_3Sb_2Cl_2B_{r7}$	2.853	1.147	0	3
$Az_3Sb_2Cl_1Br_8$	1.427	2.573	0	3
$Az_3Sb_2Br_9$	0	4	0	3

S2. Experimental Methods

S2.1 Single crystal X-ray diffraction

Single crystal X-ray diffraction (SCXRD) data all compounds analysed were collected (using a calculated strategy) and processed (including correction for Lorentz, polarization and absorption) using CrysAlisPro.[1] Structures were solved by either, direct (SHELXS[2]), or dual-space methods (SHELXT[2]) and refined by full-matrix least-squares against F^2 (SHELXL-2019/3[3]). Non-hydrogen atoms were refined anisotropically except for azetidinium ions with low occupancy which were refined isotropically. Hydrogen atoms were placed in calculated positions refined using a riding model. All calculations were performed using the Olex2[4] interface. Further details of specific structures are below and selected crystallographic data are presented in Tables S3.1 – S3.2.

SCXRD data of $Az_3Sb_2Cl_9$ were collected at 293 K and 173 K. For the data measured at 293K, the Az^+ ions were positioned on the crystallographic A-site. Due to the orientational disorder disorder arising from the symmetry mismatch between Az^+ and the high-symmetry sites, the organic cations were positioned using a calculated model and refined isotropically with occupancy fixed at 1/6. For the data measured at 173 K, the positions of the organic cations were determined directly from the electron density map and refined anisotropically subject to geometric and thermal restraints. One cation showed discrete disorder and was modelled in two orientations.

SCXRD data of $Az_3Sb_2Br_9$ were collected at 293 K. The organic cations again showed a symmetry mismatch to their high-symmetry sites, and were modelled in the same manner as in $Az_3Sb_2Cl_9$. Although the SCXRD data of $Az_3Sb_2Br_9$ below the PTs temperatures were collected and indexed in their maximal subgroup $Cmc2_1$ cell, satisfactory refinements could not be obtained, and the indicated structures did not appear to match the structure suggested from the LT-PXRD pattern.

SCXRD data of dimer structures $Az_3Sb_2I_9$, $Az_3Bi_2Br_9$ and $Az_3Bi_2I_9$ were collected at 293 K, 173

K and 100 K. At 293 K in all three structures the Az^+ ions again showed a symmetry mismatch to their high-symmetry sites, and were modelled in the same manner as $Az_3Sb_2Cl_9$. Many of the cations also displayed additional positional disorder, which was modelled with multiple orientations. In the structure of $Az_3Sb_2I_9$ at 173 K both Az^+ ions were placed using the calculated model with one at full occupancy and one showing a symmetry mismatch and refined with occupancy fixed at ½. At both 173 K and 100 K for the remaining structures, the organic cations were located directly from the electron density map, and were refined anisotropically with minimal geometric restraints.

SCXRD data of one-dimensional trans-chain structures Az_2BiCl_5 and Az_2BiBr_5 were both collected at 293 K. The organic cations again showed a symmetry mismatch to their high-symmetry sites, and were modelled in the same manner as $Az_3Sb_2Cl_9$, except with anisotropic refinement with occupancies fixed at ½. In both structures one of the cations displayed additional positional disorder, which was modelled with two orientations with their occupancies summed to ½. Although SCXRD data below PT temperatures for both compounds were collected, reasonable refinements were unable to be obtained from either the suggested monoclinic space group (suggested by variable temperature PXRD) or other potential space groups.

S2.2 Powder X-ray diffraction

Rietveld analysis of PXRD data was performed to confirm the phase purity and determine the lattice parameters using the General Structure Analysis System (GSAS-II) software.[5] Details including treatment of preferred orientation are given in section S3.2.

S2.3 Compositional Analysis

Determination of chemical composition in $Az_3Sb_2Cl_{9-x}Br_x$ was performed on the powder sample via energy-dispersive X-ray spectroscopy (EDS) by using a JEOL JSM-IT200 scanning electron microscope (SEM) with an accelerating voltage of 15 kV. The chemical composition of each sample was estimated by both point sampling and map sampling to characterize the distribution of compositional ratio between Cl and Br. The EDS results are listed in Table S3.8.

S2.4 Ultraviolet–visible absorbance spectroscopy

Pseudo-absorbances was estimated from steady-state UV-vis diffuse reflectance data measured by using a JASCO-V650 double-beam spectrophotometer. The optical band gap of powder sample was determined based on the Kubelka-Munk transformation after correction for the Teflon sample holder.[6, 7]

S2.5 Dielectric spectroscopy

Dielectric spectroscopy was carried out on the pellet sample prepared from dried powder under uniaxial loading of ca. 1 ton. Silver electrodes were applied to the opposite sides of pellet surfaces and dried in a drying oven at 120 °C for 1h.

S2.6 DSC Measurements

For DSC measurements, note that during initial cooling from room temperature a significant "exothermic"-like peak appears in the DSC trace (see e.g. Figures 12 (a) and (c) and Figures S3.15 (a) and (b)); this is an experimental artefact and is present even in calibration runs with empty pans. In instances where this peak coincided with a phase transition, the data collect run was started at a temperature higher than room temperature, e.g. $Az_3Bi_2Br_9$ Figure 12 (b),

in order to avoid overlap.

S2.7 Piezoresponse force microscopy

Single point measurements were carried out across each sample with the amplitude response recorded at each frequency for a total of 50 seconds, before the voltage was increased within the same scan for visualisation purposes. The amplitude response was averaged for each applied voltage and compared against two reference samples: periodically-poled $LiNbO_3$, (large effective d_{33}) and non-piezoelectric glass. These references allowed estimation of the effective d_{33} of $Az_3Sb_2Cl_9$ and $Az_3Sb_2Br_9$.

S2.8 Computational methodology

All density functional based calculations were carried out using the CASTEP version 22.11 with the program's standard default on-the-fly pseudopotentials.[8] The exchange–correlational energy functional was approximated within the generalized-gradient approximation (GGA) framework using Perdew-Burke-Ernzerhof functional for solids (PBESol) parametrization. For simplification, scalar relativistic potentials without spin-orbit coupling were used for all geometry optimizations and electronic structure calculations. We used a plane wave cut-off of 32Ry for the wave functions and a Γ-centered Monkhorst-Pack (MP) 6 × 6 × 4 k-point grid for the Brillouin zone (BZ) sampling for all calculations of RT $Az_3Sb_2Cl_9$, RT $Az_3Sb_2Br_9$, primitive RT $Az_3Sb_2I_9$, primitive RT $Az_3Bi_2Br_9$ and primitive RT $Az_3Bi_2I_9$. The selection of high symmetry points follows the suggestions from SeeK-path.[9] All calculations were performed by using the crystal structure obtained from single crystal XRD at room temperature.

Due to the disordered nature or the organic molecule observed in the room temperature crystal structures, the calculations on all $Az_3B_2X_9$ structure were approximated via different strategies. For rotational disorder observed in $P6_3mc$ $Az_3Sb_2Cl_9$ and $Az_3Sb_2Br_9$, the azetidinium cations occupy crystallographic sites with either 6_3 screw axis or threefold rotation axis symmetry. General methods to treat this type of disorder involved replacing the Az^+ by symmetry compatible hypothesized organic molecules (e.g. benzene) or using inorganic Cs^+. Both approximations may lead to different results in the geometry optimization and the following calculations on the electronic structure. In this work, Cs^+ was used in place of Az^+ in DFT calculations for $Az_3Sb_2Cl_9$ and $Az_3Sb_2Br_9$ as this method has been used for approximation in the similar DFT calculations on $AzPbBr_3$ and $Az_2AgBiBr_6$.[10, 11] For the Az positional disorder observed in *Cmcm* $Az_3Sb_2I_9$, $Az_3Bi_2Br_9$ and $Az_3Bi_2I_9$, a primitive cell was used in all calculations to represent the C-centered structure. In this primitive cell model the Az positions were based on those of the low temperature (primitive) crystal structure Phase II with *Pbcm* space group as determined by single crystal XRD, and the fractional positions of the azetidinium cation were modelled accordingly.

S3. Supplementary results

S3.1 Single crystal X-ray diffraction data

Table S3.1. Selected crystallographic data for $Az_3Sb_2Cl_9$, $Az_3Sb_2Br_9$, $Az_3Sb_2I_9$ and Az_2BiCl_5.

	**$Az_3Sb_2Cl_9$**		**$Az_3Sb_2Br_9$**	**$Az_3Sb_2I_9$**			**Az_2BiCl_5**
	**293 K**	**173 K**	**293 K**	**293 K**	**173 K**	**100 K**	**293 K**
formula	$C_9H_{24}Cl_9N_3Sb_2$	$C_9H_{24}Cl_9N_3Sb_2$	$C_9H_{24}Br_9N_3Sb_2$	$C_9H_{24}I_9N_3Sb_2$	$C_9H_{24}I_9N_3Sb_2$	$C_9H_{24}I_9N_3Sb_2$	$C_6H_{15}Cl_5N_2Bi$
formula weight [g/mol]	736.86	736.86	1137.00	1559.91	1559.91	1559.91	501.43
crystal description	Colourless prism	Colourless prism	Yellow plate	Yellow plate	Yellow plate	Yellow plate	Colourless needle
crystal size [mm^3]	0.07 × 0.06 × 0.04	0.07 × 0.06 × 0.04	0.03 × 0.02 × 0.01	0.09 × 0.06 × 0.01	0.09 × 0.09 × 0.01	0.035 × 0.03 × 0.007	0.12 × 0.01 × 0.01
space group	*P*6$_3$*mc*	*Pna*2$_1$	*P*6$_3$*mc*	*Cmcm*	*Pbcm*	*Pbcm*	*Pnma*
a [Å]	8.5621(4)	14.6856(7)	8.7372(4)	9.9459(8)	8.7725(2)	8.7549(8)	10.9482(4)
b [Å]		8.5128(3)		15.0414(10)	16.9496(5)	16.8680(16)	8.1168(3)
c [Å]	19.9943(9)	19.7370(10)	20.8778(10)	21.3898(16)	20.9318(5)	20.849(2)	17.1337(7)
vol [Å]^3	1269.40(13)	2467.43(19)	1380.24(13)	3199.9(4)	3112.36(14)	3078.9(5)	1522.58(10)
Z	2	4	2	4	4	4	4
ρ (calc) [g/cm^3]	1.928	1.984	2.736	3.238	3.329	3.365	2.187
μ [mm^{-1}]	3.075	3.164	14.985	10.374	10.666	10.781	12.428
F(000)	708	1416	1032	2712	2712	2712	932
reflections collected	8736	28618	15117	25431	33207	116226	16919
ind. reflections (R_{int})	1099 (0.0300)	16056 (0.0385)	1215 (0.0248)	2162 (0.1423)	3908 (0.0333)	9476 (0.0572)	2021 (0.0419)
parameters, restraints	73, 25	245, 198	73, 27	96, 42	127, 32	113, 0	143, 155
GooF on F^2	1.054	1.060	1.077	0.963	1.071	1.076	1.030
R_1 [$I > 2\sigma(I)$][a]	0.0239	0.0769	0.0660	0.0960	0.0240	0.0352	0.0366
wR_2 (all data)[a]	0.0573	0.2227	0.1631	0.2920	0.0533	0.0976	0.0801
max diff peak/hole [e/Å^3]	0.22/-0.26	2.86/-2.31	1.10/-0.49	1.77/-0.90	1.42/-1.03	1.82/-0.94	0.92/-0.54
CCDC Deposition No.	2434075	2434076	2434077	2434078	2434079	2434080	2434087

[a] $R_1 = \frac{\sum||F_o|-|F_c||}{\sum|F_o|}$; $wR_2 = \sqrt{\frac{\sum w(F_o^2-F_c^2)^2}{\sum wF_o^4}}$

Table S3.2. Selected crystallographic data for $Az_3Bi_2Br_9$, $Az_3Bi_2I_9$ and Az_2BiBr_5.

	**$Az_3Bi_2Br_9$**			**$Az_3Bi_2I_9$**			**Az_2BiBr_5**
	**293 K**	**173 K**	**100 K**	**293 K**	**173 K**	**100 K**	**293 K**
formula	$C_9H_{24}Br_9N_3Bi_2$	$C_9H_{24}Br_9N_3Bi_2$	$C_9H_{24}Br_9N_3Bi_2$	$C_9H_{24}Br_9N_3Bi_2$	$C_9H_{24}Br_9N_3Bi_2$	$C_9H_{24}Br_9N_3Bi_2$	$C_6H_{16}Br_5N_2Bi$
formula weight [g/mol]	1311.46	1311.46	1311.46	1734.37	1734.37	1734.37	724.74
crystal description	Yellow block	Yellow block	Yellow block	Red plate	Orange block	Orange block	Yellow plate
crystal size [mm^3]	0.06 × 0.04 × 0.02	0.06 × 0.04 × 0.02	0.06 × 0.04 × 0.02	0.19 × 0.04 × 0.01	0.04 × 0.03 × 0.02	0.04 × 0.03 × 0.02	0.06 × 0.03 × 0.01
space group	*Cmcm*	*Pbcm*	*Pnma*	*Cmcm*	*Pbcm*	*Pnma*	*Pnma*
a [Å]	8.4310(6)	8.4250(2)	15.773(3)	9.6212(5)	8.7765(2)	16.5620(4)	11.1028(4)
b [Å]	16.3350(10)	16.2344(3)	20.598(3)	15.2694(7)	16.8852(4)	21.4666(5)	8.3881(3)
c [Å]	20.5992(14)	20.2055(5)	8.4545(13)	22.0609(10)	21.3016(5)	8.7778(2)	18.0024(7)
vol [Å]^3	2836.9(3)	2763.60(11)	2746.8(8)	3241.0(3)	3156.75(13)	3120.74(14)	1676.59(11)
Z	4	4	4	4	4	4	4
ρ (calc) [g/cm^3]	3.071	3.152	3.171	3.554	3.649	3.691	2.871
μ [mm^{-1}]	25.066	25.731	25.888	19.420	19.938	20.168	22.407
F(000)	2320.0	2320	2320	2968.0	2968	2968	1296
reflections collected	27768	46250	68331	26886	34001	56804	18271
ind. reflections (R_{int})	1977 (0.1045)	3605 (0.0452)	12474 (0.1098)	2185 (0.0371)	4088 (0.0306)	10077 (0.0974)	2185 (0.0281)
parameters, restraints	140, 141	112, 2	114, 1	64, 25	112, 8	113/0	143, 111
GooF on F^2	1.048	1.157	1.050	1.100	1.086	0.945	1.115
R_1 [$I > 2\sigma(I)$]	0.0554	0.0376	0.0738	0.0858	0.0351	0.0665	0.0880
wR_2 (all data)	0.1734	0.0930	0.2021	0.2372	0.0748	0.1641	0.2209
largest diff. peak/hole [e/Å^3]	1.51/-1.07	1.99/-1.12	4.36/-3.06	2.48/-0.56	2.98/-1.44	6.45/-1.91	2.77/-0.54
CCDC Deposition No.	2434081	2434082	2434083	2434084	2434085	2434086	2434088

[a] $R_1 = \frac{\sum ||F_o|-|F_c||}{\sum |F_o|}$; $wR_2 = \sqrt{\frac{\sum w(F_o{}^2-F_c{}^2)^2}{\sum wF_o{}^4}}$

S3.2 Powder X-ray diffraction (PXRD) results

RT-PXRD for all compounds

Rietveld refinements were performed on the PXRD data of powder samples for undoped Az_2BiCl_5 and $Az_3M_2X_9$ (M^{3+} = Sb and Bi, X^- = Cl, Br and I) to check the crystal structure determined from SCXRD and confirm phase purity for further properties analysis. Background profiles were refined using a **12-term Chebyschev-1 function**, and a Pseudo-Voigt function used for peak profiles. In addition to Az_2BiCl_5, all $Az_3M_2X_9$ patterns show typical characteristics of preferred orientation along (00L) due to the hcp nature of the crystal structure. A spherical harmonics model with a maximum 12 harmonic orders was used to model preferred orientation. The PXRD pattern for all compounds confirm the crystal structure determined by SCXRD at RT.

The PXRD pattern of Az_2BiBr_5 powder sample shows multiple phases at RT, Figure S3.7. The structural determination of the secondary phase is not possible as only one extra peak can be assigned to other phases with high certainty. However, based on the onset transition temperature from the DSC data, Figure S3.15, this impurity could reasonably be caused by co-precipitation of $Az_3Bi_2Br_9$. Although the PXRD data of Az_2BiBr_5 powder sample can be refined using the structure determined from SCXRD, a large difference is observed in the fitted intensity of several peaks, which may be related to preferred orientation.

Table S3.3 RT-PXRD refinements for azetidinium antimony analogues

Formula	$(C_3H_8N)_3Sb_2Cl_9$	$(C_3H_8N)_3Sb_2Br_9$	$(C_3H_8N)_3Sb_2I_9$
T (K)	298	298	298
Formula wt (g/mol)	736.92	1136.98	1559.95
Crystal system	hexagonal	hexagonal	orthorhombic
Space group	$P6_3mc$ (186)	$P6_3mc$ (186)	*Cmcm* (63)
a (Å)	8.5834(4)	8.76931(24)	9.95973(32)
b (Å)	8.5834(4)	8.76931(24)	15.0403(5)
c (Å)	19.9705(6)	20.8981(3)	21.3835(4)
Volume ($Å^3$)	1274.19(9)	1391.77(6)	3203.20(21)
Density (calc., g/cm^3)	1.9207	2.7131	3.2347
θ range (deg)	3 - 70	3 - 70	3 - 70
Goodness of fit on F2	2.16	2.53	1.58
Reduced χ^2	4.67	6.41	2.49
wR_2 (all data)	6.957	8.987	7.942

Table S3.4 RT-PXRD refinements for azetidinium bismuth analogues

Formula	$(C_3H_8N)_2BiCl_5$	$(C_3H_8N)_3Bi_2Br_9$	$(C_3H_8N)_3Bi_2I_9$
T (K)	298	298	298
Formula wt (g/mol)	502.45	1311.41	1734.41
Crystal system	orthorhombic	orthorhombic	orthorhombic
Space group	*Pnma* (62)	*Cmcm* (63)	*Cmcm* (63)
a (Å)	11.00616(12)	8.44192(19)	9.5877(5)
b (Å)	8.12041(10)	16.3237(4)	15.3419(8)
c (Å)	17.16064(20)	20.8244(4)	22.1240(8)
Volume (Å^3)	1533.72(4)	2869.67(13)	3254.3(3)
Density (calc., g/cm^3)	2.1760	3.0354	3.5400
θ range (deg)	3 - 70	3 - 70	3 - 70
Goodness of fit on F2	2.22	1.78	1.66
Reduced χ^2	4.91	3.18	2.75
wR_2 (all data)	6.830	5.823	8.066

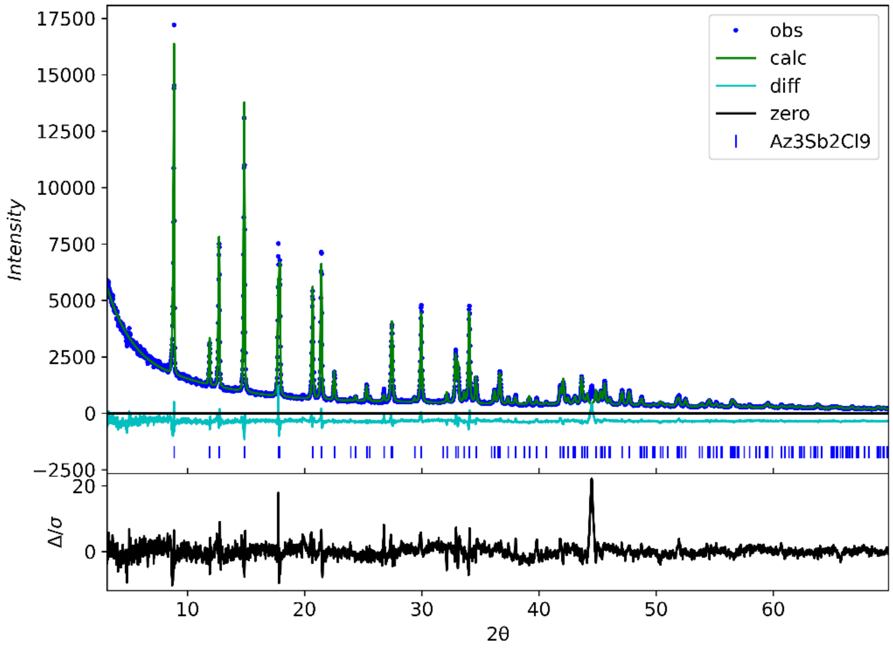

Figure S3.1 GSAS-II refinement profile for RT-PXRD $Az_3Sb_2Cl_9$

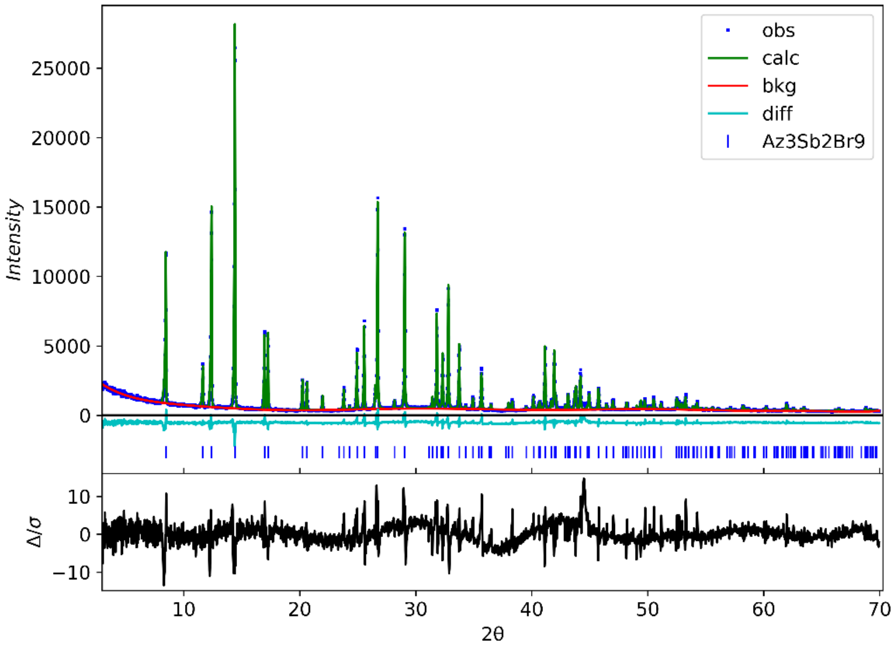

Figure S3.2 GSAS-II refinement profile for RT-PXRD $Az_3Sb_2Br_9$

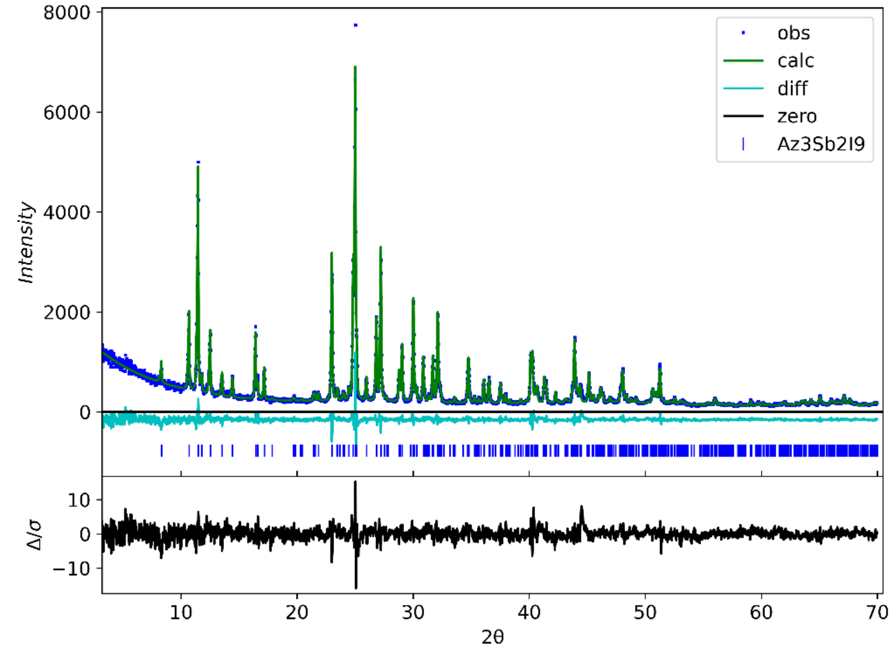

Figure S3.3 GSAS-II refinement profile for RT-PXRD $Az_3Sb_2I_9$

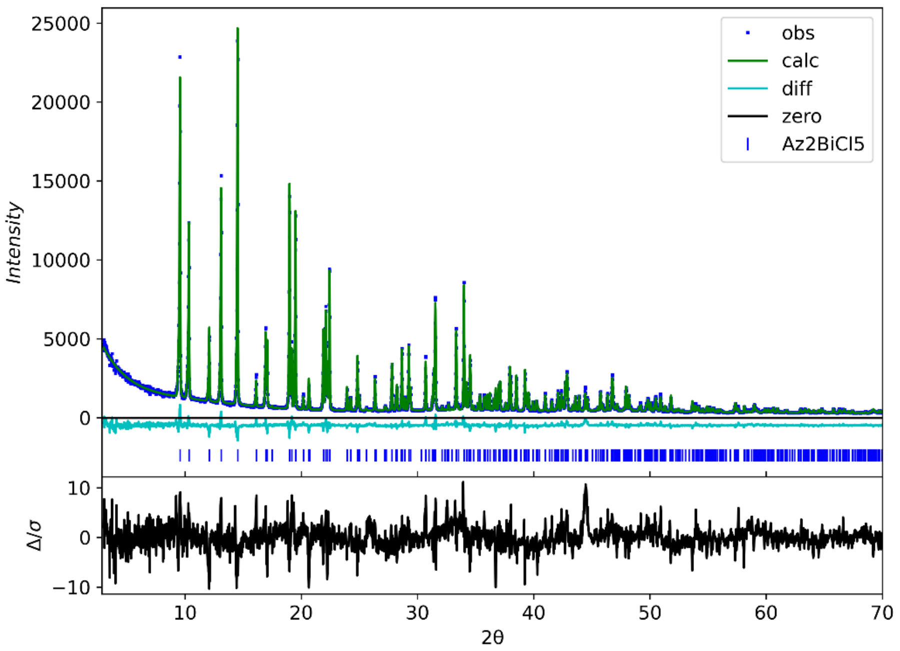

Figure S3.4 GSAS-II refinement profile for RT-PXRD Az_2BiCl_5

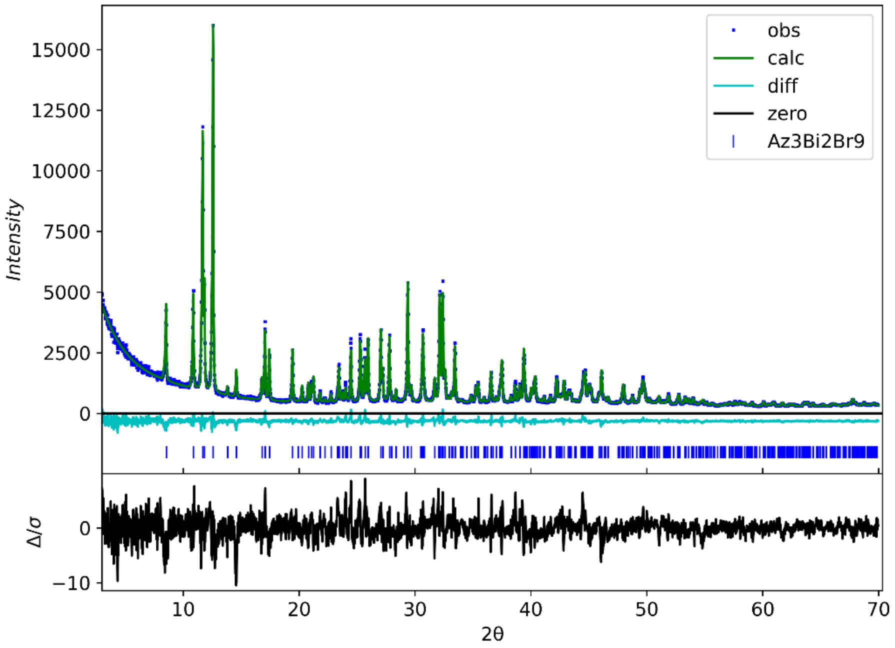

Figure S3.5 GSAS-II refinement profile for RT-PXRD $Az_3Bi_2Br_9$

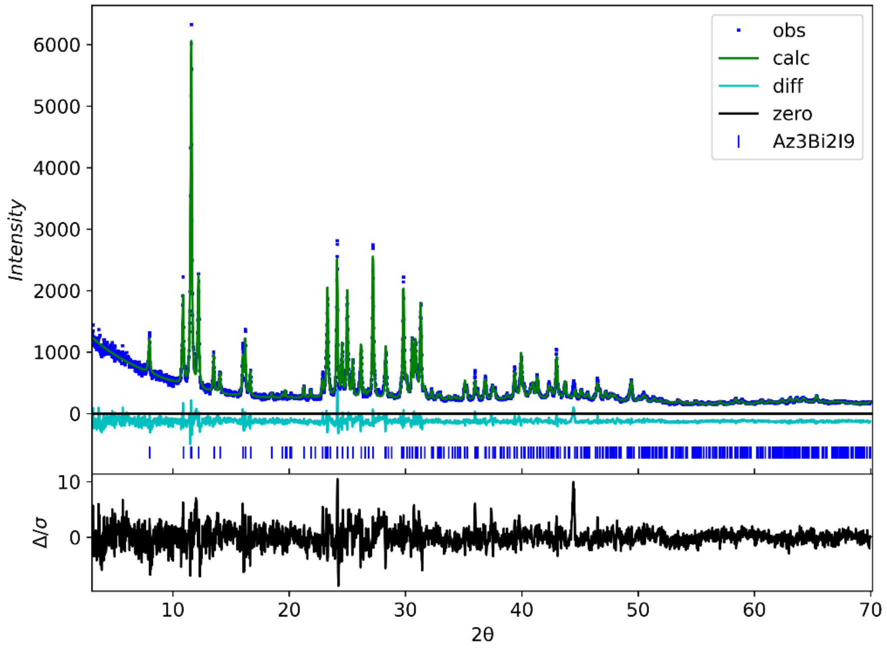

Figure S3.6 GSAS-II refinement profile for RT-PXRD $Az_3Bi_2I_9$

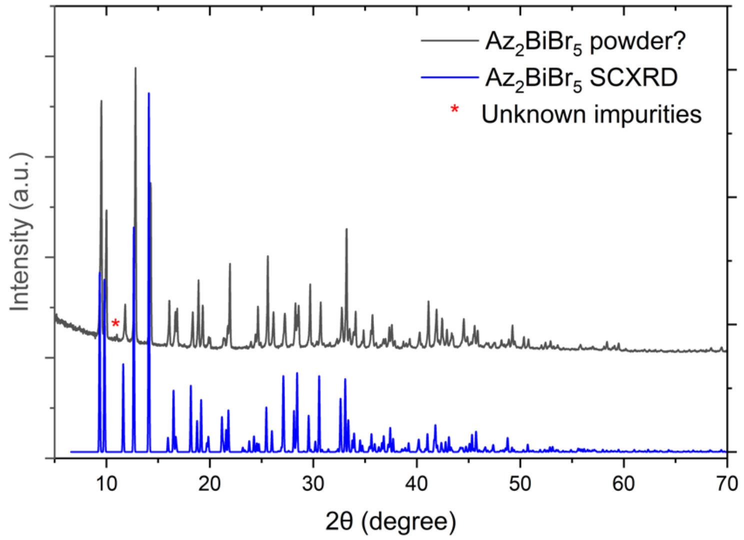

Figure S3.7 PXRD pattern for powder sample Az_2BiBr_5

VT-PXRD for $Az_3Sb_2Cl_9$ and $Az_3Sb_2Br_9$

Rietveld refinements were performed on the PXRD of powder samples for $Az_3Sb_2Cl_9$ and $Az_3Sb_2Br_9$ to investigate the structural evolution at low temperatures. In order to further check reproducibility, VT-PXRD data was collected using two different powder samples in two different experiments for both $Az_3Sb_2Cl_9$ and $Az_3Sb_2Br_9$. For the former, data was collected at 240 K, 230 K, 100 K to 20 K in the first experiment and 298 K to 260 K, 220 K to 110 K in the second. For $Az_3Sb_2Br_9$ data collections were 270 K to 240 K for the first sample and 298 K, 230 K to 60 K for the second. All data exhibited significant but different extents of preferred orientation along 00L due to the closed packed nature of 6H perovskite structures and were refined using a March-Dollase model, but nevertheless returned the same lattice constants for each composition.

As mentioned in the main manuscript, the limitation of Cu-based PXRD, strong preferred orientation and uncertainties related to organic molecular configurations inhibits further determination of distortion mode via ISODISTORT.[12, 13] Hence, the PXRD refinements on $Az_3Sb_2Cl_9$ and $Az_3Sb_2Br_9$ were carried out based on different strategies.

For $Az_3Sb_2Cl_9$, the orthorhombic $Pna2_1$ phase II can be well-defined by SCXRD and thus be used to model the superstructure orthorhombic $Pna2_1$ phase III by tripling the cell along the *a*-axis. Subsequently, the PXRD pattern measured at 220 K and 210 K were refined using orthorhombic $Pna2_1$ phase II to confirm the space group and the distortion mode observed in SCXRD. Whilst, the PXRD data at lower temperatures than 210 K were refined using the superstructure orthorhombic $Pna2_1$ phase III without

any further refinements on either atomic positions or U_{iso} parameters. Due to the relatively low intensity of the superstructure peaks appeared in the PTs from II to III, the consequences of ignoring superstructure peaks are negligible on the accuracy of the lattice parameters. For the data collected below 90 K, it is evident for the structure to show at least monoclinic distortion due to the splitting of the (011) peak but any refinement on such a large monoclinic or triclinic cell was unstable. All refinement results on VT-PXRD data of $Az_3Sb_2Cl_9$ are summarized in Table S3.5. One typical refinement for PXRD data measured on 155 K is attached in Figure S3.8.

Table S3.5 PXRD refinement details for VT-PXRD on $Az_3Sb_2Cl_9$

T(K)	Space group	a(Å)	b(Å)	c(Å)	V(Å^3)	wR^2	GOF	Reduced χ^2
296	$P6_3mc$	8.5583	8.5583	19.982	1267.5	17.6	1.29	1.66
280	$P6_3mc$	8.5526	8.5526	19.956	1264.1	18.4	1.35	1.82
269	$P6_3mc$	8.5475	8.5475	19.939	1261.5	18.6	1.36	1.86
260	$P6_3mc$	8.5428	8.5428	19.921	1259.0	18.8	1.38	1.90
240	$P6_3mc$	8.5351	8.5351	19.898	1255.3	17.3	1.27	1.61
230	$P6_3mc$	8.5295	8.5295	19.879	1252.5	17.2	1.26	1.59
220	$Pna2_1$	14.699	8.5360	19.857	2491.4	14.1	2.06	4.23
210	$Pna2_1$	14.689	8.5362	19.828	2486.1	13.6	1.98	3.92
200	$Pna2_1$	44.117	8.5385	19.831	7469.9	23.5	1.72	2.95
190	$Pna2_1$	44.006	8.5363	19.770	7426.6	14.2	2.06	4.26
180	$Pna2_1$	43.974	8.5354	19.751	7413.2	14.2	2.05	4.22
170	$Pna2_1$	43.940	8.5340	19.735	7400.1	13.8	2.01	4.02
155	$Pna2_1$	43.894	8.5304	19.717	7382.7	14.0	2.02	4.08
140	$Pna2_1$	43.846	8.5256	19.702	7365.1	14.0	2.02	4.07
125	$Pna2_1$	43.807	8.5187	19.687	7346.6	13.3	1.90	3.62
110	$Pna2_1$	43.806	8.5121	19.629	7319.3	13.3	1.89	3.58
100	$Pna2_1$	43.888	8.4951	19.569	7296.1	20.5	2.47	6.12
90	$Pna2_1$	43.880	8.4927	19.545	7283.7	19.8	2.38	5.66
80	$Pna2_1$	43.854	8.4956	19.513	7269.9	23.2	2.78	7.72
70	$Pna2_1$	43.825	8.5009	19.483	7258.5	28.0	3.55	11.24
60	$Pna2_1$	43.808	8.5021	19.467	7250.8	30.5	3.64	13.22
20	$Pna2_1$	43.818	8.4982	19.449	7242.2	33.9	2.46	6.03

For $Az_3Sb_2Br_9$, the modelling of low temperature orthorhombic structure is based on the parent hexagonal $P6_3mc$ phase I because of the inability to refine SCXRD data for

Phase II, III and IV. Although DSC data suggests an order-disorder nature for all PTs in $Az_3Sb_2Br_9$, establishing an ordered Az^+ model for refinement of LT-PXRD data was not possible given the poor scattering of Az^+ using Cu-based PXRD. Hence, to simplify the modelling process for the refinement of LT-PXRD in $Az_3Sb_2Br_9$, Az^+ was modelled using Mn^{2+} which has the same electron count as Az^+. The crystal structure for Phase IV could then be modelled by the subgroup $Pna2_1$ with the transformation [6 3 0 0 2 0 0 0 1] from the parent structure $P6_3mc$. The solution with the subgroup $Pmn2_1$ by the transformation [2 0 0 3 6 0 0 0 1] is also possible and the reflection conditions for both space groups provide similar refinement results. However, it is not possible to reliably extract any physically meaningful information on the atomic positions and thermal parameters during the PTs in such a large supercell with strong PO and uncertain positions of light atoms. Hence, only $Pna2_1$ was utilized to extract the lattice parameter by refining all allowed parameters in both atomic position and U_{iso} parameters in order to improve the accuracy of lattice parameters extraction. The refinement results are summarized in Table S3.6. One typical refinement result for the 150 K data is attached as Figure S3.9.

Table S3.6 PXRD refinement details for VT-PXRD on $Az_3Sb_2Br_9$

T(K)	Space group	a(Å)	b(Å)	c(Å)	V(Å^3)	wR^2	GOF	Reduced χ^2
298	$P6_3mc$	8.7542	8.7542	20.908	1387.7	11.3	1.54	2.36
270	$P6_3mc$	8.7457	8.7457	20.846	1380.8	17.9	1.41	2.00
260	$P6_3mc$	8.7394	8.7394	20.830	1377.8	17.5	1.38	1.90
240	$P6_3mc$	8.7282	8.7282	20.798	1372.2	14.8	2.16	4.69
230	$P6_3mc$	8.7153	8.7153	20.799	1368.2	11.7	1.57	2.48
220	$P6_3mc$	8.7098	8.7098	20.780	1365.2	11.8	1.59	2.52
210	$P6_3mc$	8.7038	8.7038	20.761	1362.1	11.5	1.54	2.38
205	$P6_3mc$	8.7013	8.7013	20.750	1360.6	11.7	1.56	2.44
202	$P6_3mc$	8.6997	8.6997	20.744	1359.7	11.7	1.56	2.42
199	$P6_3mc$	8.6979	8.6979	20.738	1358.7	11.7	1.56	2.44
196	$P6_3mc$	8.6960	8.6960	20.732	1357.7	11.9	1.59	2.52
193	$P6_3mc$	8.6944	8.6944	20.726	1356.8	11.8	1.57	2.46
190	$P6_3mc$	8.6925	8.6925	20.719	1355.8	12.1	1.60	2.54
187	$P6_3mc$	8.6909	8.6909	20.713	1354.9	12.1	1.59	2.51
184	$P6_3mc$	8.6875	8.6875	20.707	1353.4	13.7	1.80	3.23
180	$Pna2_1$	45.226	17.320	20.701	16214	12.4	1.77	3.12
170	$Pna2_1$	45.177	17.354	20.644	16185	11.3	1.47	2.17

160	*Pna*2$_1$	45.152	17.355	20.613	16153	11.2	1.59	2.52
150	*Pna*2$_1$	45.142	17.346	20.591	16124	10.7	1.52	2.31
140	*Pna*2$_1$	45.131	17.335	20.573	16096	10.7	1.39	1.94
130	*Pna*2$_1$	45.115	17.327	20.557	16069	10.6	1.38	1.90
120	*Pna*2$_1$	45.101	17.316	20.541	16042	10.7	1.39	1.93
110	*Pna*2$_1$	45.083	17.307	20.528	16017	10.9	1.41	1.99
100	*Pna*2$_1$	45.067	17.296	20.513	15989	10.9	1.41	1.98
90	*Pna*2$_1$	45.052	17.285	20.501	15965	10.3	1.33	1.77
80	*Pna*2$_1$	45.043	17.273	20.490	15943	10.6	1.38	1.90
70	*Pna*2$_1$	45.029	17.263	20.480	15920	10.8	1.53	2.34
60	*Pna*2$_1$	45.017	17.256	20.471	15902	10.9	1.52	2.32

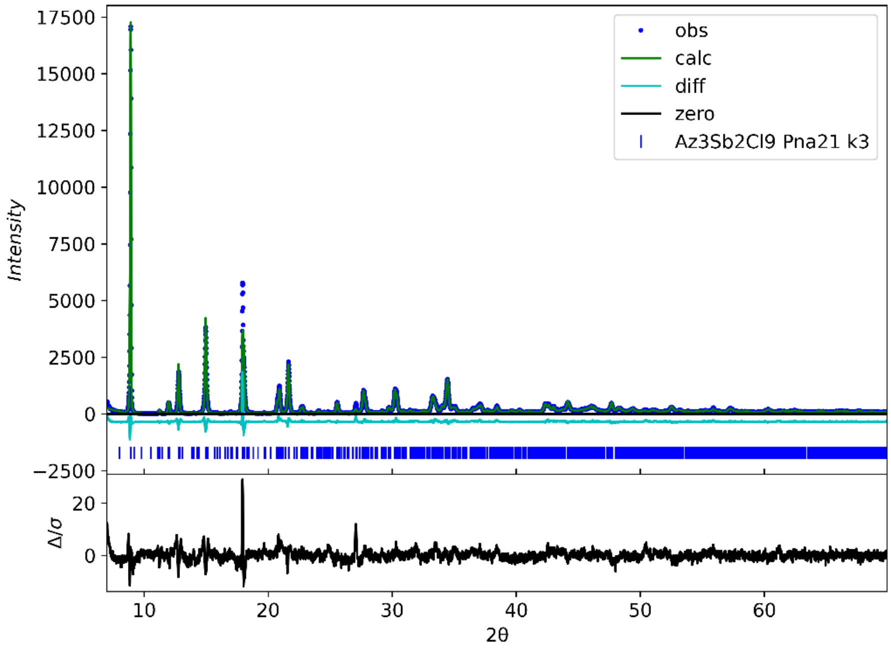

Figure S4.8 GSAS-II refinement profile for 155 K PXRD data for $Az_3Sb_2Cl_9$

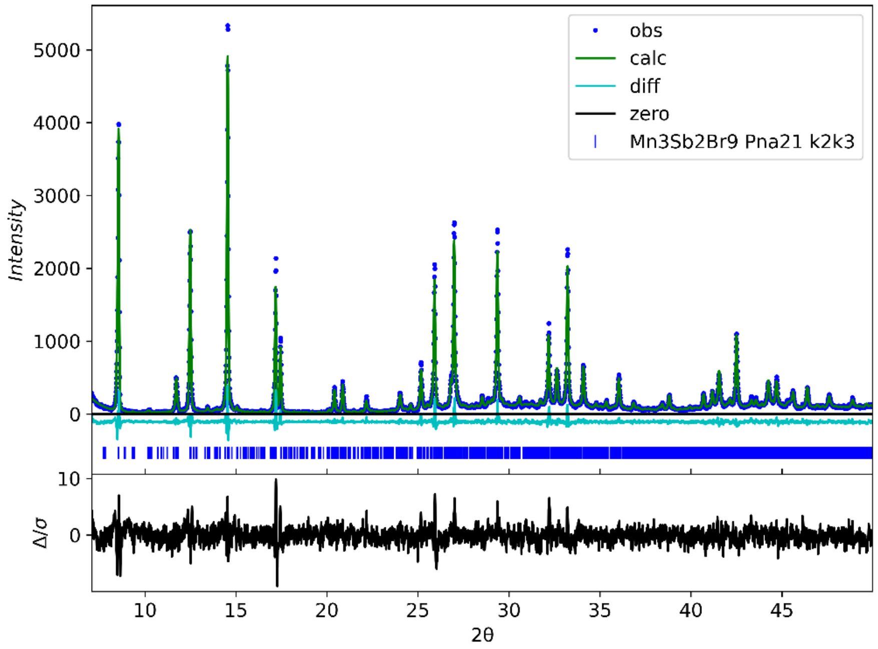

Figure S3.9 GSAS-II refinement profile for 150 K PXRD data for $Az_3Sb_2Br_9$

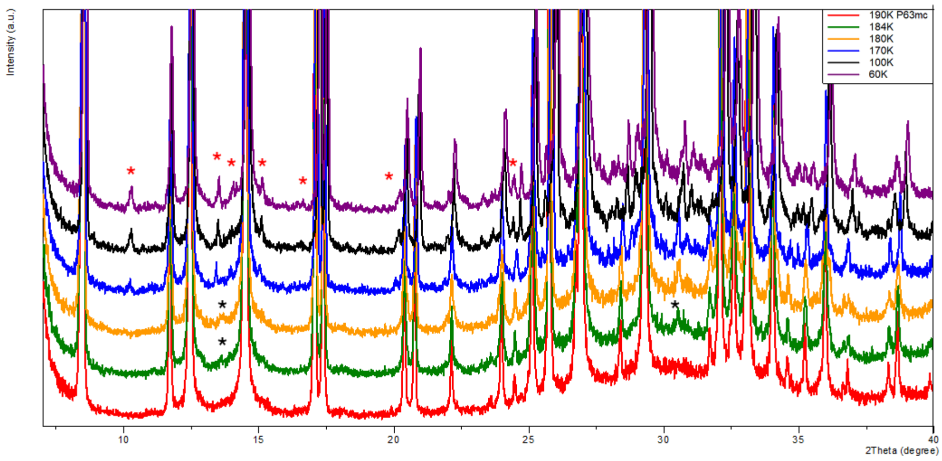

Figure S3.10 LT-PXRD profile for $Az_3Sb_2Br_9$ where black and red asterisks represent the formation of superstructure peaks during the PT (I→II→III) and PT (III→IV) respectively

RT-PXRD for solid solution $Az_3Sb_2Cl_{9-x}Br_x$

Rietveld refinements were also carried out on PXRD data of solid solution $Az_3Sb_2Cl_{9-x}Br_x$ powder samples using the same procedures to investigate the influence of Br incorporation on crystal structure. All data show different extents of PO along 00L and were refined by a spherical harmonics model with a maximum of 12 harmonic orders. The crystal structure for refinements is modelled based on the single crystal structure of $Az_3Sb_2Br_9$ obtained from SCXRD with homogeneous occupancy of Cl at Br site where the occupancy rate is calculated from EDS data (see following section for details). Due to the influence of strong PO, the atomic positions and U_{iso} parameters were not refined to avoid physically meaningless results. The refinement results based on a homogenous (random) halide distribution are attached in Table S3.7

Table S3.7 GSAS-II refinement details for RT-PXRD on $Az_3Sb_2(Cl_{1-x}Br_x)_9$

**Target composition**	**Proportion Br used in synthesis**	**Proportion Br from EDS**	***a* (Å)**	***c* (Å)**	***V* (Å^3)**	**wR$_2$**	**GOF**	**Reduced χ^2**
$Az_3Sb_2Cl_9$	0.000	0.000	8.5834	19.970	1274.2	6.96	2.16	4.67
$Az_3Sb_2Cl_{8.5}Br_{0.5}$	0.056	0.156	8.6110	20.063	1288.4	10.5	1.80	3.24
$Az_3Sb_2Cl_8Br$	0.111	0.272	8.6533	20.156	1307.0	10.6	1.36	1.84
$Az_3Sb_2Cl_{7.5}Br_{1.5}$	0.167	0.409	8.6822	20.266	1323.0	12.2	1.51	2.27
$Az_3Sb_2Cl_7Br_2$	0.222	0.491	8.6989	20.321	1331.7	10.6	1.41	2.00
$Az_3Sb_2Cl_6Br_3$	0.333	0.526	Not single phase					
**$Az_3Sb_2Cl_7Br_2$(s)**	**0.222 (s)**	**0.533**	**8.6984**	**20.359**	**1334.0**	**8.92**	**2.79**	**7.77**
$Az_3Sb_2Cl_{4.5}Br_{4.5}$	0.500	0.751	8.7275	20.557	1356.1	11.4	2.21	4.88
$Az_3Sb_2Cl_3Br_6$	0.667	0.898	8.7579	20.759	1378.9	10.5	1.75	3.08
$Az_3Sb_2Cl_2Br_7$	0.778	0.928	8.7607	20.797	1382.3	9.92	1.81	3.28
$Az_3Sb_2Cl_1Br_8$	0.889	0.967	8.765	20.85	1387.	12.	2.2	5.00

			0	0	2	3	4	
$Az_3Sb_2Br_9$	1.000	1.000	8.769 3	20.89 8	1391. 8	8.9 9	2.5 3	6.41

As discussed in the main manuscript, the deviation from Vegard's law suggests a non-random distribution of Br and Cl maybe be present. In addition, the positive and negative deviations in the *a*- and *c*-axes, respectively suggest a specific preference of site(s) rather than clustering. The deviations from Vegard's law are most evident in compositions with near 50:50 Br:Cl; the sample $Az_3Sb_2Cl_7Br_2$(s) was found by EDS to have ~ 53:47 Br:Cl ratio (i.e. actual composition $Az_3Sb_2Cl_{4.2}Br_{4.8}$) and was selected to investigate any preferred site occupancy using Rietveld refinements of PXRD data. Initial refinements assumed homogeneous distributions of Br and Cl across all six crystallographic sites in a 0.533:0.467 ratio according to the EDS composition. As discussed in the main manuscript preferred orientation (PO) of the *c*-axis was included to account for the large observed intensities of (00L) reflections, and the refinement result is shown in Figure S3.11(a) and summarised in Table S3.7. The preferred orientation results in a good fit in intensity for the (002) reflection, but underestimates (004). This suggests that there is some contribution from Br preferred site occupancy on the structure factors, F(002) and F(004), for these reflections in addition to PO. However, attempts to freely refine site occupancies (while constraining global composition), resulted in unstable refinements due to the large number of refinable parameters. To investigate this further, two extreme models were considered. In the first model, preferred occupancy of Br at the terminal sites was investigated by exclusively occupying the bridging X2 sites by Cl leaving both Cl and Br at the terminal X1 and X3 sites with fractional occupancies of 0.2005 and 0.7995, respectively, to meet the required global composition. In the second model, Br was instead exclusive situated at the bridging X2 sites, leaving a Cl:Br in a 0.2005:0.7995 ratio at the terminal sites. In both cases these occupancies were fixed but all other parameters including PO were refined. The refinement profiles for these two models are shown in Figures S3.11 (b) and (c), respectively. It is evident that model 1 with preferred Br occupancy at the terminal sites, results in a significant underestimation of the intensity of the (002) peak and a small overestimation for the (004) peak, Figure S3.11 (b). Conversely, model 2, with Br exclusively at the bridging sites gives a dramatic improvement in the (002) intensity but underestimates the (004) peak, Figure S3.11 (c). This clearly indicates some slight site preference for Br to occupy the bridging sites. Further refinements of Br occupancy at the bridging X2 site were performed by manually varying the fractional occupancy (while maintaining global composition); the most satisfactory outcome was achieved for a Br occupancy of 0.613 at bridging X2 site and

0.493 at terminal X1 and X3 sites (Cl occupancy at bridging X2 and terminal X1 and X3 sites were 0.387 and 0.507, respectively), Figure S3.11 (d). The refinement of site occupancy slightly improves the refinement results with a reduction in wR_2 from 8.92 to 8.58 (c.f. Table S3.7); this suggests that there is a weak (approximate 15%) preferred occupancy of Br at the bridging X2 site in the $Az_3Sb_2Cl_7Br_2$(s) powder sample. This result is not entirely unexpected as Br at the bridging sites would provide better screening of Sb-Sb interactions, and this may also explain the observed deviations from Vegard's law.

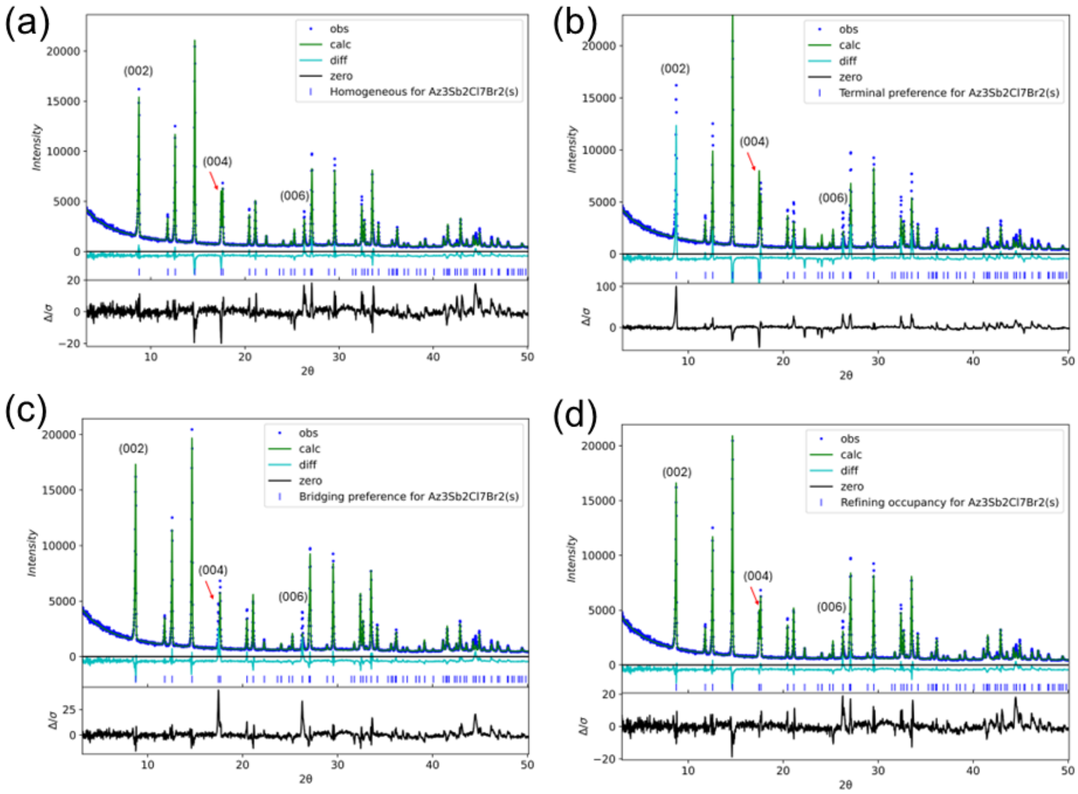

Figure S3.11 GSAS-II refinement profiles for PXRD data of $Az_3Sb_2Cl_7Br_2$(s) using: (a) a homogeneous distribution of halide ions (i.e. fractional occupancies of 0.533:0.467 Br:Cl at all sites); preferred Br occupancy at (b) terminal X1 and X3 sites or (c) bridging X2 sites; and (d) 15% Br preference for X2 bridging sites (Br X2 fractional occupancy of 0.613).

PXRD of the $Az_3Sb_2Cl_6Br_3$ powder sample shows two isostructural phases with $P6_3mc$ space group, Figure S3.12. From the refinement results the main phase occupies ca. 90 wt% of the total sample and tends to be Br rich, while the minor phase tends to be

Cl rich. Full refinements for both phases are difficult because only a few peaks are observable and refinable for the Cl rich phases.

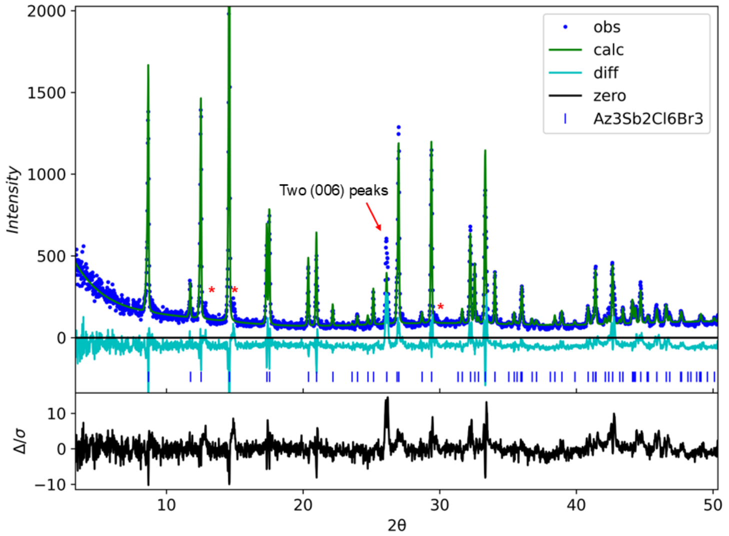

Figure S3.12 GSAS-II refinement profile for PXRD data at room temperature for $Az_3Sb_2Cl_6Br_3$ with two *P*6_3*mc* phases.

S3.4 Energy-dispersive X-ray spectroscopy (EDS) results

EDS analysis was employed to study the chemical compositions and distributions of solid solution $Az_3Sb_2(Cl_{1-x}Br_x)_9$. Typical powder morphology of samples based on backscattered electron imaging is shown in Figure S3.13. Sampling of EDS was performed using both rastering over large areas spanning numerous crystallites and also spot analyses of individual crystals to determine the overall chemical composition and also distribution of Br:Cl. For each sample, at least 5 different large areas were selected for the determination of chemical composition, whilst at least 20 different small crystals were used to evaluate any local variation in Br:Cl ratio - the results are summarised in Table S3.8.

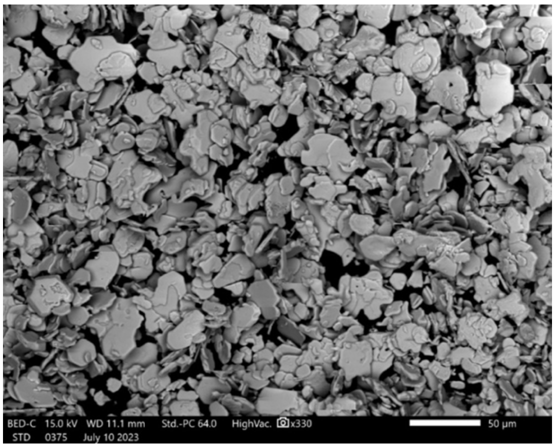

Figure S3.13 Scanning electron microscope backscattered electron imaging for $Az_3Sb_2Cl_7Br_2$(s) powder sample.

Table S3.8 EDS analysis for solid solution $Az_3Sb_2(Cl_{1-x}Br_x)_9$

Target composition	EDS composition.	Br proportion during synthesis	actual Br proportion (by EDS)	error Br content
$Az_3Sb_2Cl_9$	$Az_3Sb_2Cl_9$	0.000	0.000	0.000
$Az_3Sb_2Cl_{8.5}Br_{0.5}$	$Az_3Sb_2Cl_{7.60}Br_{1.40}$	0.056	0.156	0.029
$Az_3Sb_2Cl_8Br$	$Az_3Sb_2Cl_{6.55}Br_{2.45}$	0.111	0.272	0.028
$Az_3Sb_2Cl_{7.5}Br_{1.5}$	$Az_3Sb_2Cl_{5.32}Br_{3.68}$	0.167	0.409	0.036
$Az_3Sb_2Cl_7Br_2$	$Az_3Sb_2Cl_{4.58}Br_{4.42}$	0.222	0.491	0.035
$Az_3Sb_2Cl_6Br_3$	$Az_3Sb_2Cl_{4.27}Br_{4.73}$	0.333	0.526	0.039
$Az_3Sb_2Cl_7Br_2$(s)	$Az_3Sb_2Cl_{4.21}Br_{4.79}$	0.222 (s)	0.533	0.049
$Az_3Sb_2Cl_{4.5}Br_{4.5}$	$Az_3Sb_2Cl_{2.24}Br_{6.76}$	0.500	0.751	0.032
$Az_3Sb_2Cl_3Br_6$	$Az_3Sb_2Cl_{0.91}Br_{8.09}$	0.667	0.898	0.038
$Az_3Sb_2Cl_2Br_7$	$Az_3Sb_2Cl_{0.64}Br_{8.36}$	0.778	0.928	0.029
$Az_3Sb_2Cl_1Br_8$	$Az_3Sb_2Cl_{0.30}Br_{8.70}$	0.889	0.967	0.028
$Az_3Sb_2Br_9$	$Az_3Sb_2Br_9$	1.000	1.000	0.000

S3.5 Dielectric spectroscopy results

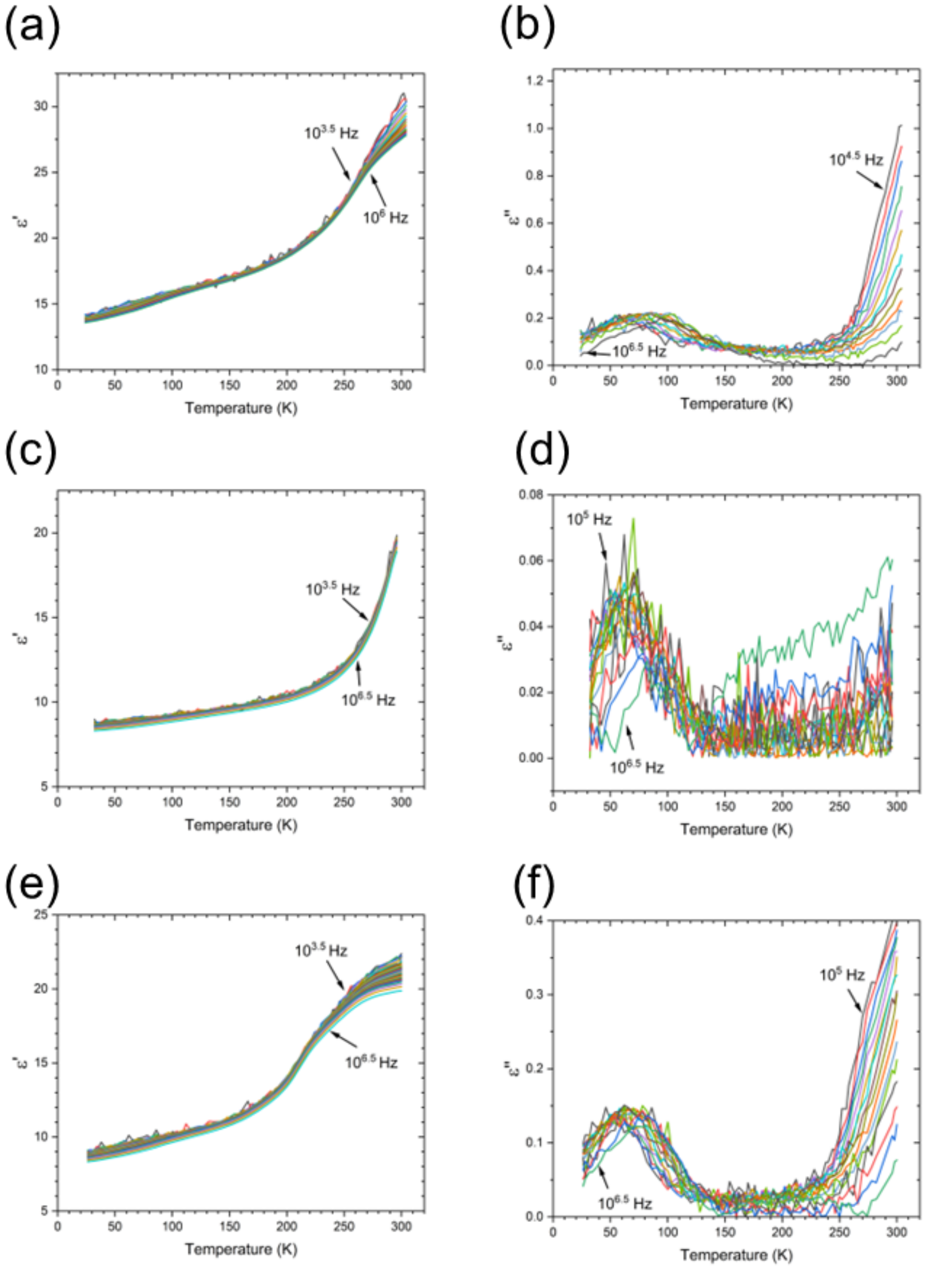

Figure S3.14 Dielectric data collected on cooling, showing both real, ε' and imaginary, ε'' parts of the complex dielectric permittivity as a function of frequency (ω) and temperature (T) for (a,b) for $Az_3Sb_2I_9$, (c,d) $Az_3Bi_2Br_9$, and (e,f) $Az_3Bi_2I_9$.

S3.6 DSC and DTA results

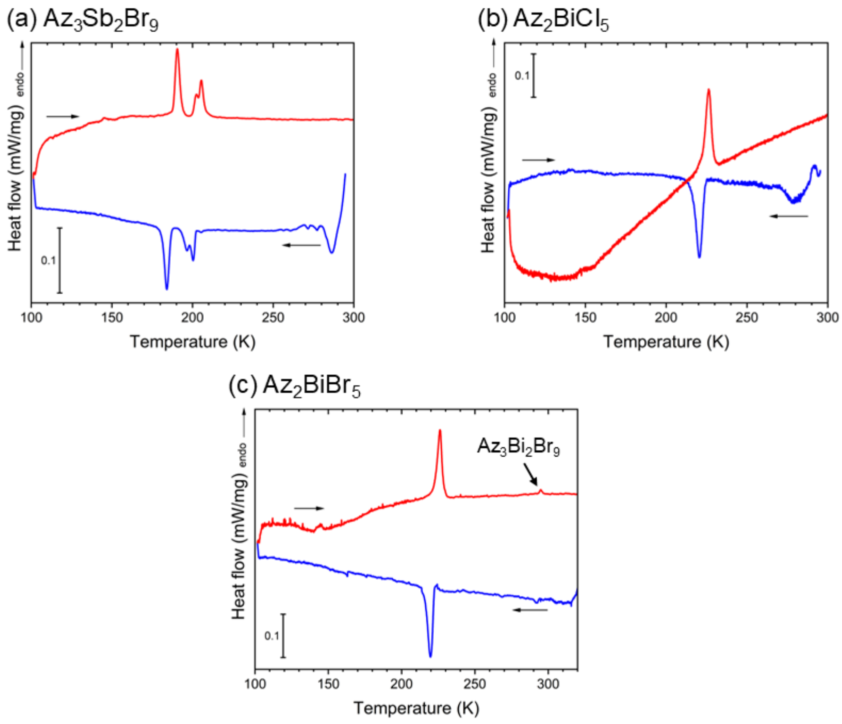

Figure S3.15 DSC curves for (a) $Az_3Sb_2Br_9$, (b) Az_2BiCl_5 and (c) Az_2BiBr_5 with a heating/cooling rate of 10 K min^{-1}.

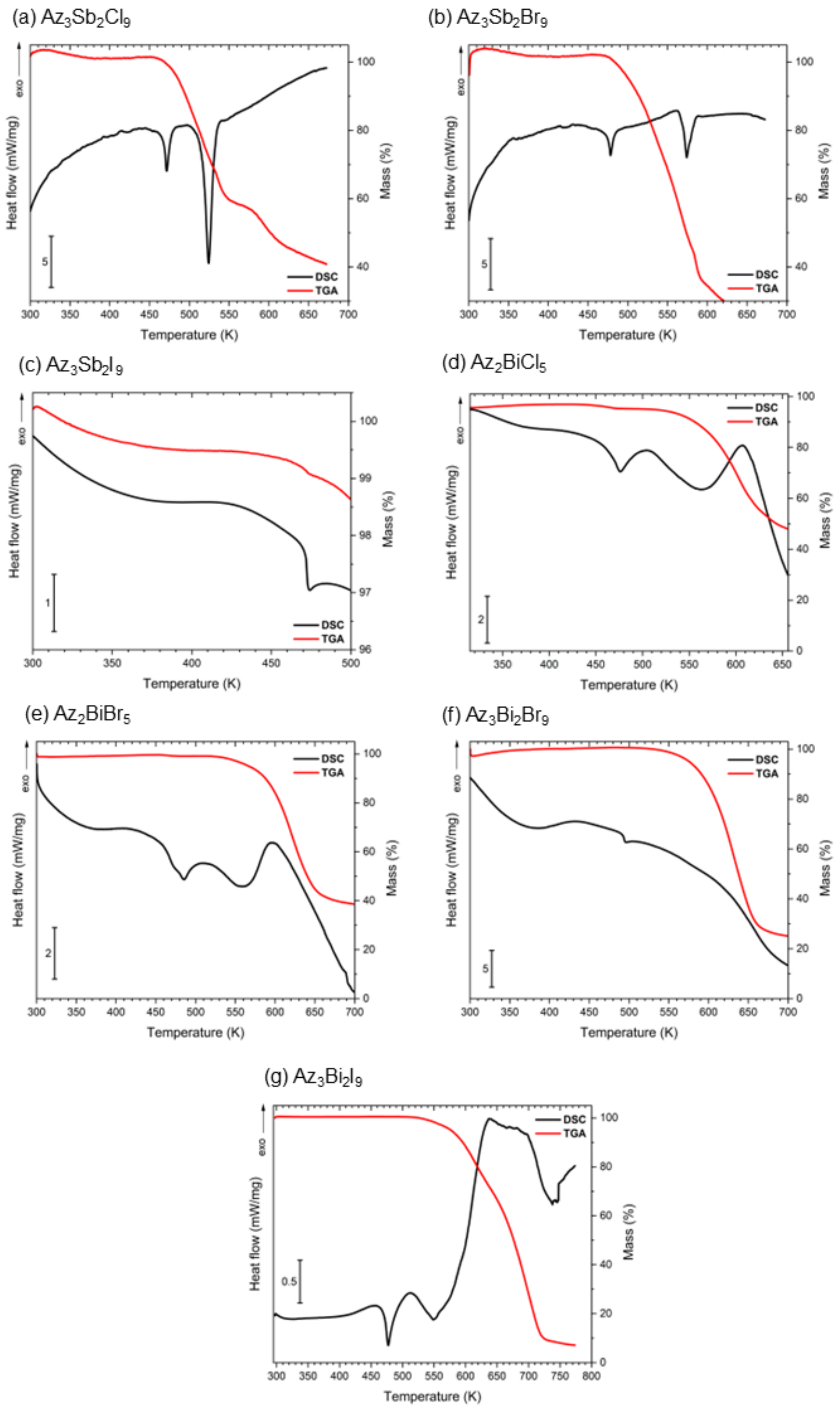

Figure S3.16 DTA-TG curves for (a) $Az_3Sb_2Cl_9$, (b) $Az_3Sb_2Br_9$, (c) $Az_3Sb_2I_9$, (d) Az_2BiCl_5, (e) Az_2BiBr_5, (f) $Az_3Bi_2Br_9$ and (g) $Az_3Bi_2I_9$ with a heating rate of 10 K min^{-1}.

Table S3.9 Onset temperature (T_{onset}), enthalpy (ΔH), and entropy change (ΔS) for successive transitions in $Az_nB_mX_{n+3m}$ series compounds.

**Compound**	**Transition**	**T_{onset} (K)**		**ΔH (kJ mol^{-1})**		**ΔS (J K^{-1} mol^{-1})**		
		Heating	Cooling	Heating	Cooling	Heating	Cooling	average
$Az_3Sb_2Cl_9$	I to II	213.2	220.2	4.12	3.92	19.35	17.81	18.58
$Az_3Sb_2Br_9$	I to II	202.1	201.5	0.98	0.70	4.84	3.47	4.16
	II to III	198.5	197.2	0.62	0.38	3.11	1.91	2.51
	III to IV	186.8	186.7	1.98	1.94	10.60	10.38	10.49
$Az_3Sb_2I_9$	I to II	289.1	278.0	5.78	3.72	20.01	13.39	16.70
Az_2BiCl_5	I to II	219.6	225.1	2.54	2.78	11.58	12.35	11.97
$Az_3Bi_2Br_9$	I to II	294.2	294.2	7.72	8.26	26.25	28.09	27.17
	II to III	195.4		1.48		7.60		
Az_2BiBr_5	impurity	293.4	293.7	0.08	0.09	0.26	0.32	0.29
	I to II	222.5	222.4	3.29	3.24	14.79	14.59	14.69
$Az_3Bi_2I_9$	I to II	237.8	229.1	4.13	3.37	17.38	14.71	16.04
	II to III	172.8		0.58		3.35		

S3.7 Optical absorbance results

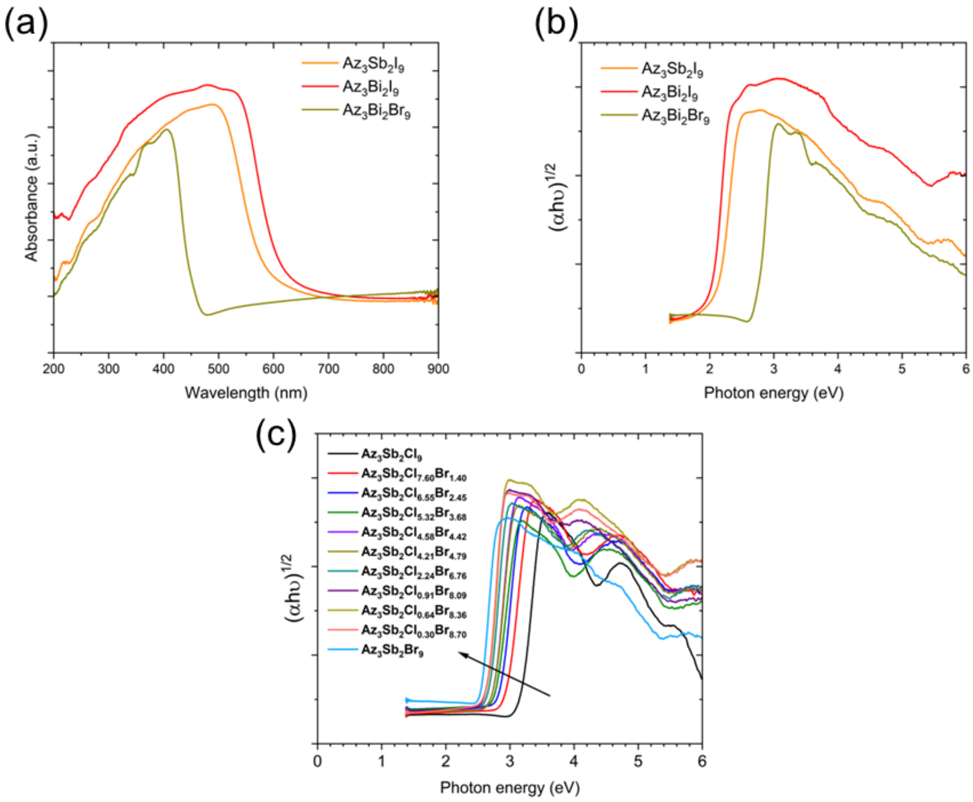

Figure S3.17 (a) Optical absorbance spectra for $Az_3Sb_2I_9$, $Az_3Bi_2I_9$ and $Az_3Bi_2Br_9$, Tauc plot assuming indirect band gap for (b) $Az_3Sb_2I_9$, $Az_3Bi_2I_9$ and $Az_3Bi_2Br_9$ and (c) $Az_3Sb_2Cl_{9-x}Br_x$.

S3.8 Computational results based on DFT

DFT calculations on 2D (hcc)$_2$ perovskite structure for $Az_3Sb_2Cl_9$ and $Az_3Sb_2Br_9$

The computational results indicate an indirect band gap for both $Az_3Sb_2Cl_9$ and $Az_3Sb_2Br_9$. The projected density of states (pDOS) diagram of both compounds suggests that the top of the valence band is dominated by the halogen p orbitals, whereas the bottom of the conduction band primarily consists of Sb 5p orbital contributions, as expected. The corresponding VBM and CBM originate mainly from the electronic states of the Sb and halogen rather than the surrogate A-site cesium ion. The indirect band gap estimated from total DOS suggests 2.13 and 1.70 for $Az_3Sb_2Cl_9$ and $Az_3Sb_2Br_9$, respectively, which are significantly underestimated compared to the experimental values of 3.23 and 2.59 eV, which is possibly caused by the PBE functionals. It is worth noting that the indirect band gap for $Az_3Sb_2Cl_9$ is in contrast to the DFT calculations of Luo *et al.*[14] This might be related to the octahedral distortion

induced by the use of a different A-site cation. In their work, a hypothesized organic cation was established to solve the symmetry incompatibility problem arising from lower symmetric Az^+ cation. In this study, the all A-site organic cations were replaced by Cs^+ cations in the DFT calculation for simplification.

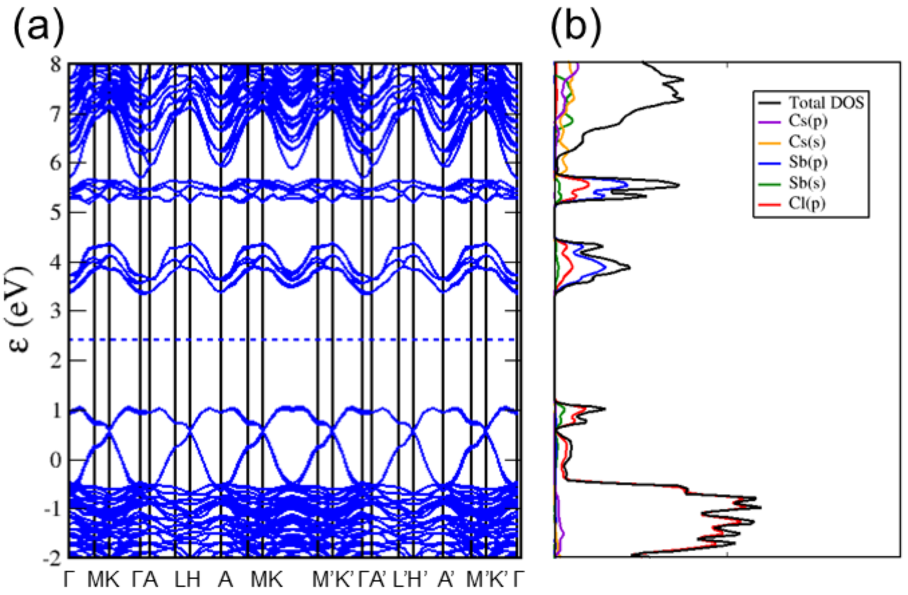

Figure S3.18 (a) Calculated band structure for $Az_3Sb_2Cl_9$ without spin-orbit coupling and (b) corresponding pDOS

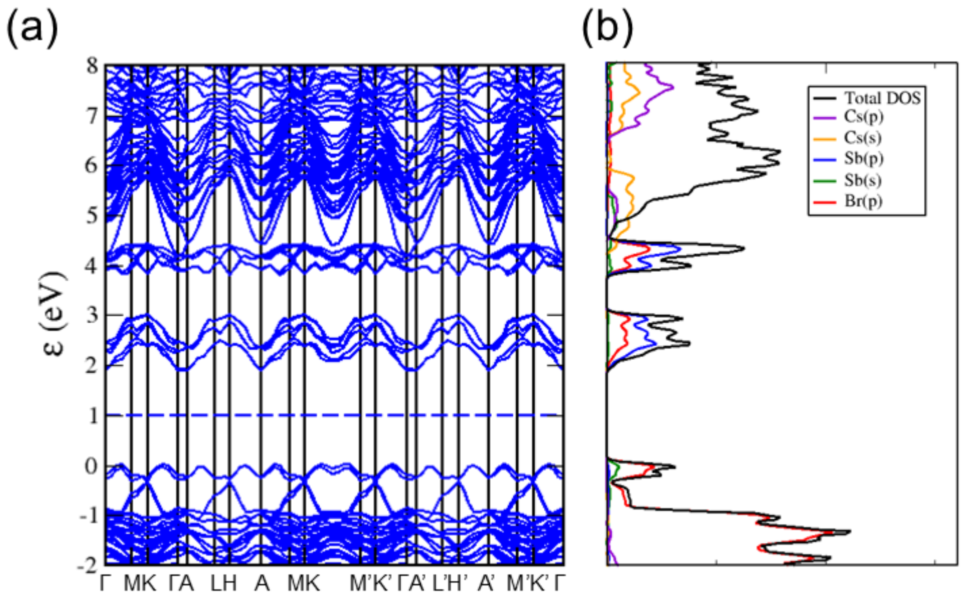

.

Figure S3.19 (a) Calculated band structure for $Az_3Sb_2Br_9$ without spin-orbit coupling and (b) corresponding pDOS

DFT calculations on 0D $(hcc)_2$ perovskite structure in $Az_3Sb_2I_9$, $Az_3Bi_2Br_9$ and $Az_3Bi_2I_9$

The band structure and projected density of states calculated by DFT for $Az_3Sb_2I_9$, $Az_3Bi_2I_9$ and $Az_3Bi_2Br_9$ are shown in Figure S3.20, Figure S3.21 and Figure S3.22 respectively. To simplify calculations, the primitive cells of the *Cmcm* phases determined from SCXRD at RT were used and without considering spin-orbit coupling. From the calculated band structure, all dimer structures show typical indirect band gaps for the room temperature phase. The pDOS all show a similar characteristic to the layered analogues where the highest valence states are predominately constituted by the halogen p states and the pnictogen s states, and the lowest energy conduction band states are mainly dominated by the pnictogen p states. The calculated band gaps from total DOS were 2.30, 2.04 and 2.88 eV, respectively for $Az_3Sb_2I_9$, $Az_3Bi_2I_9$ and $Az_3Bi_2Br_9$. The large difference in the calculated band gap and optical band gap in $Az_3Bi_2I_9$ and $Az_3Bi_2Br_9$ is possibly caused by neglecting spin-orbital coupling using PBE functional which was discussed in the similar study of $Cs_3Bi_2I_9$.[15]

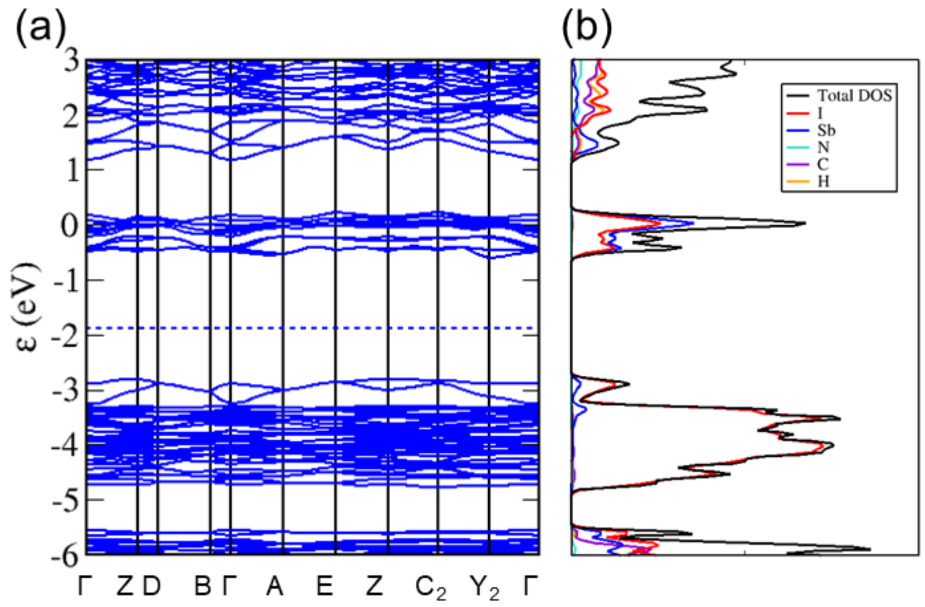

Figure S3.20 (a) Calculated band structure for $Az_3Sb_2I_9$ without spin-orbit coupling and (b) corresponding pDOS

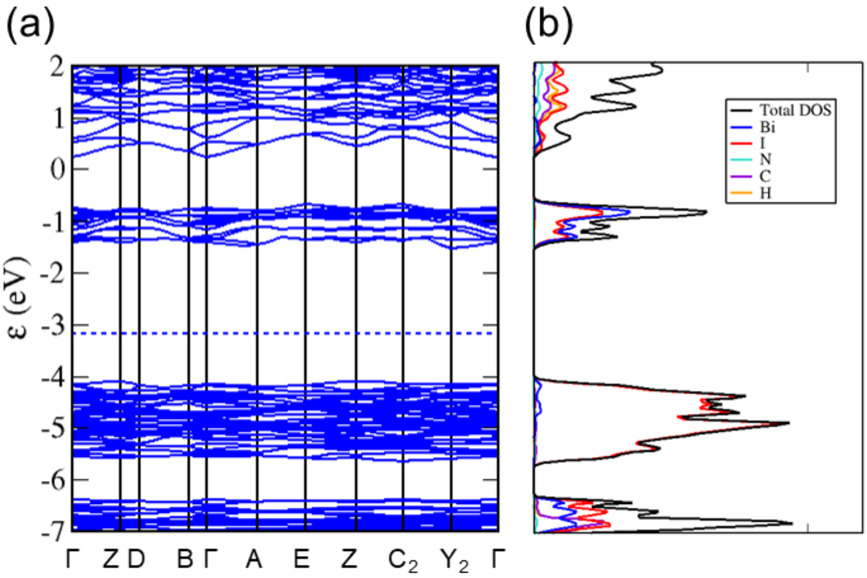

Figure S3.21 (a) Calculated band structure for $Az_3Bi_2I_9$ without spin-orbit coupling and (b) corresponding pDOS

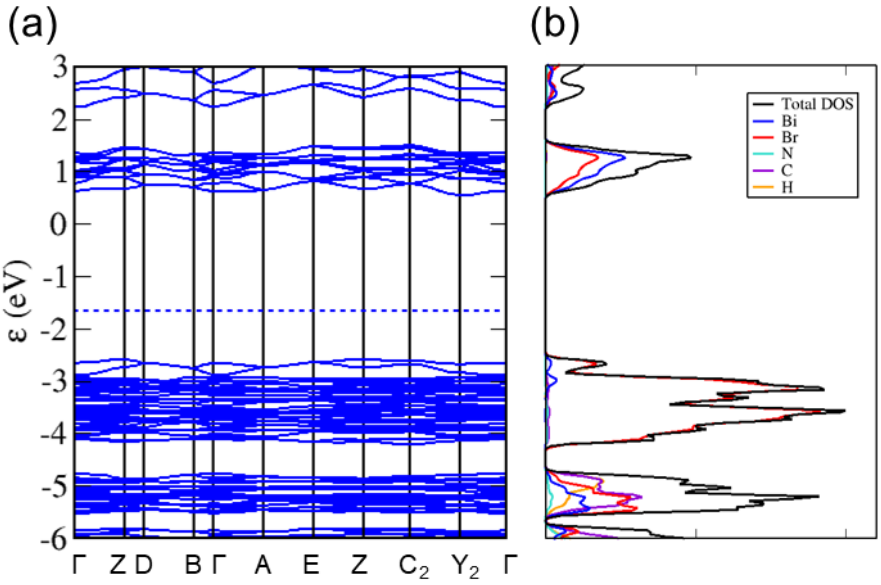

Figure S3.22 (a) Calculated band structure for $Az_3Bi_2Br_9$ without spin-orbit coupling and (b) corresponding pDOS

S4. Supplementary Discussions

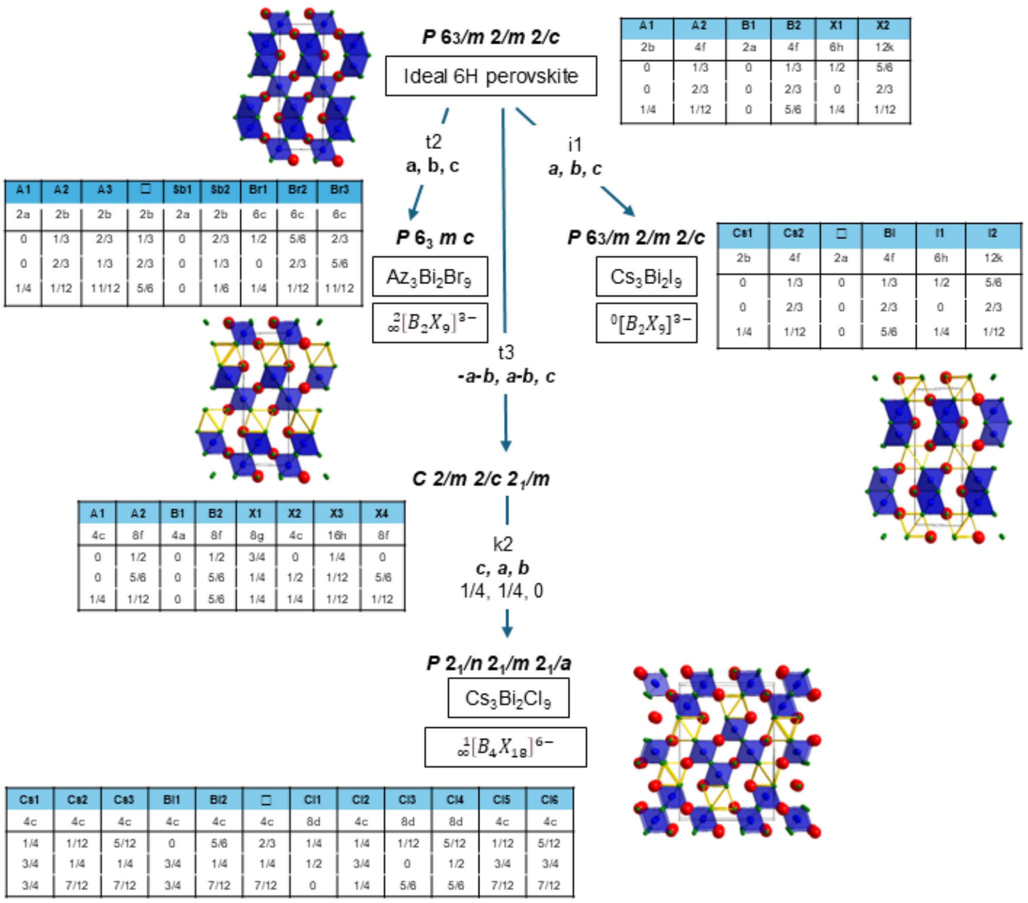

Figure S4.1 Full version of the Bärnighausen tree for the group-subgroup relationship among the aristotype structure of ideal 6H closed packed B-site deficient $A_3B_2X_9$ family where *t* represents translation equivalent subgroups, *k* denotes point group equivalent subgroups and *i* denotes isomorphic structure.

S4.1 Discussion on crystal structures

Chemical environment of the inorganic asymmetric units in $Az_3Sb_2Cl_9$

The phase transition from $P6_3mc$ phase I to $Pna2_1$ phase II in $Az_3Sb_2Cl_9$ leads to significant change in the chemical environment of the inorganic asymmetric units (Figure S4.2). The distortion of polyhedra is generally quantified by polyhedral distortion parameters. In this study, polyhedral distortion index and bond angle variance were used to evaluate the octahedral distortion as shown in Table S4.1 and S4.2. The polyhedral distortion index based on bond lengths is defined as:

$$D = \frac{1}{n}\sum_{i=1}^{n}\frac{|l_i - \bar{l}|}{\bar{l}}$$

where l_i represents the distance between the central atom and the *i*th coordinated atom and $\bar{l}$ denotes the average bond length.[16] The bond angle variance σ^2 for a coordination octahedron can be calculated by:

$$\sigma^2 = \frac{1}{11}\sum_{i=1}^{12}(\phi_i - 90)^2$$

where ϕ_i denotes the *i*th bond angle.[17] As shown in Figure S4.2, PT I to II results in a significant distortion of the $[SbCl_6]^{3-}$ octahedra, quantified by the increase of bond angle variance from 0.96 and 9.03 to 30.08 and 16.46 for Sb1 and Sb2, respectively. A summary of the octahedral distortion indices calculated by VESTA 3 are shown in Tables S4.1-3 for all compounds.[18]

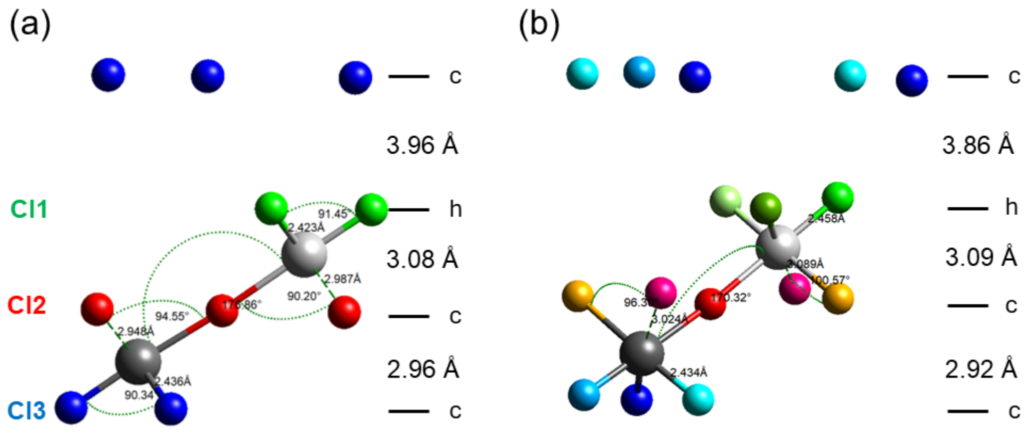

Figure S4.2 Chemical environment of the Sb octahedra in (a) $P6_3mc$ (phase I) at 293K and (b) $Pna2_1$ (phase II) for $Az_3Sb_2Cl_9$ at 173K

Table S4.1 Polyhedral distortion parameters for $Az_3Sb_2Cl_9$ and $Az_3Sb_2Br_9$ measured at RT, 173 K by SCXRD

	$Az_3Sb_2Cl_9$				$Az_3Sb_2Br_9$	
Space group	$Pna2_1$		$P6_3mc$		$P6_3mc$	
T (K)	173		293		293	
Crystallographic sites	Sb1	Sb2	Sb1	Sb2	Sb1	Sb2
Average bond length (Å)	2.702	2.713	2.705	2.692	2.844	2.846
Polyhedral volume (Å^3)	25.94	26.41	26.37	25.89	30.63	30.68
Distortion index	0.101	0.102	0.104	0.095	0.089	0.083
Bond angle variance (deg.^{2})	30.08	16.46	0.96	9.03	2.26	3.60

Table S4.2 Polyhedral distortion parameters for $Az_3Sb_2I_9$, $Az_3Bi_2Br_9$ and $Az_3Bi_2I_9$ measured at RT, 173K and 100K by SCXRD

	$Az_3Sb_2I_9$		$Az_3Bi_2Br_9$			$Az_3Bi_2I_9$		
Space group	*Cmcm*	*Pbcm*	*Cmcm*	*Pbcm*	*Pnma*	*Cmcm*	*Pbcm*	*Pnma*
T (K)	293	173	293	173	100	293	173	100
Average bond length (Å)	3.055	3.050	2.879	2.887	2.888	3.105	3.102	3.102
Polyhedral volume (Å^3)	37.76	37.57	31.44	31.71	31.67	39.55	39.47	39.36
Distortion index	0.055	0.057	0.056	0.059	0.055	0.047	0.050	0.047
Bond angle variance (deg.^{2})	14.71	16.01	26.66	28.04	31.99	20.46	19.43	24.34

S5. References

1. CrysAlisPro v1.171.42.74a, a., 96a, and 43.109a Rigaku Oxford Diffraction, Rigaku Corporation, Tokyo, Japan 2023.
2. Sheldrick, G. M., A short history of SHELX. *Acta Crystallogr., A, Found. Crystallogr.* **2008,** *64* (1), 112-122.
3. Sheldrick, G. M., Crystal structure refinement with SHELXL. *Acta crystallogr., C Struct. Chem.* **2015,** *71* (1), 3-8.
4. Dolomanov, O. V.; Bourhis, L. J.; Gildea, R. J.; Howard, J. A.; Puschmann, H., OLEX2: a complete structure solution, refinement and analysis program. *J. Appl. Crystallogr.* **2009,** *42* (2), 339-341.
5. Toby, B. H.; Von Dreele, R. B., GSAS-II: the genesis of a modern open-source all purpose crystallography software package. *J. Appl. Crystallogr.* **2013,** *46* (2), 544-549.
6. Viezbicke, B. D.; Patel, S.; Davis, B. E.; Birnie, D. P., Evaluation of the Tauc method for optical absorption edge determination: ZnO thin films as a model system. *Phys. Status Solidi B* **2015,** *252* (8), 1700-1710.
7. Makula, P.; Pacia, M.; Macyk, W., How To Correctly Determine the Band Gap Energy of Modified Semiconductor Photocatalysts Based on UV-Vis Spectra. *J. Phys. Chem. Lett.* **2018,** *9* (23), 6814-6817.
8. Clark, S. J.; Segall, M. D.; Pickard, C. J.; Hasnip, P. J.; Probert, M. I.; Refson, K.; Payne, M. C., First principles methods using CASTEP. *Z. Kristallogr. Cryst. Mater.* **2005,** *220* (5-6), 567-570.
9. Hinuma, Y.; Pizzi, G.; Kumagai, Y.; Oba, F.; Tanaka, I., Band structure diagram paths based on crystallography. *Comput. Mater. Sci.* **2017,** *128*, 140-184.
10. Jin, Y. U.; Marler, B.; Karabanov, A. D.; Winkler, K.; Yap, I. C. J.; Dubey, A.; Spee, L.; Escobar Castillo, M.; Muckel, F.; Salak, A. N.; Benson, N.; Lupascu, D. C., Lead-free organic-inorganic azetidinium alternating metal cation bromide: $[(CH_2)_3NH_2]_2AgBiBr_6$, a perovskite-related absorber. *RSC Adv.* **2023,** *13* (51), 36079-36087.
11. Tian, J.; Cordes, D. B.; Quarti, C.; Beljonne, D.; Slawin, A. M. Z.; Zysman-Colman, E.; Morrison, F. D., Stable 6H Organic–Inorganic Hybrid Lead Perovskite and Competitive Formation of 6H and 3C Perovskite Structure with Mixed A Cations. *ACS Appl. Energy Mater.* **2019,** *2* (8), 5427-5437.
12. Campbell, B. J.; Stokes, H. T.; Tanner, D. E.; Hatch, D. M., ISODISPLACE: a web-based tool for exploring structural distortions. *J. Appl. Crystallogr.* **2006,** *39* (4), 607-614.
13. H. T. Stokes, D. M. H., and B. J. Campbell, ISODISTORT, ISOTROPY Software Suite, iso.byu.edu., ISODISTORT, ISOTROPY Software Suite.
14. Luo, W.; Wu, L. K.; Shen, H. Y.; Li, H. K.; Xu, Z. J.; Shi, C.; Ye, H. Y.; Miao, L. P.; Wang, N., Halogen-Regulated T_c and X-ray Radiation Detection in 2D

Hybrid Perovskite Ferroelastic Semiconductor. *Inorg. Chem.* **2024,** *63* (8), 3913-3920.
15. Ghosh, B.; Chakraborty, S.; Wei, H.; Guet, C.; Li, S.; Mhaisalkar, S.; Mathews, N., Poor Photovoltaic Performance of $Cs_3Bi_2I_9$: An Insight through First-Principles Calculations. *J. Phys. Chem. C* **2017,** *121* (32), 17062-17067.
16. Baur, W., The geometry of polyhedral distortions. Predictive relationships for the phosphate group. *Acta Crystallogr. B* **1974,** *30* (5), 1195-1215.
17. Robinson, K.; Gibbs, G.; Ribbe, P., Quadratic elongation: a quantitative measure of distortion in coordination polyhedra. *Science* **1971,** *172* (3983), 567-570.
18. Momma, K.; Izumi, F., VESTA 3 for three-dimensional visualization of crystal, volumetric and morphology data. *J. Appl. Crystallogr.* **2011,** *44* (6), 1272-1276.
